# Gene Instability-Related lncRNA Prognostic Model of Melanoma Patients via Machine Learning Strategy

**DOI:** 10.1155/2021/5582920

**Published:** 2021-05-25

**Authors:** Kexin Yan, Yutao Wang, Yining Shao, Ting Xiao

**Affiliations:** ^1^Department of Dermatology, The First Hospital of China Medical University, National Health Commission Key Laboratory of Immunodermatology, Key Laboratory of Immunodermatology of Ministry of Education, Shenyang, Liaoning, China; ^2^Department of Urology, China Medical University, The First Hospital of China Medical University, Shenyang, Liaoning, China; ^3^Department of Plastic Surgery, China Medical University, The First Hospital of China Medical University, Shenyang, Liaoning, China

## Abstract

**Background:**

Melanoma is a common tumor characterized by a high mortality rate in its late stage. After metastasis, current treatment methods are relatively ineffective. Many studies have shown that long noncoding RNA (lncRNA) may participate in gene mutation and genomic instability in cancer.

**Methods:**

We downloaded transcriptome data, mutation data, and clinical follow-up data of melanoma patients from The Cancer Genome Atlas. We divided samples into groups according to the number of somatic cell mutations and then performed a differential analysis to screen out the differentially expressed genes. We then divided samples into genomic unstable and genomic stable groups. We compared lncRNA expression profiles in these groups and constructed a protein-coding genes network coexpressed with selected lncRNA to analyze the pathways enriched by these genes. Two machine learning methods, least absolute shrinkage and selector operation (LASSO) and support vector machine-recursive feature elimination (SVM-RFE), were applied to conduct the lncRNA-related prognostic model. Afterward, we performed survival analysis, risk correlation analysis, independent prognostic analysis, and clinical subgroup model validation. Finally, through wound healing assay and transwell assay, the function of AATBC was verified by A375 cell lines.

**Results:**

We screened 61 prognostic-related lncRNAs and constructed an lncRNA-mRNA coexpression network based on these lncRNAs. Seven lncRNAs were selected as common characteristic factors based on the two machine learning methods. The model formula was as follows: risk score = 0.085*∗*AATBC + 0.190*∗* AC026689.1−0.117*∗*AC083799.1 + 0.036*∗* AC091544.6−0.039*∗* LINC01287−0.291*∗* SPRY4.AS1 + 0.056*∗* ZNF667.AS1. The seven lncRNAs in this formula are key candidates. Cell experiments have verified that knocking down AATBC in A375 cell lines can reduce the proliferation and invasion ability of melanoma cells.

**Conclusion:**

The lncRNA we identified provides a new way to study lncRNA's role in the genomic instability of melanoma. Our findings may provide essential candidate biomarkers for the diagnosis and treatment of melanoma.

## 1. Introduction

Melanoma is a malignant tumor that originates in melanocytes. The incidence of melanoma has increased in recent decades. Although most patients benefit from early diagnosis and treatment and have a good prognosis, advanced melanoma is associated with poor outcome [1]. Early melanoma can be treated surgically. After advanced metastasis, the main treatment methods are systemic chemotherapy and biochemical therapy [2]. Therefore, it is essential to identify melanoma genes that might improve diagnosis, treatment, and outcome.

About 93% of DNA is transcribed into RNA in the human genome, while only 2% encodes proteins. A large portion of the rest is transcribed into RNA encoding no proteins, the so-called noncoding RNAs. RNAs of more than 200 bases are called long noncoding RNA (lncRNA) [3]. Several lines of evidence showed that these lncRNAs are not junk or transcriptional noise; they have critical biological purposes. They participate in structural and regulatory functions in translation mechanisms [4]. The lncRNA mediates a wide range of biological functions through interactions with one or more protein chaperones [5]. The lncRNA is involved in many cell-signaling pathways and participates in the occurrence, development, and metastasis of cancer. The lncRNA may mediate carcinogenesis or tumor inhibition [6].

Genomic instability refers to the process by which the genome is prone to change or has an increased propensity to change. Genomic instability during cell division is associated with parental cells' inability to replicate the genome accurately and the precise distribution of genomic materials among their daughter cells. Tumorigenesis can be seen as the accumulation of genomic changes in cell division series [7]. Genomic instability has long been recognized as one of the tumorigenesis drivers and the source of treatment resistance. Numerous studies have shown that genomic changes found in cancer genomes are transcribed. Replication stress and oxidative stress contribute to genomic instability and subsequent genomic changes [8]. The instability of cancer genomes exacerbates the phenomenon of genetic heterogeneity within tumors. Andor et al. stated that high genomic instability might be the basis for tumor susceptibility to DNA damage therapy [9].

In addition to traditional experimental methods, bioinformatics is used to identify genes associated with disease and to build risk scoring models. In addition to the common model composed of mRNA, the model composed of lncRNA and miRNA has also attracted more and more attention. Liu et al. screened seven lncRNA signatures as prognostic markers for melanoma by comprehensively analyzing the competitive endogenous RNA network [10]. Zhu et al. constructed a global triple network and found that MALAT1 and LINC00943 may be closely related to melanoma occurrence [11]. Bao et al. and Geng et al. explored lncRNA signatures associated with gene instability in lung adenocarcinoma and breast cancer, respectively [12, 13]. Nevertheless, the relationship between lncRNA in melanoma and genetic instability was not discussed in these bioinformatic studies.

To study the lncRNA associated with genomic instability in melanoma patients, we combined the mRNA expression spectrum, lncRNA expression spectrum, somatic mutation spectrum, and clinical follow-up data from melanoma tumor genomes to establish the prognosis model of melanoma using the machine learning method. We also explored the possibility of using lncRNA signature as an indicator of genomic instability in melanoma.

## 2. Materials and Methods

### 2.1. Data Collection

TCGA-SKCM FPKM RNA matrix, clinical follow-up information, and somatic mutation information of melanoma patients were obtained from The Cancer Genome Atlas (TCGA) (https://portal.gdc.cancer.gov/) [14]. TCGA-SKCM long noncoding RNA expression matrix was obtained from the TANRIC database (http://bioinformatics.mdanderson.org/main/TANRIC:Overview, version 1.0.6) [15]. We retained 470 melanoma samples with paired long noncoding RNA and mRNA expression profiles, clinical follow-up information, and somatic mutation information for further study. We randomly allocated all patients with melanoma into training and validation sets. We used the training set to conduct the genome stability-related prognosis lncRNA signature. We used the validation set to verify the accuracy of the model in the training set.

### 2.2. Identification Genome Stability-Related lncRNAs

We calculated the number of somatic mutations in each sample. Based on the number of somatic mutations, we defined the 25% with the most somatic mutations as the high mutation group and the 25% with the least somatic mutations as the low mutation group. We conducted a differential analysis of the lncRNA of samples from the high and low mutation groups and determined the difference in lncRNA between the two groups according to |logFC>1|,  *P* < 0.05.

### 2.3. The Functional Analysis of lncRNA

To evaluate the relative biological functions of the differential lncRNA obtained above, we indirectly characterized the biological functions of these lncRNAs by constructing protein-coding genes coexpressed by lncRNA and enriching the functions of these coexpressed genes. After obtaining the coexpression network, we determined the top ten mRNAs related to lncRNA according to the Pearson correlation coefficient's size and included them in the subsequent functional analysis [16]. We analyzed Gene Ontology (GO) [17] and the Kyoto Encyclopedia of Genes and Genomes (KEGG) [18] using cluster-Profiler software in R 3.6.3. We verified the correlations between lncRNA and sample pathway scores and determined whether these lncRNAs were associated with pathways related to genome stability.

### 2.4. Feature Selection Based on Machine Learning

Univariate Cox regression analysis was performed based on these genomic mutation-related lncRNAs. After combining the lncRNAs selected by the LASSO [19] and SVM-RFE [20] algorithms, lncRNAs were selected simultaneously by the two algorithms. Using the seven lncRNAs, we segregated the 470 discovery-phase samples into gene stable and gene unstable clusters. We then used a multi-Cox regression model to further narrow down the lncRNA-based signature for patients with melanoma in the training cohort.

### 2.5. Cell Lines and Culture

The human melanoma A375 cell line was purchased from Fuheng Biology (Shanghai, China). They were previously stored in liquid nitrogen, and the frozen cells were quickly removed for cell resuscitation. It was cultured at 37°C, 5% CO_2_ medium with high glucose DMEM (Hyclone Laboratories Inc., Logan, UT, USA), and 10% fetal bovine serum (FBS) (Solely Biomall, Shanghai, China).

### 2.6. Cell Transfection

The medium was changed 6 hours after transfection. The SiRNA sequences were as follows: 5ʹ-CAUGCAGACUUCUACAUCA-3ʹ 5ʹ-GGACCCACGUGACCAUCAA-3ʹ.

### 2.7. Wound Healing Assay

Cells were seeded into 6-well culture plates. When the cells reached 80%–90% confluency, 2 scratches were evenly drawn in the Petri dish with 1000 *μ*L pipetting nozzle. The cells were then washed with phosphate buffer solution and incubated at 37°C in a low-serum medium of 3% FBS. After that, the picture was taken under a microscope, and the area of the scratch was calculated. After incubation for 24 hours, the wound area was calculated again.

### 2.8. Transwell Assay

Transfected A375 cells were inoculated on pretreated Matrigel using transwell chambers with 8 micron pores. Add 200 *μ*L serum-free medium and 600 *μ*L (10% FBS) medium to upper and lower chambers, respectively. After incubation at 37°C for 24 h, it was fixed with 4% paraformaldehyde and stained with 1.0% crystal violet. Finally, the EVOSTM XL Core Imaging System (Invitrogen; Thermo Fisher Scientific, Inc.) was used to observe the staining of cells and counted the invaded cells and processed the images using ImageJ software.

### 2.9. Statistical Analysis

We used Euclidean distances and Ward's linkage method to perform hierarchical cluster analyses between various lncRNA matrices [21]. We performed univariate Cox proportional hazard regression analysis to identify the independent prognostic value of the various lncRNAs. We performed a multivariate Cox proportional hazard regression analysis to conduct genome stability-related lncRNA signature. GILncSig = a1x1+a2x2+a3x3+,…, +anxn. The genome instability-derived lncRNA signature (GILncSig) is the overall survival risk score for patients with melanoma. Higher risk scores imply a greater risk of death in the same period. A1 is the coefficient of lncRNA in multivariate Cox analysis. For coefficients >0, the lncRNA is a risk factor; for coefficients <0, the lncRNA is a protective factor. X1 is the expression level of a particular lncRNA. We used the median GILncSig of the melanoma samples in the training cohort as the cutoff point to separate patients into various risk groups. We used the Kaplan–Meier method and the log-rank test to evaluate the survival difference between high and low-risk groups with a significance level of 5%. We drew time-dependent receiver operating characteristic (ROC) curves to evaluate the training and validation cohorts' prognosis status diagnostic ability. We used R-version 3.6.3 for all statistical analyses.

## 3. Results

### 3.1. Genome Stability-Related lncRNAs

**Figure 1:**
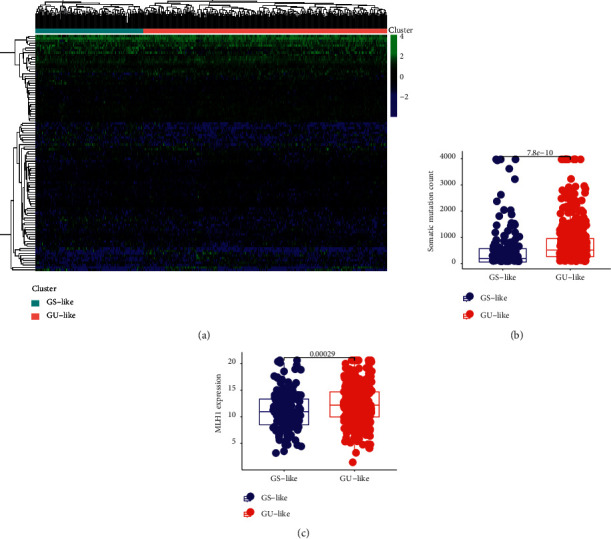
(a) Consensus cluster analysis divided the samples into the gene stable group and gene unstable group. (b) In somatic cell mutation count, the expression of the GS group and GU group was significantly different (*P* = 7.8e−10). (c) In MLH1 expression, the expression of the GS group and GU group was significantly different (*P* = 0.00029).

We matched the TCGA-SKCM melanoma cohort with the mutation burden. We selected the 25% samples with the most somatic mutations as the high mutation group and the 25% samples with the least number of cell mutations as the low mutation group. In the difference analysis of the matrix lncRNA, we obtained 214 statistically significant and significantly different lncRNAs (Table S1). To determine whether these top significant 100 differential lncRNAs are genomic stability-related variables in the overall sample, we conducted a consensus cluster analysis on the overall sample. We found that these lncRNAs divide the overall sample into a GS-like group and a GU-like group (Figure 1(a)). We determined that the number of somatic mutations (Figure 1(b)) and the expression of the critical gene MLH1 (Figure 1(c)) for mismatch repair differed significantly between the groups. These results show that the lncRNAs are related to genome stability [22].

### 3.2. lncRNA-mRNA Coexpression Network

**Figure 2:**
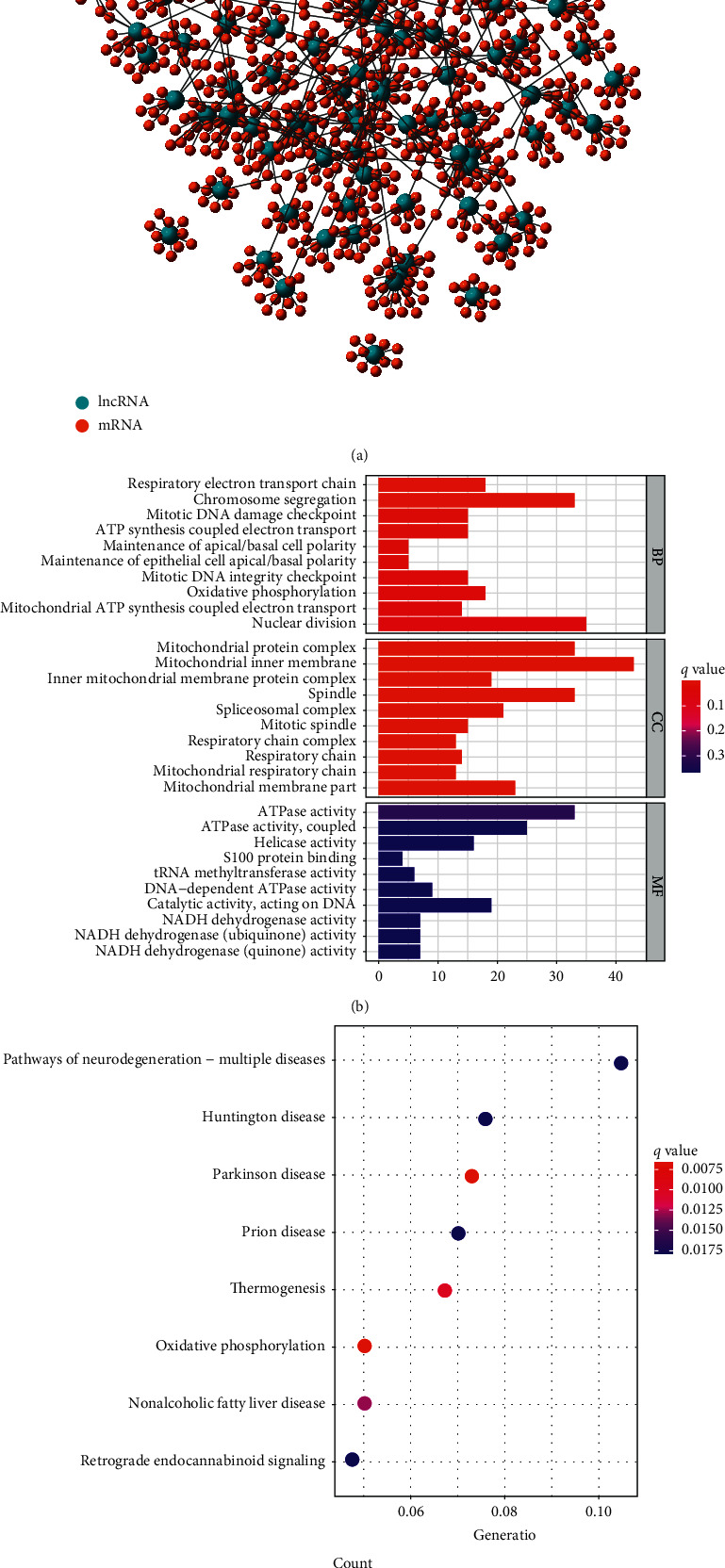
(a) Coexpression network of lncRNA-mRNA. Blue represents for lncRNA and red represents for mRNA. (b) GO analysis of the lncRNA-mRNA coexpression network. These candidate genes are related to biological processes such as respiratory electron transport chain, chromosome segregation, and mitotic DNA damage checkpoints. (c) KEGG analysis of the lncRNA-mRNA coexpression network. These candidate genes are related to oxidative phosphorylation.

The current understanding of the related functions of lncRNA is in the preliminary stages of development. Therefore, we have no way to use the existing database to perform a functional analysis of the 25 genomic stability-related lncRNAs. Therefore, we constructed a lncRNA-mRNA coexpression network (Figure 2(a)). Using functional analysis of the protein-coding genes related to the function of these lncRNAs, we indirectly speculated that these lncRNAs might participate in biology by regulating their coexpressed protein-coding genes in the process of network adjustment.

GO enrichment results showed that these protein-coding genes are related to biological processes such as respiratory electron transport chain, chromosome segregation, and mitotic DNA damage checkpoints (Figure 2(b)). KEGG enrichment results showed that these protein-coding genes are related to oxidative phosphorylation (Figure 2(c)).

### 3.3. Construction of a Genome Instability-Related lncRNA Risk Model

**Figure 3:**
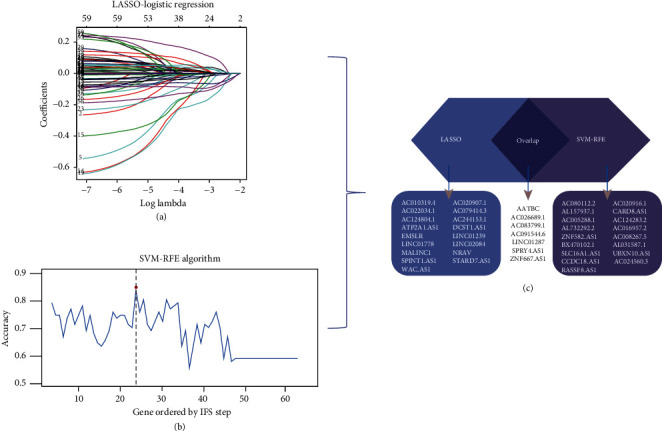
Feature selection of lncRNAs using two algorithms. (a) Results of Lasso regression analysis. (b) Results of SVM-RFE algorithm. (c) lncRNAs selected in LASSO and SVM-RFE algorithm were used for prognostic module.

**Figure 4:**
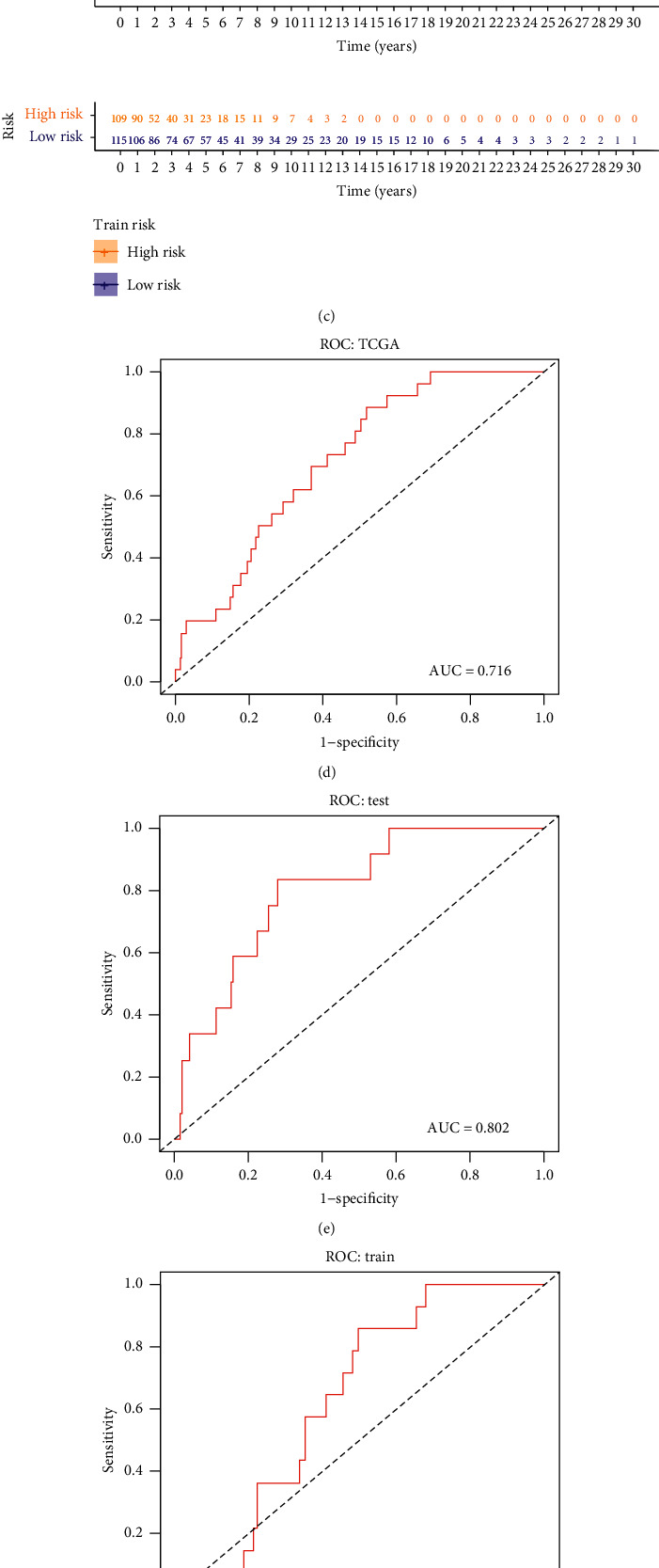
(a), (b), (c) The Cox proportional hazards regression model in all sample group (*P* < 0.001), train group (*P* < 0.001), and test group (*P* < 0.001) can reflect the difference of survival rate between the high-risk group and low-risk group. (d), (e) The area under the ROC curve of all sample group is 0.716. (f) The area under the ROC curve of the test group is 0.802. (g) The area under the ROC curve of the train group is 0.641. (h), (i) WRN expression was different in all sample group (*P* = 1.3e−08), test group (*P* = 8.7e−05), and train group (*P* = 1.6e−05).

On this basis, we first selected 61 lncRNAs using univariate Cox regression analysis. Then, we identified a group of 24 lncRNAs using the LASSO algorithm. Meanwhile, SVM-RFE algorithm was implemented, and another group of 24 lncRNAs was screened out. lncRNAs selected by LASSO and SVM-RFE algorithm were intersected, and a total of seven key lncRNAs were selected. The lncRNAs are as follows: AATBC, AC026689.1, AC083799.1, AC091544.6, LINC01287, SPRY4.AS1, and NF667.AS1 (Figure 3). Then, we calculated the risk score as follows using multivariate risk hazard regression analysis: risk score = 0.085*∗* AATBC + 0.190*∗* AC026689.1-0.117*∗* AC083799.1 + 0.036*∗* AC091544.6-0.039*∗* LINC01287-0.291*∗* SPRY4.AS1 + 0.056*∗* ZNF667.AS1 (Table 1). We performed a multivariate analysis of the signature and other clinical features of these gene instability-related lncRNAs to verify that its efficacy is independent of other clinical features (Table 2). The risk score model divides TCGA-SKCM samples into high-risk and low-risk groups based on the median score. The survival curve analysis demonstrated a significant difference in survival between the two groups (Figure 4(a); *P* < 0.001). Higher risk scores correlate with worse outcomes. We then obtained the same results in the training and validation sets (Figures 4(b) and 4(c)). We conducted risk model diagnosis tests on survival status in TCGA-SKCM, training, and validation sets. The diagnostic test results showed that the area under the ROC curve of the whole set, training set, and the validation set of TCGA-SKCM was 0.716, 0.641, and 0.802, respectively (Figures 4(d)–4(f)).

**Figure 5:**
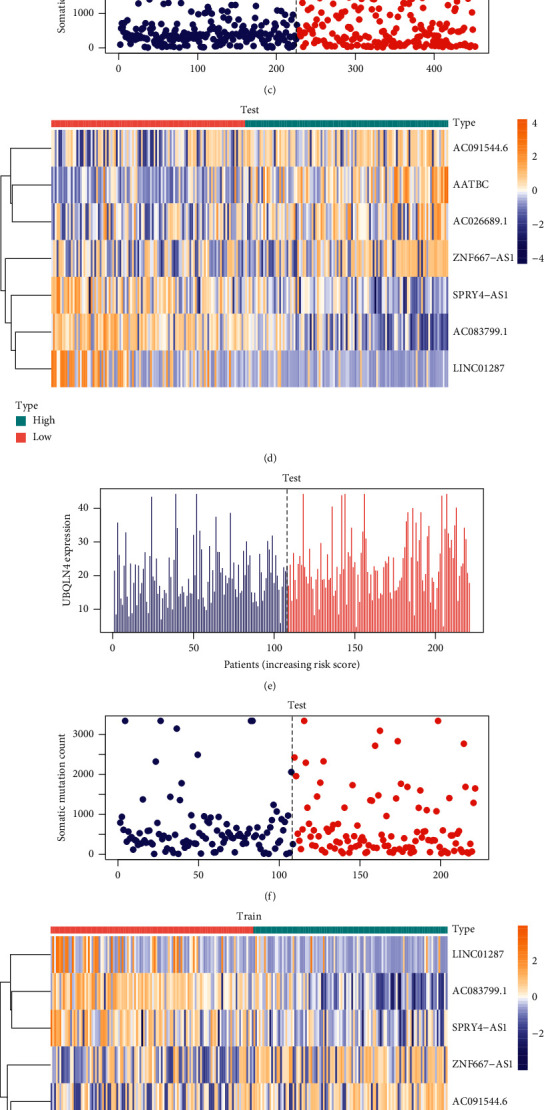
(a), (d), (g) Heat maps of the expression of key lncRNAs in high-risk and low-risk groups in all sample group, test group, and train group. (b), (e), (h) In all sample group, test group, and train group, the expression value of UBQLN4 corresponding to the risk score of patients. (c), (f), (i) In all sample group, test group, and train group, the somatic mutation count corresponding to the risk score of patients.

We already demonstrated the clinical prognostic value of lncRNA related to genome stability. Next, drew expression heat maps of the risk scoring model in each group and the corresponding number of somatic mutations and the expression levels of UBQLN4 (Figure 5) because UBQLN4 is a factor that indicates gene instability [23]. WRN RecQ-like helicase encodes a member of the RecQ subfamily of DNA helicase proteins [24]. The encoded nuclear protein is essential for maintaining genome stability and participates in DNA repair, replication, transcription, and telomere maintenance. Comparison analysis showed significant differences in WRN RecQ-like helicase expression pattern between the samples in the high-risk and low-risk groups (Figures 5(g)–5(i)). We found that the expression of WRN was significantly increased in the low-risk group (*P* < 0.001, Mann–Whitney *U*-test), suggesting that the genome is stable. The prognostic signature constructed by lncRNAs in this study should be combined with gene instability; therefore, we supplemented the characteristic difference analysis of four KEGG pathways associated with genomic instability (ssGSEA). The expression of these KEGG pathways was found to be higher in groups with higher risk scores (Figure S1).

### 3.4. Clinical Subgroup Model Validation

**Figure 6:**
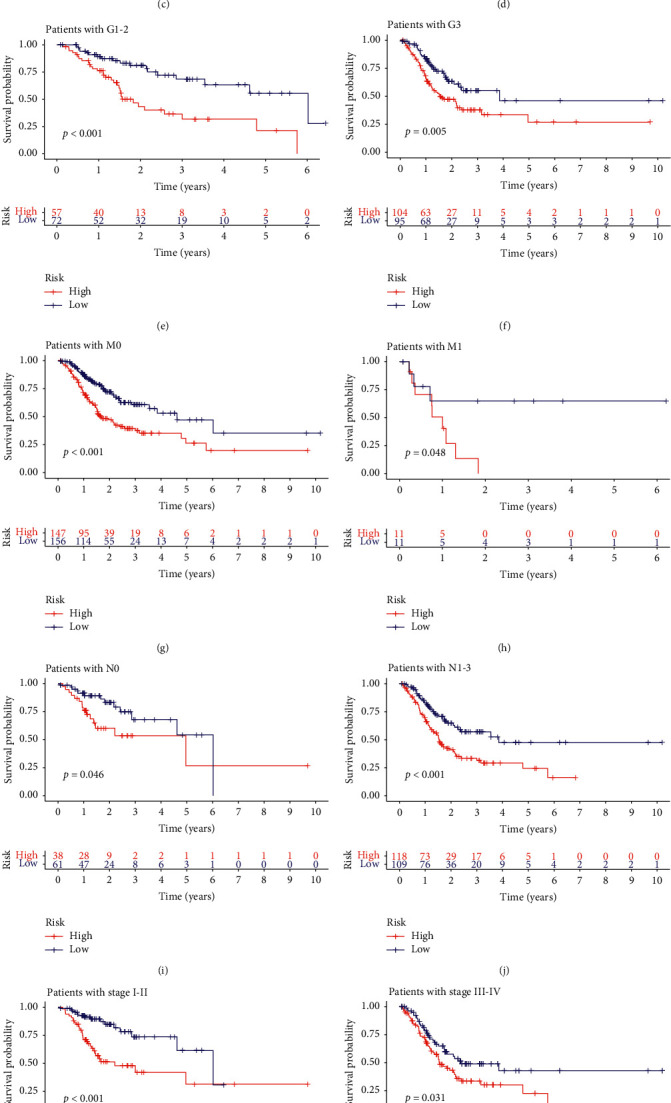
Model validation for clinical subgroups. The samples were grouped by age, sex, metastasis, stage, and lymph node infiltration. The red curve represents the high-risk group and the blue curve represents the low-risk group. The Cox proportional hazards regression model showed good predictive ability in each clinical subgroup. All of the results were statistically significant.

Based on this analysis, we determined a prognostic score model related to genome stability. To demonstrate its prognostic effect in various subgroups, we conducted a survival analysis. We found that grouping according to the scores of the sample documents, the prognostic model related to genomic stability significantly distinguished patients with different prognostic status (Figure 6).

### 3.5. Elimination of AATBC Can Inhibit the Proliferation and Migration of Melanoma Cells

**Figure 7:**
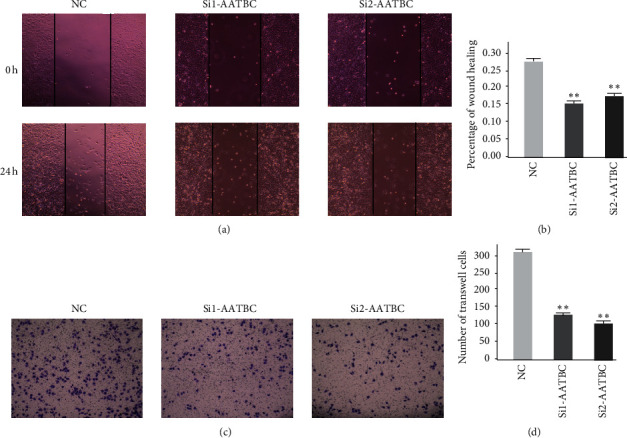
AATBC-related cell experiments. (a) The cell migration results of the normal control group, the SiRNA1 group, and the SiRNA2 group were observed under the microscope at 0 h and 24 h. (b) The results of wound healing assay were expressed as the mean ± SD of the three independent experiments (^*∗∗*^*P* < 0.01). (c) Transwell assay was used to compare the normal control group, the SiRNA1 group, and the SiRNA2 group. AATBC knockdown was observed to inhibit cell migration. (d) The results of transwell assay were expressed as the mean ± SD of the three independent experiments (^*∗∗*^*P* < 0.01).

In a series of in vitro experiments in A375 cell lines, we demonstrated that the overexpression of AATBC in melanoma plays an important role in poor prognosis. In this study, AATBC was knocked out, and a significant reduction in melanoma cell activity was observed (Figure 7). The results of wound healing assay and transwell assay also showed that the elimination of AATBC could inhibit the migration and invasion of A375 cells.

## 4. Discussion

**Figure 8:**
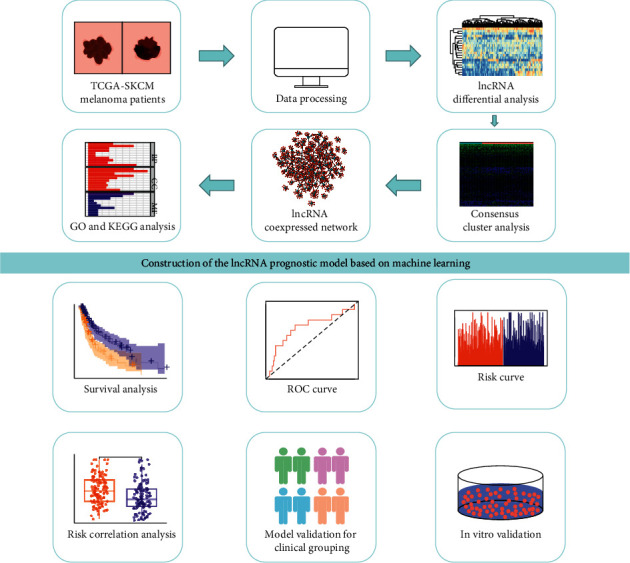
Flow chart of this study. First, RNA expression data, clinical follow-up data, and gene mutation data of melanoma patients were downloaded from the TCGA database. After data processing, two groups of samples were screened out according to the number of somatic cell mutations, and the differences were analyzed. According to the results of the difference analysis, the consensus cluster analysis was carried out on the total samples, and the samples were divided into the gene stable group and the gene unstable group. Then, a coexpression network of lncRNA-mRNA was constructed, and GO analysis and KEGG analysis were performed for this network. The machine learning method, Lasso regression analysis, and SVM-RFE method were combined to screen out key lncRNAs. Cox proportional hazards regression model was established, and key lncRNAs were selected. For this model, survival analysis, clinical subgroup analysis, risk correlation analysis, and in vitro validation were performed.

We downloaded gene transcriptome data, gene mutation data, and clinical follow-up data of melanoma patients from TCGA. Based on the somatic cell mutation number of melanoma patients, we screened the top 25% and the last 25% for differential analysis, and we screened out 25 differentially expressed lncRNAs. Using consensus cluster analysis, we divided all samples divided into an unstable genomic group (GU) and a genomic stable group (GS). After constructing the coexpression network of lncRNA-mRNA, we analyzed the pathways enriched in the network. Univariate Cox regression analysis was used to initially screen lncRNAs, and then, LASSO regression and SVM-RFE, two machine learning methods, were combined to select the key lncRNAs. After establishing an lncRNA-related multivariate Cox proportional risk regression model, we performed survival analysis, risk correlation analysis, independent prognostic analysis, clinical subgroup model validation, and in vitro validation according to selected vital factors to determine whether the model had good predictive ability (Figure 8).

Using GO analysis, we found that these lncRNAs are enriched in biological processes, including nuclear division, mitotic DNA damage checkpoint, chromosome segregation, and mitotic DNA integrity checkpoint. We know that genomes need to replicate precisely when cells divide and pass genetic material to their offspring. Changes that occur during DNA repair, chromosome replication, or recombination provide a natural genetic variation source. This low-frequency inherent variability of the genome is called genomic instability [25]. Such unstable events may be associated with chromosome loss, total chromosome rearrangement, copy number variation, and other genetic changes. Faulty DNA synthesis and defective excision or mismatch repair lead to genetic mutations. Chromosome misclustering leads to abnormal gain or loss of chromosomes during mitosis and chromosome number changes, also known as chromosome instability [26]. Cell cycle checkpoints detect DNA damage and regulate the cell cycle to ensure that the critical phase of the cell cycle is completed before entering the next phase and ensure the integrity of chromosomes [27]. In eukaryotes, cell cycle checkpoints ensure the coordination of DNA synthesis and DNA repair with cell division. The checkpoint monitors the DNA integrity, and if the DNA is damaged, it triggers a checkpoint reaction that stops the cell cycle from moving forward until the damage is repaired. Some tumors inactivate checkpoint responses [28].

For more than a century, people have used chemotherapy to treat cancer. Radiation and DNA-destroying drugs have been routine cancer treatments until now [29]. Anticancer chemotherapy causes genotoxic damage and activates molecular factors that regulate cell cycle checkpoints, leading to cell death and tumor regression. Antimitotic chemotherapy affects mitotic cells and interferes with normal mitotic processes, including spindle formation [30].

Their enrichment's cell components include the respiratory chain, mitochondrial inner membrane, mitochondrial protein complex, and the spindle body. Using KEGG analysis, we found that these lncRNAs were concentrated in thermogenesis and oxidative phosphorylation. The respiratory chain, also known as the electron transport chain, comprises a series of electronic carriers. It is a continuous reaction system consisting of a series of hydrogen and electron transfer reactions. It gives the pair of hydrogen atoms removed from the metabolite to oxygen to form water, and the energy released enables adenosine diphosphate and phosphate to form adenosine triphosphate. The coupling mechanism between electron transport and ATP formation is called oxidative phosphorylation (OxPhos). The respiratory chain progressively releases this energy, facilitating ATP and the maintenance of transmembrane potentials. Prokaryotic cells' respiratory chain is located on the plasma membrane, while eukaryotic cells are located on the inner membrane of mitochondria [31, 32].

At the beginning of the previous century, Otto Warburg observed that cancer cells obtain their energy from aerobic glycolysis by converting glucose into lactic acid. Warburg hypothesized that this was due to abnormal mitochondrial function in cancer cells. Hypoxia conditions present in many solid tumors may not satisfy their need for oxygen, allowing cancer cells to inhibit oxidative phosphorylation and promote glycolytic activation. The activation of oncogenes or inactivation of tumor suppressor genes may also increase glycolytic proteins [33]. Many recent studies showed that oxidative phosphorylation is upregulated in various cancers, possibly making them sensitive to inhibition of oxidative phosphorylation, thereby reducing tumor hypoxia. Many well-tolerated and widely prescribed drugs, including metformin, carboxylic aminotriazole, arsenic trioxide, and atroquinine, act as oxidative phosphorylation inhibitors and have the potential to act as anticancer therapeutics [34]. Investigators proposed several strategies to inhibit oxidative phosphorylation for treating cancer, including inhibiting mitochondrial transfer from stromal cells to malignant cells, inhibiting mitochondrial protein synthesis, using drugs that disrupt mitochondrial function, and directly inhibiting respiratory chain complexes [35].

Oxidative phosphorylation and glycolysis have essential roles in malignant tumor cells. Metabolic phenotypes in melanoma also show some metabolic plasticity between glycolysis and oxidative phosphorylation [36]. To maintain their function and proliferation, melanoma cells typically transfer their metabolism from mitochondria to glycolytic ATP production. Various oncogenes and tumor suppressors, as well as hypoxia, stimulate mitochondrial metabolism. A key oncogenic factor in melanoma is the mutation of the BRAF gene. This protein kinase participates in RAS-RAF-MEK-ERK mitogen-activated protein kinase signal transduction [37]. Despite the success of BRAFV600E inhibitors, the treatment response in patients with metastatic melanoma remains transient due to resistance acquired. Roesch et al. conducted cytotoxic therapy on melanoma cells and found that the deletion of JARID1B increased melanoma treatment sensitivity. Inhibition of the mitochondrial respiratory chain prevents the JARID1B^high^ subtype and improves multiple drug resistance in melanoma [38].

A careful literature search revealed that the biological functions of AC026689.1, AC083799.1, AC091544.6, SPRY4.AS1, and NF667.AS1 had not been reported to date. lncRNA AATBC is overexpressed in bladder cancer tissues and positively correlated with tumor grade and stage [39]. lncRNA AATBC was reported to promote the occurrence and development of nasopharyngeal carcinoma by regulating pinin through the mir-1237-3P-PNN-ZEB1 axis [40]. Mo et al. reported that the expression level of LINC01287 was increased in both hepatocellular cancer cell lines and tissues, and downregulation of LINC01287 could inhibit the growth of hepatocellular cancer cells [41]. In addition, Song et al. found that LINC01287 also promoted the proliferation and metastasis of breast cancer cells [42].

**Figure 9:**
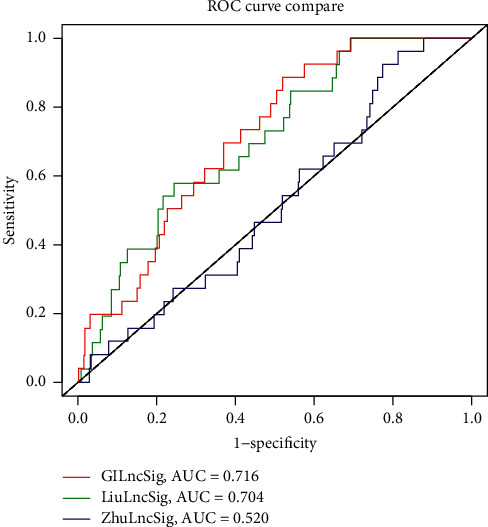
Comparison of models' ROC curve. The gene instability-related signature proposed in this study is compared with the model proposed by Zhu et al. and the model proposed by Liu et al. The model proposed in this study (AUC = 0.716) has the highest AUC value and the best predictive ability.

In order to compare the advantages of the lncRNA signature associated with gene instability proposed in this study, we compared it with the lncRNA-related prognostic model proposed by other scholars [10, 11]. In this study, the proposed area under curve (AUC) value of GIsig is 0.716, that of Liusig is 0.704, and that of Zhusig is 0.520 (Figure 9). It can be seen from the figure that the model proposed in this study has a higher AUC value and better predictive ability.

In summary, we combined machine learning method and other different bioinformatic mining analysis methods to verify literature mining results and found that the model we established measured indicators of genomic instability of melanoma patients and predicted outcomes. We found that AATBC, AC026689.1, AC083799.1, AC091544.6, LINC01287, SPRY4.AS1, and NF667.AS1 were biomarkers for genomic instability of melanoma. This provides an essential basis for the diagnosis and treatment of melanoma.

## Figures and Tables

**Table 1:** The prognosis model established by multivariate risk ratio regression analysis.

lncRNA	Coef	*P* value	HR
AATBC	0.085	0.093	1.089
AC026689.1	0.190	<0.001	1.210
AC083799.1	−0.117	<0.001	0.889
AC091544.6	0.036	0.020	1.037
LINC01287	−0.039	0.010	0.962
SPRY4.AS1	−0.291	0.003	0.747
ZNF667.AS1	0.056	0.005	1.057

**Table 2:** Variables in the equation.

	B	SE	Wald	Df	Sig	Exp (B)
Age	0.012	0.005	5.144	1	0.023	1.012
Gender	−0.028	0.168	0.027	1	0.869	0.973
Stage	0.278	0.116	5.768	1	0.016	1.320
T	0.187	0.079	5.600	1	0.018	1.205
M	0.488	0.474	1.062	1	0.303	1.629
Risk score	0.785	0.135	33.629	1	6.6698E-9	2.192

Df, degree of freedom; Sig, significance.

## Data Availability

The dataset used to support the findings of this study was downloaded from the open-source database TCGA.

## Supplementary Materials

Supplementary Materials Supplemental information for this article can be found online. Figure S1: the following four KEGG pathways (ssGSEA) associated with gene instability were analyzed for characteristic differences between the high- and low-risk score groups: base excision repair, DNA replication, homologous recombination, and mismatch repair. ^*∗∗∗∗*^*P* < 0.0001. ^*∗∗*^*P* < 0.01. ^*∗*^*P* < 0.05. Table S1: the difference analysis of the matrix lncRNA.
