# Supplementary material for: Gene Instability-Related lncRNA Prognostic Model of Melanoma Patients via Machine Learning Strategy
Source: J Oncol. 2021 May 25;2021:5582920. doi: 10.1155/2021/5582920 (PMC8169244; doi:10.1155/2021/5582920)
Supplement: Supplementary Materials — Supplemental information for this article can be found online. Figure S1: the following four KEGG pathways (ssGSEA) associated with gene instability were analyzed for characteristic differences between the high- and low-risk score groups: base excision repair, DNA replication, homologous recombination, and mismatch repair. ∗∗∗∗P < 0.0001. ∗∗P < 0.01. ∗P < 0.05. Table S1: the difference analysis of the matrix lncRNA. [file 5582920.f1.zip › 5582920.f1/supplementary table 1.docx]

| Supplementary table 1. The difference analysis of the matrix lncRNA. | | | | | |
| --- | --- | --- | --- | --- | --- |
| lncRNA | conMean | treatMean | logFC | pValue | fdr |
| AC120049.1 | 0.594275919 | 0.329897595 | -0.849114661 | 2.14E-09 | 3.19E-06 |
| PTPRG-AS1 | 0.390577317 | 0.668828493 | 0.776028144 | 8.97E-09 | 6.70E-06 |
| AP001453.4 | 1.050425828 | 1.476292209 | 0.491004013 | 1.34E-07 | 6.36E-05 |
| AC025048.2 | 0.623658973 | 1.03740465 | 0.73414948 | 1.70E-07 | 6.36E-05 |
| AC005330.1 | 1.01394683 | 0.293992271 | -1.786131872 | 3.18E-07 | 9.50E-05 |
| HOXD-AS2 | 2.181389399 | 1.39142198 | -0.648687312 | 5.83E-07 | 0.00014506 |
| AL139260.1 | 0.780722836 | 1.248397269 | 0.677194733 | 7.54E-07 | 0.000160849 |
| LINC02600 | 0.899250016 | 0.365053442 | -1.300614598 | 1.04E-06 | 0.000193334 |
| AC124804.1 | 1.315495502 | 0.421148565 | -1.643205159 | 1.39E-06 | 0.000222508 |
| ALMS1-IT1 | 0.389228304 | 0.584301336 | 0.586095963 | 1.49E-06 | 0.000222508 |
| AL133415.1 | 1.012367992 | 1.511019333 | 0.577788319 | 2.31E-06 | 0.000313499 |
| LINC00294 | 3.289940077 | 4.466034306 | 0.440933026 | 2.92E-06 | 0.000354877 |
| AL512353.1 | 0.469080143 | 0.732119602 | 0.642244921 | 3.09E-06 | 0.000354877 |
| AL161772.1 | 0.733307075 | 0.408451506 | -0.844252656 | 5.09E-06 | 0.000524942 |
| AL117327.1 | 0.646718069 | 1.041241261 | 0.687095563 | 5.45E-06 | 0.000524942 |
| AC010442.1 | 8.502682727 | 3.771269934 | -1.172867689 | 5.63E-06 | 0.000524942 |
| ABALON | 0.423769601 | 0.672498155 | 0.666250208 | 6.74E-06 | 0.000592232 |
| DSCR8 | 0.965560057 | 2.691063932 | 1.478738764 | 7.14E-06 | 0.000592232 |
| AP005233.2 | 0.709001367 | 1.370087666 | 0.950407893 | 8.76E-06 | 0.000643439 |
| MIR4435-2HG | 2.64429446 | 3.784646267 | 0.517275625 | 9.07E-06 | 0.000643439 |
| AP003119.2 | 1.239794216 | 0.666312395 | -0.895830042 | 9.21E-06 | 0.000643439 |
| AL138756.1 | 0.513417929 | 0.331342067 | -0.631812298 | 9.48E-06 | 0.000643439 |
| AL121772.1 | 0.351928375 | 0.805817521 | 1.195171334 | 1.21E-05 | 0.000788485 |
| LINC01503 | 1.375308517 | 0.782391735 | -0.813792253 | 1.28E-05 | 0.000796542 |
| LINC00973 | 0.386866752 | 1.471310899 | 1.92719348 | 1.36E-05 | 0.000815082 |
| RGMB-AS1 | 0.432925537 | 0.69112966 | 0.674837489 | 1.94E-05 | 0.001088104 |
| AP001065.3 | 0.793555627 | 0.556753083 | -0.511293714 | 1.97E-05 | 0.001088104 |
| AC005041.3 | 0.91677328 | 1.357922828 | 0.56676459 | 2.35E-05 | 0.001251198 |
| CHROMR | 2.106291337 | 2.707819716 | 0.362426689 | 2.95E-05 | 0.001480802 |
| LINC01128 | 1.316282405 | 1.791006482 | 0.444301511 | 2.98E-05 | 0.001480802 |
| UBXN10-AS1 | 1.298522539 | 0.762070508 | -0.768874666 | 3.40E-05 | 0.001614818 |
| LINC02585 | 0.770621644 | 1.110640149 | 0.527296842 | 3.46E-05 | 0.001614818 |
| AC019080.5 | 0.735995771 | 1.043460052 | 0.503605987 | 3.70E-05 | 0.001674137 |
| MCF2L-AS1 | 1.311080856 | 0.941360854 | -0.477936896 | 3.97E-05 | 0.001742274 |
| TDRKH-AS1 | 0.375706129 | 0.525043174 | 0.482831408 | 4.74E-05 | 0.002023983 |
| AC244153.1 | 1.212797265 | 0.670235584 | -0.855598215 | 5.19E-05 | 0.002153772 |
| AL513327.1 | 0.468341336 | 0.688706306 | 0.55632851 | 5.64E-05 | 0.00227423 |
| NUTM2A-AS1 | 1.269767698 | 1.561072423 | 0.297972887 | 6.42E-05 | 0.002473906 |
| AC046168.2 | 2.434493886 | 1.555293674 | -0.646434858 | 6.46E-05 | 0.002473906 |
| AC010980.2 | 0.863054047 | 0.493869992 | -0.805319597 | 6.90E-05 | 0.002576716 |
| LINC02761 | 1.552504263 | 0.655621293 | -1.243662614 | 7.43E-05 | 0.002703161 |
| AC008736.1 | 2.400174529 | 1.801659832 | -0.413812671 | 7.60E-05 | 0.002703161 |
| AC006946.2 | 0.707807908 | 1.093136332 | 0.627043554 | 8.37E-05 | 0.002906551 |
| ZNF710-AS1 | 1.593085454 | 1.171302658 | -0.443709748 | 8.85E-05 | 0.003003711 |
| DUXAP8 | 0.347137786 | 0.538954844 | 0.634655992 | 0.000108758 | 0.003608354 |
| DDX11-AS1 | 0.404961981 | 0.550610838 | 0.443246539 | 0.000114012 | 0.003700421 |
| KDM7A-DT | 3.511982479 | 2.534556364 | -0.4705524 | 0.000117646 | 0.003737135 |
| LBX2-AS1 | 9.295893582 | 12.32368551 | 0.406768313 | 0.000123303 | 0.003835229 |
| AL118522.1 | 0.677094031 | 0.368552539 | -0.877485906 | 0.000126349 | 0.003849781 |
| LINC02454 | 1.537481478 | 2.445455239 | 0.669534027 | 0.000130601 | 0.003899731 |
| AL392172.1 | 4.890629246 | 6.153429194 | 0.331370523 | 0.000139667 | 0.004088689 |
| MRPS30-DT | 0.466024613 | 0.666994465 | 0.517268636 | 0.000144067 | 0.004121675 |
| AC012073.1 | 0.998010766 | 1.347079906 | 0.432708147 | 0.000146315 | 0.004121675 |
| AATBC | 0.832058774 | 0.6855305 | -0.279464585 | 0.000184267 | 0.005002 |
| SLC16A1-AS1 | 0.429996756 | 0.591521074 | 0.46010379 | 0.000184267 | 0.005002 |
| AP003119.3 | 0.515160102 | 0.306782241 | -0.747805893 | 0.000191428 | 0.005014083 |
| AP001062.1 | 0.915421248 | 0.764819073 | -0.259317279 | 0.000191428 | 0.005014083 |
| AC002401.4 | 0.337892543 | 0.832574167 | 1.301014285 | 0.000199633 | 0.005138817 |
| AC016394.2 | 1.044497997 | 1.433889909 | 0.457124535 | 0.000206544 | 0.005226616 |
| CEROX1 | 6.360422511 | 3.921347585 | -0.697773078 | 0.000211293 | 0.005257674 |
| AC009486.1 | 0.347382946 | 0.550659593 | 0.664633819 | 0.000225095 | 0.005509298 |
| TBC1D8-AS1 | 0.473538379 | 0.615486501 | 0.378245859 | 0.000253147 | 0.00609594 |
| KDM4A-AS1 | 0.548909072 | 0.73506384 | 0.421302369 | 0.000274823 | 0.006512867 |
| LNCTAM34A | 1.48897211 | 1.126365763 | -0.402641344 | 0.000296028 | 0.006905789 |
| XIST | 1.59934607 | 0.728726155 | -1.134033469 | 0.000309427 | 0.007107289 |
| AC141002.1 | 0.363535408 | 0.488214808 | 0.425420166 | 0.000341765 | 0.007731128 |
| AL121772.3 | 0.698444902 | 1.154046869 | 0.724483601 | 0.00035333 | 0.007873461 |
| LINC01505 | 0.918213606 | 1.359741223 | 0.566430398 | 0.000363835 | 0.007930457 |
| MIR222HG | 0.876703885 | 1.298460634 | 0.56664073 | 0.000366511 | 0.007930457 |
| ATP2A1-AS1 | 2.432702155 | 3.730736871 | 0.616900914 | 0.000374637 | 0.007940477 |
| HOXA11-AS | 1.361322505 | 0.704629951 | -0.950071185 | 0.000380145 | 0.007940477 |
| U52111.1 | 0.827356549 | 1.15334533 | 0.479243446 | 0.00038293 | 0.007940477 |
| AC006449.7 | 1.313286913 | 1.604873098 | 0.289277088 | 0.000445967 | 0.009120935 |
| FLVCR1-DT | 0.824735756 | 1.119784424 | 0.441217156 | 0.00045245 | 0.009128489 |
| AC068580.3 | 0.864331233 | 1.240727483 | 0.521530073 | 0.00048273 | 0.009609541 |
| AC108463.2 | 0.422459852 | 0.711250106 | 0.751542719 | 0.000492909 | 0.009683075 |
| AC112220.2 | 0.897590792 | 1.188039013 | 0.404452431 | 0.000526048 | 0.010156735 |
| EP300-AS1 | 0.432720023 | 0.636235474 | 0.556126933 | 0.000535501 | 0.010156735 |
| FOXD3-AS1 | 7.783756115 | 9.759541347 | 0.326346842 | 0.000537429 | 0.010156735 |
| HEIH | 18.1593143 | 14.18781876 | -0.356056922 | 0.000572966 | 0.010581271 |
| AC011944.1 | 0.319453469 | 0.549254766 | 0.781869678 | 0.000574519 | 0.010581271 |
| LOXL1-AS1 | 0.640491881 | 0.442399134 | -0.533831721 | 0.000581155 | 0.010581271 |
| GLIS2-AS1 | 0.990999709 | 0.580228299 | -0.772263973 | 0.000618361 | 0.01112305 |
| SLC9A3-AS1 | 4.191608265 | 3.221675744 | -0.379692597 | 0.000646132 | 0.011484234 |
| CEBPB-AS1 | 0.509593839 | 0.390184841 | -0.385190107 | 0.000693138 | 0.01214732 |
| AL117336.2 | 0.626552608 | 0.769103377 | 0.295741877 | 0.000707848 | 0.01214732 |
| FLG-AS1 | 0.84755455 | 0.549982406 | -0.62392076 | 0.000707848 | 0.01214732 |
| AC002116.2 | 0.503635203 | 0.655673327 | 0.380598078 | 0.000738141 | 0.012523241 |
| DUBR | 1.540574589 | 2.133680824 | 0.469875846 | 0.000775004 | 0.012881113 |
| AL445524.1 | 6.820889457 | 9.047700619 | 0.407591311 | 0.00079132 | 0.012881113 |
| AC025419.1 | 0.638427319 | 1.509290176 | 1.241275912 | 0.000796016 | 0.012881113 |
| CYTOR | 3.56161568 | 4.670980934 | 0.391193707 | 0.000802373 | 0.012881113 |
| PCAT19 | 0.486123276 | 0.317994271 | -0.612321439 | 0.000802373 | 0.012881113 |
| AC008556.1 | 1.62874201 | 1.357384728 | -0.262928415 | 0.000842199 | 0.013376631 |
| AL049838.1 | 1.184016152 | 0.884608721 | -0.420577392 | 0.000865763 | 0.013606142 |
| CACNA1C-AS2 | 0.693365623 | 0.331206118 | -1.065886986 | 0.000937273 | 0.01457655 |
| LINC01447 | 0.754170267 | 0.518669648 | -0.540074325 | 0.000984088 | 0.015146834 |
| HOTAIR | 0.6804066 | 0.437776294 | -0.636203302 | 0.001012225 | 0.015420944 |
| AF186192.1 | 0.946481688 | 0.550859921 | -0.780889093 | 0.001132298 | 0.017075967 |
| AP004609.5 | 0.546745325 | 0.373542933 | -0.549594911 | 0.001153018 | 0.017214564 |
| GNG12-AS1 | 0.468278856 | 0.642686903 | 0.456748176 | 0.001184538 | 0.017510051 |
| ZNF793-AS1 | 0.760620345 | 0.522198687 | -0.542577699 | 0.001283536 | 0.01861036 |
| AL589765.4 | 1.0147576 | 1.361067147 | 0.423603097 | 0.001283903 | 0.01861036 |
| AC037459.2 | 2.263348107 | 3.266105233 | 0.529112784 | 0.001309961 | 0.018805499 |
| AC025265.1 | 0.754898444 | 1.051432934 | 0.478002354 | 0.001368023 | 0.019451984 |
| AC079922.2 | 1.259338539 | 1.973057986 | 0.647767191 | 0.00143797 | 0.020253677 |
| AC015909.2 | 0.918961799 | 1.180913374 | 0.361826345 | 0.001506211 | 0.020960053 |
| AC020910.5 | 2.010822614 | 1.481435994 | -0.440789524 | 0.001516199 | 0.020960053 |
| AC008443.1 | 0.754915108 | 0.970076139 | 0.361783566 | 0.001556767 | 0.021323424 |
| ZNF687-AS1 | 0.730305669 | 0.919410065 | 0.33220803 | 0.001619496 | 0.021980971 |
| AL592148.3 | 0.51482568 | 0.638598336 | 0.310824773 | 0.001651725 | 0.02221644 |
| AC093278.2 | 1.097374703 | 0.729560377 | -0.588956941 | 0.001684543 | 0.022256837 |
| RAMP2-AS1 | 0.771368685 | 0.547685559 | -0.494072739 | 0.001684543 | 0.022256837 |
| AC091057.1 | 0.580956507 | 0.832415033 | 0.51887286 | 0.00171796 | 0.02249925 |
| LLPH-DT | 0.481361993 | 0.640285173 | 0.411592366 | 0.001742083 | 0.022616777 |
| AC015813.1 | 1.231975798 | 1.862230671 | 0.596057873 | 0.001763465 | 0.022697011 |
| AC114271.1 | 0.41891766 | 0.518327583 | 0.307197466 | 0.001894378 | 0.024044556 |
| LINC01293 | 2.486694912 | 4.386029553 | 0.818686016 | 0.001900374 | 0.024044556 |
| AC006547.1 | 1.269747972 | 1.601372947 | 0.33476717 | 0.002008063 | 0.025158707 |
| LINC02768 | 0.88632651 | 1.45003466 | 0.710177215 | 0.00203309 | 0.025158707 |
| AL445423.1 | 0.716522306 | 0.510000654 | -0.49051252 | 0.002055799 | 0.025158707 |
| LINC01842 | 0.732919964 | 0.512102611 | -0.517222747 | 0.002055835 | 0.025158707 |
| AC091544.6 | 3.440448978 | 1.8878988 | -0.865815417 | 0.002090839 | 0.025375504 |
| AL031055.1 | 0.700824539 | 0.541211215 | -0.372861558 | 0.002107544 | 0.025375504 |
| MED8-AS1 | 0.509851577 | 0.687399319 | 0.431071098 | 0.00212798 | 0.025377467 |
| RASSF8-AS1 | 2.00075266 | 2.519565382 | 0.332632067 | 0.002141702 | 0.025377467 |
| AL117382.2 | 0.486327407 | 0.331534872 | -0.552767271 | 0.002221467 | 0.02611536 |
| AC005288.1 | 12.4021129 | 14.47344662 | 0.222822589 | 0.002254431 | 0.026295824 |
| AC116351.1 | 0.826754462 | 0.460526703 | -0.844174115 | 0.002387621 | 0.027633478 |
| THAP9-AS1 | 5.000749949 | 6.249328717 | 0.32155676 | 0.00246487 | 0.028023028 |
| AC084262.1 | 0.38393492 | 0.552329493 | 0.524667383 | 0.002480511 | 0.028023028 |
| CCDC18-AS1 | 1.681754196 | 1.97691352 | 0.233282904 | 0.002480568 | 0.028023028 |
| AC008972.2 | 0.931782971 | 0.73644505 | -0.339416083 | 0.002496358 | 0.028023028 |
| PCBP1-AS1 | 0.638859718 | 0.79752034 | 0.320022138 | 0.002544281 | 0.028316585 |
| MAP4K3-DT | 0.896432328 | 1.073287987 | 0.259770652 | 0.002560441 | 0.028316585 |
| AC004943.2 | 0.942820115 | 1.352631323 | 0.520714224 | 0.002609487 | 0.028603523 |
| LINC00461 | 0.304414161 | 0.562362708 | 0.885465452 | 0.002624704 | 0.028603523 |
| FOXN3-AS1 | 2.420521257 | 1.978802908 | -0.290689839 | 0.002761823 | 0.029879726 |
| AC027031.2 | 3.694993607 | 5.13083264 | 0.473621099 | 0.002832101 | 0.030419612 |
| LINC01239 | 0.818826607 | 2.017768941 | 1.30113109 | 0.002936748 | 0.031222772 |
| UPK1A-AS1 | 0.592451982 | 1.051753341 | 0.828026267 | 0.002948701 | 0.031222772 |
| LINC01102 | 0.536274394 | 0.4393898 | -0.287469992 | 0.00307242 | 0.032303679 |
| AC099850.4 | 8.351066991 | 10.85516613 | 0.378349362 | 0.003110426 | 0.032446326 |
| AL365181.2 | 0.307203683 | 0.989759906 | 1.687883088 | 0.003129451 | 0.032446326 |
| PRR7-AS1 | 0.744167321 | 0.984519499 | 0.403792742 | 0.003248659 | 0.033077933 |
| SUCLG2-AS1 | 0.334645994 | 0.491873863 | 0.555652653 | 0.003248659 | 0.033077933 |
| AL360181.2 | 0.987012533 | 1.402017307 | 0.506363849 | 0.003268857 | 0.033077933 |
| HOXB-AS1 | 1.047136301 | 0.742960727 | -0.495091387 | 0.003278991 | 0.033077933 |
| AL031847.1 | 3.191247748 | 4.903954868 | 0.619825087 | 0.003309596 | 0.033162601 |
| AL450384.2 | 0.600071454 | 0.688544753 | 0.198416125 | 0.003520287 | 0.035038593 |
| AC245884.9 | 0.815286971 | 0.479310182 | -0.766348371 | 0.003584717 | 0.035443592 |
| AC099850.2 | 0.885417536 | 1.168573364 | 0.400318459 | 0.003630193 | 0.035632904 |
| AC024560.3 | 0.380716585 | 0.49043373 | 0.365340784 | 0.003651597 | 0.035632904 |
| AC018730.1 | 1.264072041 | 1.076965867 | -0.231106161 | 0.003724459 | 0.036055252 |
| AC012306.2 | 2.355067682 | 1.99102196 | -0.242259389 | 0.003743178 | 0.036055252 |
| BAALC-AS2 | 0.785365588 | 0.322064879 | -1.286013042 | 0.003879975 | 0.036901691 |
| AL606491.1 | 0.488032417 | 0.696589044 | 0.513330802 | 0.003930582 | 0.036901691 |
| AC093620.1 | 1.009190935 | 0.770805866 | -0.388759696 | 0.003954468 | 0.036901691 |
| AL365181.3 | 1.404903183 | 4.391109756 | 1.644114883 | 0.003954635 | 0.036901691 |
| AC016831.1 | 0.406631018 | 0.633152503 | 0.638832763 | 0.003954635 | 0.036901691 |
| AC105942.1 | 4.485450265 | 6.838225131 | 0.608369105 | 0.004052053 | 0.037456754 |
| GATA2-AS1 | 0.547593627 | 0.411975301 | -0.410547811 | 0.004064296 | 0.037456754 |
| AL357033.4 | 1.67468118 | 0.882900262 | -0.92356409 | 0.004126539 | 0.037797075 |
| AP001029.1 | 0.423121363 | 0.65992234 | 0.641224729 | 0.004210397 | 0.038255072 |
| DCST1-AS1 | 1.259891224 | 1.6331567 | 0.374364043 | 0.004227788 | 0.038255072 |
| AC040160.1 | 0.473392466 | 0.660204703 | 0.47987667 | 0.004279252 | 0.03848749 |
| CPB2-AS1 | 0.490692986 | 0.668179452 | 0.445414967 | 0.004305197 | 0.038488975 |
| AC006449.5 | 0.427852723 | 0.577043903 | 0.431566815 | 0.004383889 | 0.038959203 |
| AC020916.1 | 5.12421424 | 7.556884189 | 0.560460718 | 0.004410408 | 0.038962953 |
| AL162411.1 | 0.272933955 | 0.560020862 | 1.036928683 | 0.004447519 | 0.038974133 |
| DLGAP1-AS2 | 1.569529214 | 1.924825359 | 0.294395673 | 0.004463883 | 0.038974133 |
| AL122023.3 | 0.663383144 | 0.539303406 | -0.29874521 | 0.00449084 | 0.038981532 |
| LINC02289 | 0.533373243 | 0.521178317 | -0.033368388 | 0.004545197 | 0.03922531 |
| AC010894.2 | 0.891977628 | 1.137607927 | 0.350923993 | 0.004600149 | 0.039471393 |
| AC020907.1 | 6.102479013 | 4.286396164 | -0.509630233 | 0.004697669 | 0.04007783 |
| MELTF-AS1 | 2.710278019 | 3.477936307 | 0.359790662 | 0.004740172 | 0.040210662 |
| AC010319.4 | 0.417736066 | 0.510837612 | 0.290273043 | 0.004768635 | 0.040223571 |
| AC006946.3 | 0.336137912 | 0.517185498 | 0.621628551 | 0.004918323 | 0.04099684 |
| AC008555.4 | 0.771750222 | 0.307948837 | -1.325443311 | 0.004939054 | 0.04099684 |
| AC015712.6 | 0.413165201 | 0.687032157 | 0.73365888 | 0.004942687 | 0.04099684 |
| WARS2-AS1 | 1.130628642 | 1.429328144 | 0.338212018 | 0.005092097 | 0.042002764 |
| AP000525.1 | 0.700800616 | 1.07485859 | 0.617070921 | 0.005150046 | 0.042247352 |
| LINC00896 | 0.644718948 | 0.340445316 | -0.9212473 | 0.005243851 | 0.042563298 |
| VIM-AS1 | 1.057349137 | 1.268142312 | 0.262264821 | 0.005245577 | 0.042563298 |
| AC125437.1 | 0.484266406 | 0.60481978 | 0.320704398 | 0.005292419 | 0.042711252 |
| AC121761.2 | 0.395209402 | 0.527389038 | 0.416250315 | 0.00540087 | 0.043352142 |
| AL391069.2 | 2.175358172 | 1.775940032 | -0.292670093 | 0.005565138 | 0.044431822 |
| AL135999.1 | 0.570583813 | 0.695597864 | 0.285814684 | 0.00563112 | 0.044510058 |
| AL136987.1 | 0.195473219 | 0.827167493 | 2.081208529 | 0.00563523 | 0.044510058 |
| LINC00702 | 0.409965673 | 0.901183882 | 1.136318393 | 0.005664374 | 0.044510058 |
| AL157937.1 | 0.390066544 | 1.396787361 | 1.84032024 | 0.005718956 | 0.044703675 |
| AC018521.5 | 0.68848236 | 0.809945351 | 0.234404878 | 0.005799172 | 0.045094606 |
| AL031058.1 | 0.877346699 | 0.48391995 | -0.858378644 | 0.005833715 | 0.045128171 |
| AC010503.5 | 1.042713207 | 0.297153403 | -1.8110626 | 0.005927454 | 0.045616951 |
| SNHG12 | 3.669088969 | 4.334712311 | 0.240514359 | 0.006042044 | 0.045863607 |
| AC009549.1 | 2.926247789 | 4.632724573 | 0.662808974 | 0.006077478 | 0.045863607 |
| AC106897.2 | 1.401690258 | 1.974257092 | 0.494142291 | 0.006077478 | 0.045863607 |
| AP006623.1 | 0.489571071 | 0.689906949 | 0.494883481 | 0.0061131 | 0.045863607 |
| SNHG21 | 1.374229853 | 1.683205179 | 0.29258772 | 0.0061131 | 0.045863607 |
| AC068473.5 | 2.375749228 | 1.822688649 | -0.382314419 | 0.006184907 | 0.046170328 |
| LINC01579 | 0.971564651 | 0.374889139 | -1.373845973 | 0.006236221 | 0.04632178 |
| NBR2 | 2.010992809 | 2.461471989 | 0.291613399 | 0.006330803 | 0.046791532 |
| AL121985.1 | 0.776793728 | 0.378164567 | -1.038517359 | 0.006417409 | 0.046799516 |
| AC016957.2 | 0.81085514 | 0.478909987 | -0.759689677 | 0.006423542 | 0.046799516 |
| AL355803.1 | 0.477502957 | 0.348773994 | -0.453217198 | 0.006425921 | 0.046799516 |
| AL355488.1 | 1.208809554 | 1.442537718 | 0.255022074 | 0.006631924 | 0.047970344 |
| AL162431.1 | 0.492567696 | 0.610940323 | 0.310709448 | 0.006670456 | 0.047970344 |
| PANTR1 | 3.631973528 | 3.612557959 | -0.007732954 | 0.006683075 | 0.047970344 |
| AC011005.4 | 0.47608283 | 0.410739887 | -0.212987544 | 0.006749926 | 0.048218374 |
| AC135048.3 | 0.458309668 | 0.612044539 | 0.417313923 | 0.006865892 | 0.048773245 |
| AL023284.4 | 0.636279707 | 0.479881218 | -0.406983762 | 0.006892937 | 0.048773245 |
| AC115522.1 | 1.211500339 | 0.545716362 | -1.150571604 | 0.006938569 | 0.048864541 |
| AC023043.4 | 2.378139188 | 3.484399471 | 0.551076876 | 0.007066855 | 0.049534339 |
| AL732292.2 | 0.445292395 | 0.584662025 | 0.392849916 | 0.007128245 | 0.049731166 |
| LINC01357 | 0.916743326 | 0.623918521 | -0.555160221 | 0.007229825 | 0.050205248 |
| AZIN1-AS1 | 0.61553134 | 0.430172231 | -0.516917918 | 0.007484556 | 0.051733531 |
| AC104083.1 | 1.443658037 | 0.968837396 | -0.575402589 | 0.007570678 | 0.052087662 |
| AL583722.1 | 0.327395359 | 0.599602573 | 0.872972703 | 0.007670011 | 0.052495943 |
| CRIM1-DT | 1.219885388 | 0.72398134 | -0.75272119 | 0.007700343 | 0.052495943 |
| Z98884.2 | 0.858076171 | 1.048760115 | 0.2895071 | 0.007923311 | 0.053532622 |
| AC067852.2 | 2.998802125 | 3.458062086 | 0.205577442 | 0.007924119 | 0.053532622 |
| AC005281.1 | 3.527232057 | 1.08193954 | -1.704916613 | 0.007978848 | 0.053601175 |
| LINC02041 | 0.616567758 | 0.391316833 | -0.655922279 | 0.00800607 | 0.053601175 |
| AC010654.1 | 0.656489154 | 0.530469811 | -0.307500527 | 0.008060423 | 0.053724158 |
| ZNF582-AS1 | 0.755947547 | 0.621823729 | -0.281780461 | 0.008198808 | 0.054403643 |
| NINJ2-AS1 | 2.205149125 | 1.813948495 | -0.281742729 | 0.008578342 | 0.056420546 |
| AC124045.1 | 0.515440479 | 0.390862918 | -0.399143122 | 0.008578342 | 0.056420546 |
| LINC01117 | 0.894016962 | 1.22017158 | 0.448709925 | 0.008675333 | 0.056781901 |
| AC099811.3 | 0.665570357 | 0.520068473 | -0.355889597 | 0.0087246 | 0.056781901 |
| AC068580.1 | 0.911955724 | 1.221484243 | 0.421599565 | 0.008749248 | 0.056781901 |
| LINC00963 | 4.43448412 | 5.617428916 | 0.341143683 | 0.008823443 | 0.056781901 |
| AC006504.5 | 1.459643076 | 1.661326804 | 0.186720265 | 0.008823443 | 0.056781901 |
| AF131215.6 | 1.193601928 | 0.999988219 | -0.255338768 | 0.009125811 | 0.058475691 |
| LINC01446 | 0.36459776 | 0.453853069 | 0.315919612 | 0.009384888 | 0.059878792 |
| LINC00115 | 0.538348081 | 0.619096079 | 0.201624042 | 0.009490238 | 0.060037819 |
| AC018647.2 | 3.292086445 | 4.141069661 | 0.331001253 | 0.009490238 | 0.060037819 |
| AC024361.1 | 0.554598779 | 0.72365523 | 0.38385808 | 0.009677106 | 0.060706489 |
| IDH1-AS1 | 0.690247753 | 0.910016143 | 0.39877785 | 0.009677257 | 0.060706489 |
| LINC01518 | 0.50531728 | 1.020734218 | 1.014345841 | 0.009836392 | 0.061446581 |
| AP000640.1 | 0.422479511 | 0.530475275 | 0.32840413 | 0.010201541 | 0.063116989 |
| AC087752.3 | 0.813722841 | 0.607019289 | -0.422795126 | 0.010258074 | 0.063116989 |
| AC005786.2 | 0.234553672 | 0.6905621 | 1.55785307 | 0.010299189 | 0.063116989 |
| RUSC1-AS1 | 0.891595591 | 1.113320003 | 0.320406941 | 0.010315168 | 0.063116989 |
| MALINC1 | 0.460286948 | 0.529188895 | 0.201249253 | 0.010315168 | 0.063116989 |
| AC121761.1 | 0.572785704 | 0.794632169 | 0.472291714 | 0.010545662 | 0.064263972 |
| AL354696.1 | 0.525439944 | 0.667000171 | 0.344161252 | 0.010869886 | 0.065970485 |
| AL365203.2 | 1.304613806 | 2.142727654 | 0.71582569 | 0.011020437 | 0.066613411 |
| FAM230C | 0.361331303 | 0.561880735 | 0.636941691 | 0.011531591 | 0.069422035 |
| LINC01778 | 0.324442466 | 0.508001468 | 0.646869999 | 0.011765892 | 0.070279856 |
| AC004825.2 | 0.806389077 | 0.678082989 | -0.250014246 | 0.011768228 | 0.070279856 |
| STK32A-AS1 | 3.73851623 | 2.868964954 | -0.381935451 | 0.011928879 | 0.070955441 |
| ENTPD3-AS1 | 0.977504198 | 1.157661937 | 0.244039212 | 0.012092864 | 0.071645419 |
| AL139384.1 | 0.469994364 | 0.391107943 | -0.26507662 | 0.012158735 | 0.071750956 |
| SAP30-DT | 0.601559418 | 0.804317319 | 0.41905754 | 0.012224925 | 0.071857529 |
| AC124283.2 | 0.828723783 | 0.978304093 | 0.239391653 | 0.012291232 | 0.071963958 |
| AC009506.2 | 1.375927344 | 1.114054832 | -0.304584049 | 0.012358262 | 0.072073769 |
| LINC01786 | 0.660085657 | 0.806650654 | 0.289290752 | 0.012697174 | 0.073476281 |
| NALT1 | 0.660630194 | 0.462375129 | -0.514779112 | 0.012697174 | 0.073476281 |
| AC064807.1 | 1.364486561 | 1.13792164 | -0.261956972 | 0.012766034 | 0.073589535 |
| AL513477.2 | 0.433579888 | 0.522306388 | 0.268598512 | 0.012973179 | 0.074217267 |
| LINC01137 | 2.673974156 | 3.197153402 | 0.257802445 | 0.012974351 | 0.074217267 |
| AC024909.1 | 0.503976851 | 0.637279613 | 0.338569042 | 0.013044456 | 0.074333482 |
| FGD5-AS1 | 22.73448416 | 26.10757852 | 0.199586383 | 0.01318567 | 0.074688212 |
| AL353796.1 | 0.488858543 | 0.572616744 | 0.228152793 | 0.013256782 | 0.074688212 |
| AC242426.2 | 0.478597348 | 0.574607583 | 0.263764632 | 0.013256782 | 0.074688212 |
| AL357033.3 | 0.929228932 | 0.496622698 | -0.903883873 | 0.013327487 | 0.074804277 |
| AC098487.1 | 0.630553342 | 0.739829872 | 0.230575131 | 0.013544635 | 0.075274186 |
| MIR205HG | 1.977272116 | 0.161015562 | -3.618239399 | 0.013657679 | 0.075274186 |
| AL731577.2 | 0.442301621 | 0.5198695 | 0.233118988 | 0.013689402 | 0.075274186 |
| AC130371.2 | 0.799416831 | 0.642128108 | -0.316086795 | 0.013689859 | 0.075274186 |
| LINC01278 | 4.316275378 | 3.499248543 | -0.302741772 | 0.013690621 | 0.075274186 |
| AC078845.1 | 0.728789146 | 0.798097047 | 0.131062714 | 0.013729317 | 0.075274186 |
| AL390066.2 | 0.940428448 | 1.948231799 | 1.050775252 | 0.013764135 | 0.075274186 |
| AC139256.3 | 0.531043006 | 0.61729534 | 0.217132199 | 0.013912207 | 0.075806297 |
| AC096996.2 | 0.679058148 | 0.406991891 | -0.738535067 | 0.014278302 | 0.0775182 |
| TONSL-AS1 | 0.71509921 | 0.591522455 | -0.273710472 | 0.014595848 | 0.078955076 |
| AC007405.4 | 0.923051394 | 1.370523983 | 0.570244693 | 0.01466882 | 0.079063352 |
| A2M-AS1 | 1.35790748 | 1.138017888 | -0.254861952 | 0.014752012 | 0.079225734 |
| AC009120.3 | 0.400559392 | 0.541227769 | 0.434219692 | 0.015305511 | 0.081631967 |
| ELOA-AS1 | 1.15612399 | 1.305368402 | 0.175160893 | 0.015309411 | 0.081631967 |
| AC104031.1 | 0.620328315 | 0.580286322 | -0.096267057 | 0.015468797 | 0.082188308 |
| NUTM2B-AS1 | 0.514821896 | 0.58054714 | 0.173339804 | 0.015718825 | 0.083220588 |
| AC009171.2 | 0.844252352 | 0.629511487 | -0.42344159 | 0.016051889 | 0.084683641 |
| AC137630.3 | 0.353291687 | 0.531049825 | 0.587987422 | 0.016121139 | 0.084749507 |
| AC012511.1 | 1.110872221 | 1.439960434 | 0.374336292 | 0.016393933 | 0.085881201 |
| AC016394.3 | 0.86312326 | 1.228559699 | 0.509329457 | 0.01656664 | 0.086482493 |
| FAM27E3 | 0.373457869 | 0.464384117 | 0.314373134 | 0.016697191 | 0.086785508 |
| SP2-AS1 | 0.812595862 | 1.067140468 | 0.393140168 | 0.016740942 | 0.086785508 |
| CARD8-AS1 | 2.036396835 | 1.453738215 | -0.486251232 | 0.017005414 | 0.087851499 |
| AC106771.1 | 0.348725996 | 0.585519228 | 0.747622634 | 0.017172048 | 0.088406441 |
| AC021028.1 | 0.486948338 | 0.764830896 | 0.651372084 | 0.017306831 | 0.088780527 |
| SPRY4-AS1 | 0.719149395 | 0.853020368 | 0.246288685 | 0.01736364 | 0.088780527 |
| EMSLR | 2.493139219 | 1.672278433 | -0.576148371 | 0.017906075 | 0.090969662 |
| AC093627.4 | 0.71739556 | 0.556230682 | -0.367085489 | 0.017913651 | 0.090969662 |
| AL441992.2 | 2.481063571 | 3.045032881 | 0.295499105 | 0.018100314 | 0.091605995 |
| AC012360.3 | 0.676125443 | 0.793836337 | 0.231550665 | 0.018288678 | 0.092102579 |
| PICSAR | 0.961546111 | 0.080984168 | -3.569644239 | 0.018342127 | 0.092102579 |
| AC009133.1 | 0.976796854 | 1.088344241 | 0.156004492 | 0.018383502 | 0.092102579 |
| AC108488.1 | 2.197529468 | 1.864983815 | -0.236719401 | 0.018574442 | 0.092747965 |
| AC015726.1 | 1.348674603 | 1.132797795 | -0.251651946 | 0.018961534 | 0.094224129 |
| LINC01287 | 0.915104085 | 1.310868649 | 0.518515382 | 0.019030752 | 0.094224129 |
| AL157392.3 | 0.550415623 | 0.624197022 | 0.181480053 | 0.019059402 | 0.094224129 |
| AP001330.4 | 1.60105758 | 1.236775154 | -0.372441952 | 0.019157711 | 0.094397568 |
| MYLK-AS1 | 0.797903865 | 1.00493047 | 0.332808848 | 0.019355661 | 0.095059217 |
| LIPE-AS1 | 0.373143752 | 0.43264006 | 0.213435729 | 0.019455304 | 0.09523531 |
| LINC02877 | 1.956720137 | 0.127489404 | -3.93998818 | 0.019635483 | 0.095590596 |
| PRRT3-AS1 | 3.742999765 | 4.705984037 | 0.330301469 | 0.019655936 | 0.095590596 |
| SH3BP5-AS1 | 1.126803906 | 1.197000409 | 0.087187175 | 0.019756928 | 0.095769785 |
| AC106791.1 | 0.543182429 | 0.43675486 | -0.314613057 | 0.019958669 | 0.096434606 |
| NRAV | 3.705124921 | 4.204703549 | 0.182481902 | 0.020062627 | 0.096624203 |
| HOXA-AS2 | 0.480125665 | 0.474628718 | -0.016612664 | 0.020165441 | 0.096807083 |
| ERICD | 0.702755617 | 0.537361018 | -0.387131412 | 0.020476643 | 0.097859514 |
| TRPM2-AS | 1.27282768 | 1.054017481 | -0.272138321 | 0.020580719 | 0.097859514 |
| BRWD1-AS2 | 0.563481013 | 0.486167722 | -0.212912887 | 0.020581304 | 0.097859514 |
| PPP1R14B-AS1 | 1.783258265 | 2.276361672 | 0.352214133 | 0.020686432 | 0.098047118 |
| YTHDF3-AS1 | 2.037830201 | 1.667419289 | -0.289416916 | 0.020898091 | 0.098616063 |
| LINC02875 | 0.513245711 | 0.424001746 | -0.275579458 | 0.020946412 | 0.098616063 |
| AC131009.1 | 0.526032915 | 0.630411105 | 0.261139875 | 0.021004627 | 0.098616063 |
| AL513320.1 | 0.543373148 | 0.622233649 | 0.19551314 | 0.021111516 | 0.098807192 |
| AL590617.2 | 3.70664266 | 4.432683376 | 0.258067277 | 0.021219119 | 0.099000452 |
| WAC-AS1 | 6.743310942 | 7.581228339 | 0.168974495 | 0.021327079 | 0.09919417 |
| AL596244.1 | 0.983294669 | 0.856686222 | -0.198856936 | 0.021653833 | 0.10027278 |
| DLEU2 | 0.778411445 | 0.999949307 | 0.361322036 | 0.021874083 | 0.10027278 |
| AC139795.2 | 1.318281444 | 1.132222227 | -0.219501258 | 0.021874083 | 0.10027278 |
| LINC00221 | 1.143360982 | 1.988129532 | 0.798130792 | 0.021920851 | 0.10027278 |
| SPINT1-AS1 | 0.678126974 | 0.52816534 | -0.360565803 | 0.021984324 | 0.10027278 |
| AL357079.1 | 0.929381421 | 1.046218409 | 0.170841352 | 0.021984937 | 0.10027278 |
| AC072061.1 | 0.509835945 | 0.421840107 | -0.273336824 | 0.022096279 | 0.10027278 |
| TAPT1-AS1 | 0.660537012 | 0.537964205 | -0.296129219 | 0.022096279 | 0.10027278 |
| TAF1A-AS1 | 0.936873047 | 1.116063601 | 0.252493774 | 0.022208112 | 0.100341847 |
| AC079414.3 | 0.647776362 | 0.67401112 | 0.05727657 | 0.022320312 | 0.100341847 |
| AC107952.2 | 0.764932646 | 0.488872961 | -0.645873106 | 0.022430643 | 0.100341847 |
| LINC00844 | 0.766671827 | 0.172124363 | -2.155157848 | 0.022443613 | 0.100341847 |
| AC025176.1 | 0.503616976 | 0.579976233 | 0.203666868 | 0.02251101 | 0.100341847 |
| RTCA-AS1 | 1.072384258 | 1.369773805 | 0.353115729 | 0.022660378 | 0.100341847 |
| ATXN1-AS1 | 0.771151587 | 0.967513756 | 0.327267693 | 0.022660378 | 0.100341847 |
| LINC01679 | 4.51410347 | 3.904005462 | -0.209484416 | 0.022660378 | 0.100341847 |
| AC011481.1 | 0.519668727 | 0.547306902 | 0.074757808 | 0.022716373 | 0.100341847 |
| AL117335.1 | 1.253021385 | 1.12933931 | -0.149932029 | 0.022889498 | 0.100493192 |
| THUMPD3-AS1 | 1.500070983 | 1.730767753 | 0.206381377 | 0.023004811 | 0.100493192 |
| AC112721.1 | 0.300413271 | 0.764139945 | 1.346888334 | 0.023049665 | 0.100493192 |
| AP003469.2 | 0.734897994 | 0.518431312 | -0.503391156 | 0.023099038 | 0.100493192 |
| AP000759.1 | 2.740940148 | 3.146848373 | 0.19923684 | 0.023120628 | 0.100493192 |
| BX322234.2 | 0.440784803 | 4.562043432 | 3.371533792 | 0.023154493 | 0.100493192 |
| AC008267.5 | 5.470899802 | 4.919206582 | -0.153352491 | 0.023707338 | 0.102594363 |
| AL021707.2 | 0.664796488 | 0.712506018 | 0.099989439 | 0.024065529 | 0.103466031 |
| AC006538.1 | 0.930276875 | 1.233419439 | 0.406931419 | 0.024185964 | 0.103466031 |
| AC025165.5 | 1.803694389 | 1.261323677 | -0.516016372 | 0.024185964 | 0.103466031 |
| AL359504.1 | 0.968419015 | 1.081449932 | 0.159263563 | 0.024185964 | 0.103466031 |
| AC022007.1 | 1.098043474 | 1.325873246 | 0.272007684 | 0.024428403 | 0.104204589 |
| AL117336.1 | 0.683168067 | 0.876751274 | 0.359927081 | 0.024670155 | 0.10435328 |
| AL161421.1 | 1.346846679 | 1.701823513 | 0.337495802 | 0.024672946 | 0.10435328 |
| AL365330.1 | 1.482854178 | 1.760176761 | 0.247343582 | 0.024672946 | 0.10435328 |
| AC126773.4 | 4.036339979 | 2.818918587 | -0.517905885 | 0.025168061 | 0.105849067 |
| AP006621.3 | 2.10391367 | 2.536025666 | 0.269493838 | 0.025168398 | 0.105849067 |
| AC005034.4 | 3.503860572 | 3.800345701 | 0.117185293 | 0.025293599 | 0.106076807 |
| AL035461.3 | 2.727347608 | 2.970525327 | 0.123219502 | 0.025545618 | 0.106406384 |
| AC010618.3 | 0.670534958 | 0.763217769 | 0.186782211 | 0.025545618 | 0.106406384 |
| AC026367.3 | 0.449730529 | 0.59189206 | 0.396273282 | 0.025671245 | 0.106406384 |
| LINC01176 | 1.012982852 | 1.155219811 | 0.189557636 | 0.02567244 | 0.106406384 |
| AL391422.4 | 3.24968195 | 3.682612225 | 0.180430964 | 0.025799807 | 0.106406384 |
| SRP14-AS1 | 1.29113438 | 1.508475842 | 0.22445243 | 0.025799807 | 0.106406384 |
| AC027575.3 | 1.047150646 | 0.922666862 | -0.182587259 | 0.02618519 | 0.107698315 |
| AC112721.2 | 0.446481055 | 0.86062986 | 0.946793935 | 0.027732085 | 0.113601185 |
| LINC02321 | 0.836939673 | 1.076935558 | 0.363736382 | 0.02777256 | 0.113601185 |
| PSPC1-AS2 | 0.61636989 | 0.735253421 | 0.254445206 | 0.028036912 | 0.113801182 |
| AC011468.1 | 1.52088636 | 1.281235254 | -0.247376959 | 0.028050124 | 0.113801182 |
| LINC00921 | 0.532842505 | 0.415891254 | -0.357502825 | 0.028050124 | 0.113801182 |
| AC034231.1 | 1.156269878 | 1.412108806 | 0.288373087 | 0.028187609 | 0.114049052 |
| AC090825.1 | 0.927058149 | 1.227264041 | 0.404713934 | 0.028325674 | 0.114297924 |
| AC017104.1 | 0.953648534 | 1.197079978 | 0.327989977 | 0.028743372 | 0.115670767 |
| AL390755.1 | 1.584623322 | 0.487288416 | -1.70129211 | 0.029424185 | 0.118092225 |
| LINC00239 | 0.70738439 | 1.061740874 | 0.58586542 | 0.029840062 | 0.119293307 |
| AC104794.3 | 1.987188869 | 2.3664054 | 0.251968252 | 0.029883253 | 0.119293307 |
| LINC01444 | 0.474121307 | 0.65031629 | 0.455885333 | 0.03004435 | 0.119616573 |
| LINC02084 | 1.440399345 | 0.854633261 | -0.753091478 | 0.030320465 | 0.120394824 |
| POLR2J4 | 1.307932837 | 1.120380012 | -0.223300309 | 0.03061524 | 0.121242846 |
| AC025181.2 | 2.091699513 | 1.590147416 | -0.395515095 | 0.030912317 | 0.122067259 |
| RBPMS-AS1 | 1.25414088 | 0.963941294 | -0.379682227 | 0.030986933 | 0.122067259 |
| AP003400.1 | 1.0558549 | 1.765507916 | 0.741671702 | 0.031174453 | 0.12225671 |
| AC016747.1 | 2.65859135 | 3.131608258 | 0.236241715 | 0.031211866 | 0.12225671 |
| ZFPM2-AS1 | 2.073674107 | 2.417272143 | 0.221190722 | 0.031353959 | 0.12225671 |
| ZNF667-AS1 | 3.024246463 | 2.660733673 | -0.184751607 | 0.031362572 | 0.12225671 |
| SNHG16 | 12.46430225 | 14.88002248 | 0.255574582 | 0.031513902 | 0.122400607 |
| LINC01705 | 4.703589974 | 10.79091141 | 1.197982514 | 0.031563452 | 0.122400607 |
| HAGLROS | 1.883152511 | 1.335524438 | -0.495743469 | 0.031818283 | 0.123069163 |
| LINC02199 | 0.817137501 | 1.017711028 | 0.316677207 | 0.03227519 | 0.124513849 |
| LINC02315 | 0.478159583 | 0.840809993 | 0.814287624 | 0.032521031 | 0.125002775 |
| TNKS2-AS1 | 0.717921523 | 0.962064577 | 0.422307587 | 0.032747244 | 0.125002775 |
| LINC02335 | 0.558398925 | 0.634968852 | 0.185389661 | 0.032896972 | 0.125002775 |
| AC010894.3 | 0.71274584 | 0.890717884 | 0.321580847 | 0.032902865 | 0.125002775 |
| AL133520.1 | 2.17964103 | 1.962918691 | -0.15109014 | 0.03290428 | 0.125002775 |
| ATP6V0E2-AS1 | 1.705720705 | 1.459012422 | -0.225389273 | 0.03290428 | 0.125002775 |
| SBF2-AS1 | 0.973657019 | 1.1826462 | 0.280532979 | 0.033061961 | 0.125283014 |
| DLGAP1-AS1 | 3.877043892 | 3.974613095 | 0.035857361 | 0.033220288 | 0.125564277 |
| AC080112.2 | 2.597176614 | 2.335440986 | -0.153249133 | 0.033379263 | 0.125846564 |
| AL031587.1 | 0.822228246 | 0.914865106 | 0.154020104 | 0.0338533 | 0.126985953 |
| AL445222.2 | 1.041089017 | 0.842490367 | -0.305361334 | 0.033940771 | 0.126985953 |
| TMEM161B-AS1 | 1.694348028 | 2.034445122 | 0.263905122 | 0.034021689 | 0.126985953 |
| FAM111A-DT | 1.487725647 | 1.718240165 | 0.2078232 | 0.034021689 | 0.126985953 |
| TMEM44-AS1 | 2.792068883 | 3.376810907 | 0.27432686 | 0.035171377 | 0.130949791 |
| HPN-AS1 | 6.420394161 | 4.886379595 | -0.393895927 | 0.035328969 | 0.131209329 |
| AC129510.1 | 0.580094797 | 0.659056205 | 0.184112825 | 0.035505038 | 0.131536035 |
| AL162274.2 | 0.662861072 | 0.759332954 | 0.196026089 | 0.03567416 | 0.131835448 |
| AP000254.2 | 2.385172422 | 2.018761187 | -0.240623307 | 0.036012744 | 0.132464023 |
| AL355512.1 | 0.554640454 | 0.648983242 | 0.226628381 | 0.036021697 | 0.132464023 |
| C2orf27A | 0.948410563 | 1.156999428 | 0.286804515 | 0.036183061 | 0.132705668 |
| AC003092.1 | 1.144367745 | 2.160693779 | 0.916943883 | 0.036349956 | 0.132705668 |
| U62317.2 | 5.586723521 | 6.172109525 | 0.143761238 | 0.036354064 | 0.132705668 |
| AC026689.1 | 0.555315763 | 0.413088559 | -0.426857246 | 0.03661358 | 0.13330978 |
| AC007038.1 | 0.894979154 | 1.041131072 | 0.218225722 | 0.036698138 | 0.13330978 |
| AC083799.1 | 3.81459774 | 4.12415597 | 0.112567966 | 0.037219447 | 0.134875327 |
| MKLN1-AS | 1.29286036 | 1.500228495 | 0.21461579 | 0.037924322 | 0.136765733 |
| RNF144A-AS1 | 0.887912814 | 1.107862771 | 0.319289261 | 0.037924322 | 0.136765733 |
| DNAJC3-DT | 1.005973704 | 0.809841942 | -0.312880325 | 0.038102305 | 0.137076485 |
| AL133342.1 | 1.010920042 | 0.664171786 | -0.606040549 | 0.039093501 | 0.140046555 |
| AC009063.3 | 0.956251873 | 1.746097339 | 0.868671412 | 0.03926116 | 0.140046555 |
| AC022034.1 | 0.980365038 | 2.541441272 | 1.374255953 | 0.039276135 | 0.140046555 |
| AL589986.2 | 0.483943018 | 0.41124188 | -0.234849995 | 0.039303086 | 0.140046555 |
| AC015712.1 | 0.737317401 | 0.947104779 | 0.361238236 | 0.039828801 | 0.141581905 |
| AP001033.4 | 0.488877169 | 0.51718541 | 0.081209545 | 0.040200622 | 0.142564199 |
| PRKCZ-AS1 | 0.809092875 | 0.953832096 | 0.237430011 | 0.040481461 | 0.143219957 |
| Z82243.1 | 0.643653393 | 0.831261181 | 0.369017834 | 0.040669394 | 0.143544693 |
| LINC01909 | 0.985961526 | 0.752824801 | -0.389217194 | 0.040953099 | 0.143860198 |
| BX470102.1 | 0.645145485 | 0.958332006 | 0.570901016 | 0.041037756 | 0.143860198 |
| LY6E-DT | 0.847284037 | 0.606965298 | -0.481231652 | 0.041047853 | 0.143860198 |
| AC026369.2 | 0.650998493 | 0.522055726 | -0.318450391 | 0.041621275 | 0.145528253 |
| LINC01116 | 5.609561111 | 6.95775476 | 0.310733929 | 0.042007265 | 0.146527079 |
| AC087741.1 | 1.037622777 | 1.252817549 | 0.271894272 | 0.042201369 | 0.146527079 |
| AC008115.4 | 0.534578089 | 0.476194722 | -0.166849078 | 0.042201369 | 0.146527079 |
| AL359881.1 | 0.328389509 | 0.521668151 | 0.667724316 | 0.042927834 | 0.148215219 |
| AL139383.1 | 0.661688206 | 0.53252553 | -0.313300875 | 0.042974798 | 0.148215219 |
| LINC01315 | 2.519319539 | 2.08500761 | -0.272981469 | 0.042985392 | 0.148215219 |
| AC084117.1 | 0.390588522 | 0.583588165 | 0.579301074 | 0.043128263 | 0.1483652 |
| LINC00622 | 4.375291566 | 4.648769398 | 0.087469702 | 0.04378169 | 0.1499226 |
| AF131215.5 | 1.375165882 | 1.28644538 | -0.096215453 | 0.043882049 | 0.1499226 |
| AC005899.7 | 0.689874807 | 0.790151525 | 0.195794766 | 0.04396047 | 0.1499226 |
| AC005034.6 | 0.95440135 | 1.139372535 | 0.255571546 | 0.043982651 | 0.1499226 |
| AC011451.1 | 1.631719037 | 1.818362395 | 0.15624709 | 0.044387058 | 0.150956441 |
| LINC-PINT | 1.518207079 | 1.604293412 | 0.07956944 | 0.04459041 | 0.15130337 |
| AL357992.1 | 0.46748623 | 0.661990312 | 0.501886233 | 0.044774828 | 0.151584622 |
| LINC02154 | 0.205346173 | 1.772650257 | 3.109777958 | 0.044899898 | 0.151657369 |
| KDM2B-DT | 0.573184875 | 0.655701144 | 0.194037872 | 0.044999474 | 0.151657369 |
| LINC01521 | 1.065662934 | 1.379533254 | 0.372429043 | 0.045411697 | 0.152701945 |
| LINC01534 | 0.595942591 | 0.713166889 | 0.259066365 | 0.045722899 | 0.153402894 |
| UBL7-AS1 | 1.394002232 | 1.590268298 | 0.190037316 | 0.045827098 | 0.153407753 |
| AC009902.3 | 0.585690049 | 0.387643609 | -0.595406502 | 0.046985938 | 0.156935135 |
| AC069209.1 | 3.078967814 | 2.180808772 | -0.497583516 | 0.047736226 | 0.159085235 |
| AC023043.1 | 3.000091734 | 3.474203751 | 0.211675751 | 0.048169229 | 0.160170732 |
| BOLA3-AS1 | 3.199682221 | 4.042799283 | 0.337425949 | 0.048387276 | 0.16053823 |
| CAPN10-DT | 0.507391485 | 0.527082316 | 0.05492898 | 0.04882544 | 0.161632777 |
| MUC20-OT1 | 0.542067424 | 0.634927449 | 0.228119441 | 0.049488929 | 0.16346675 |
| AC006059.1 | 1.0435836 | 1.520200463 | 0.542715401 | 0.049711766 | 0.163479441 |
| STARD7-AS1 | 1.98140943 | 2.221903629 | 0.16526922 | 0.049711766 | 0.163479441 |
| AC009690.2 | 0.634890801 | 0.655196233 | 0.045418586 | 0.049935443 | 0.163721829 |
| LINC02550 | 1.435564218 | 1.127488944 | -0.348504582 | 0.050004792 | 0.163721829 |
| AL359091.3 | 0.490178177 | 0.624567937 | 0.349552252 | 0.050159964 | 0.163870517 |
| AL596202.1 | 0.526258467 | 0.829152005 | 0.655865071 | 0.050611545 | 0.164625352 |
| LINC00847 | 12.36600101 | 9.91765609 | -0.318307924 | 0.050611545 | 0.164625352 |
| EIF3J-DT | 1.789136739 | 2.044025732 | 0.192149706 | 0.050838609 | 0.164646516 |
| LINC00342 | 1.855985888 | 1.910018695 | 0.041401018 | 0.050838609 | 0.164646516 |
| AC118553.1 | 0.540783006 | 0.68329141 | 0.337451174 | 0.051524874 | 0.16581725 |
| MIR210HG | 1.446209275 | 1.757409394 | 0.281173977 | 0.051524928 | 0.16581725 |
| AC009309.2 | 1.028353227 | 1.238931343 | 0.268760343 | 0.051755418 | 0.16581725 |
| AL031985.3 | 1.770568274 | 1.468846841 | -0.269528504 | 0.051755418 | 0.16581725 |
| TMC3-AS1 | 0.759542026 | 0.867062437 | 0.191006092 | 0.051755418 | 0.16581725 |
| AC010542.6 | 1.312581964 | 1.376440578 | 0.068534815 | 0.051985684 | 0.16584668 |
| AL049840.2 | 0.89790168 | 1.116212007 | 0.313981686 | 0.051986769 | 0.16584668 |
| AC010643.1 | 0.478926094 | 0.444761692 | -0.106770513 | 0.052685965 | 0.16771886 |
| VPS9D1-AS1 | 4.437459309 | 3.774514668 | -0.233442737 | 0.052920843 | 0.168108124 |
| APTR | 2.13113887 | 2.336652265 | 0.132818446 | 0.053630567 | 0.170000925 |
| LINC00355 | 0.465795673 | 0.652225327 | 0.485673226 | 0.05409127 | 0.170789469 |
| ZNF236-DT | 1.06814885 | 0.93958974 | -0.185009842 | 0.054108117 | 0.170789469 |
| AL118506.1 | 1.693554745 | 1.579414742 | -0.100664562 | 0.05434822 | 0.171185427 |
| LINC01152 | 0.736644919 | 0.594617107 | -0.309008404 | 0.054525496 | 0.171222043 |
| SNHG18 | 3.114138003 | 2.995089197 | -0.05623391 | 0.054589211 | 0.171222043 |
| SERPINB9P1 | 1.432227879 | 0.831631442 | -0.784244846 | 0.054709481 | 0.171239528 |
| AP000873.2 | 0.478136132 | 0.559357691 | 0.226349703 | 0.055073868 | 0.172019425 |
| AL359076.1 | 2.097577502 | 1.583582636 | -0.405531963 | 0.055317538 | 0.172419801 |
| SDCBP2-AS1 | 0.915312961 | 1.089429853 | 0.251236291 | 0.055562107 | 0.172462007 |
| ACVR2B-AS1 | 1.140775973 | 1.54488691 | 0.437485731 | 0.055562107 | 0.172462007 |
| HCCAT5 | 1.099004945 | 0.641674603 | -0.776284088 | 0.055714205 | 0.172575329 |
| HAR1A | 0.482895778 | 0.377303602 | -0.355985976 | 0.056294389 | 0.174011433 |
| LINC02391 | 0.606269753 | 0.822752306 | 0.440498318 | 0.056549178 | 0.174437858 |
| GSEC | 1.331401126 | 1.552740501 | 0.221871449 | 0.057048507 | 0.175615299 |
| AC068620.1 | 0.471200493 | 0.429196989 | -0.134701095 | 0.057551265 | 0.176798434 |
| LINC00467 | 2.165677501 | 2.592006383 | 0.259250849 | 0.057804023 | 0.176847144 |
| LINC00852 | 0.670454098 | 0.549655697 | -0.286610362 | 0.057804023 | 0.176847144 |
| TMEM132D-AS1 | 1.019860186 | 3.17900633 | 1.640204504 | 0.057922808 | 0.176848164 |
| AL137003.1 | 0.68017162 | 0.790928673 | 0.217648784 | 0.058184831 | 0.177285617 |
| AC027130.1 | 8.319762151 | 3.928390866 | -1.082603803 | 0.058423299 | 0.177649664 |
| AC090907.1 | 0.955455001 | 1.021770408 | 0.096811225 | 0.058695893 | 0.178115788 |
| AC004241.3 | 0.615080106 | 0.698680489 | 0.183858539 | 0.058824298 | 0.17814336 |
| AC007292.1 | 0.575063126 | 0.621458842 | 0.111938513 | 0.059081689 | 0.178560653 |
| AC005046.2 | 0.961876765 | 1.20030556 | 0.319477743 | 0.059340016 | 0.178979077 |
| AC004812.2 | 1.621249334 | 1.482389819 | -0.129181104 | 0.05985948 | 0.180181862 |
| Z93930.2 | 0.411149579 | 0.49792723 | 0.276271564 | 0.060120623 | 0.180603803 |
| AC007406.5 | 2.689008765 | 2.253603741 | -0.254840594 | 0.06038271 | 0.18102688 |
| AC015712.4 | 0.651763005 | 0.915493542 | 0.490202244 | 0.060628714 | 0.18108819 |
| AL138724.2 | 1.337819156 | 1.504413162 | 0.169317725 | 0.060645743 | 0.18108819 |
| AL139287.1 | 4.501408576 | 4.964146778 | 0.141169254 | 0.060909726 | 0.181513414 |
| AL606469.1 | 0.378290685 | 0.532888766 | 0.494339165 | 0.061068674 | 0.181578064 |
| AC138207.5 | 2.597325602 | 1.959497972 | -0.406542803 | 0.061174659 | 0.181578064 |
| AC010969.2 | 1.569091363 | 1.697364659 | 0.113367187 | 0.061440546 | 0.182005428 |
| AL645940.1 | 0.461634778 | 0.527110018 | 0.191352194 | 0.062243713 | 0.184019531 |
| AL139002.1 | 10.21722251 | 3.175066464 | -1.686144364 | 0.062973626 | 0.185755792 |
| AC064807.4 | 0.564252071 | 0.453674603 | -0.31468191 | 0.063321336 | 0.185755792 |
| AL121906.2 | 0.427000728 | 0.570820796 | 0.418799365 | 0.063328666 | 0.185755792 |
| EMC1-AS1 | 0.935515446 | 1.129042599 | 0.271266541 | 0.063328666 | 0.185755792 |
| LINC00462 | 2.870090828 | 1.981340748 | -0.53461938 | 0.063478337 | 0.185828161 |
| NUP50-DT | 4.940801123 | 5.719582745 | 0.211164918 | 0.063602271 | 0.185828161 |
| AL121820.2 | 3.902867275 | 3.130644235 | -0.318074831 | 0.064152349 | 0.186704775 |
| PPP3CB-AS1 | 0.423387916 | 0.424911647 | 0.005182794 | 0.064152411 | 0.186704775 |
| ST3GAL5-AS1 | 1.037744286 | 1.334477637 | 0.362824142 | 0.064428704 | 0.187144076 |
| AC005746.2 | 0.467974786 | 0.397888649 | -0.234066061 | 0.064914292 | 0.188027536 |
| AC007566.1 | 0.50777022 | 0.602392742 | 0.246528602 | 0.064984735 | 0.188027536 |
| PRKAG2-AS1 | 2.482860253 | 1.814590697 | -0.452358895 | 0.065264479 | 0.188471698 |
| AC010327.6 | 0.539394009 | 0.60676013 | 0.169786791 | 0.065544967 | 0.188916285 |
| AL590666.2 | 1.788757279 | 3.321293269 | 0.89278748 | 0.065826385 | 0.188997858 |
| AP001160.1 | 0.475148606 | 0.497431554 | 0.06611923 | 0.065826448 | 0.188997858 |
| ZNF571-AS1 | 0.596575888 | 0.479839319 | -0.314154289 | 0.066962287 | 0.191522585 |
| AC027644.3 | 2.04111828 | 1.662641678 | -0.295882505 | 0.066962351 | 0.191522585 |
| AL031716.1 | 0.628879927 | 0.50564286 | -0.31466583 | 0.067387133 | 0.192369005 |
| LINC01270 | 0.452746717 | 0.649177842 | 0.519909578 | 0.067824827 | 0.192880888 |
| AC107375.1 | 1.238791331 | 0.982337865 | -0.334641977 | 0.067824827 | 0.192880888 |
| LINC00106 | 1.33576509 | 1.742645702 | 0.383612969 | 0.068404616 | 0.193883909 |
| CDK6-AS1 | 0.381317477 | 0.504965328 | 0.405191676 | 0.068437254 | 0.193883909 |
| AC022167.2 | 0.569420339 | 0.654887449 | 0.201752956 | 0.068696419 | 0.193974384 |
| CNIH3-AS2 | 2.506774786 | 2.649894354 | 0.080102456 | 0.068729035 | 0.193974384 |
| AC020765.2 | 0.634440329 | 0.484398684 | -0.389289537 | 0.068987704 | 0.194337059 |
| HLX-AS1 | 0.447500102 | 0.412350846 | -0.118015642 | 0.069209223 | 0.194376482 |
| LINC02163 | 0.884801814 | 1.178460576 | 0.413477248 | 0.069262082 | 0.194376482 |
| AC055822.1 | 0.968690078 | 0.659770353 | -0.554071213 | 0.071065485 | 0.198615263 |
| AC004540.1 | 1.37267199 | 1.141958838 | -0.265476275 | 0.071065746 | 0.198615263 |
| ELFN1-AS1 | 4.001462196 | 2.703610501 | -0.565639957 | 0.071171578 | 0.198615263 |
| ZBTB11-AS1 | 1.385807994 | 1.521279682 | 0.134558028 | 0.071366568 | 0.198787846 |
| LINC00323 | 0.728259505 | 0.542155039 | -0.425747153 | 0.071668169 | 0.199256195 |
| AL358472.3 | 0.902815 | 1.038026978 | 0.201341646 | 0.072275298 | 0.20057067 |
| HAS2-AS1 | 0.831787632 | 0.642232865 | -0.373118738 | 0.072578985 | 0.201039748 |
| AC005253.1 | 0.536495198 | 0.617694357 | 0.203327894 | 0.073193482 | 0.201992365 |
| FOXD2-AS1 | 4.020833559 | 4.979391778 | 0.308474913 | 0.073193482 | 0.201992365 |
| AC017048.3 | 0.39174616 | 0.548476162 | 0.485509786 | 0.07346128 | 0.202357362 |
| AL157871.2 | 0.937954745 | 1.269213993 | 0.43634511 | 0.073809556 | 0.202572903 |
| AC023157.2 | 1.325560518 | 1.139305172 | -0.218448301 | 0.07381089 | 0.202572903 |
| AC005291.2 | 2.65294582 | 2.393251615 | -0.14862313 | 0.074599586 | 0.204361801 |
| LINC01963 | 3.075677291 | 2.602889077 | -0.240790307 | 0.07474498 | 0.204385082 |
| ERVK-28 | 0.398605153 | 0.56081897 | 0.492574789 | 0.074946555 | 0.204561621 |
| PXN-AS1 | 1.256463754 | 1.405939141 | 0.162165091 | 0.075373054 | 0.205350309 |
| AC005632.3 | 1.553118414 | 1.200758861 | -0.371221374 | 0.07600543 | 0.206320193 |
| AC068338.2 | 1.225410233 | 1.09061265 | -0.168126009 | 0.07600543 | 0.206320193 |
| AC010967.1 | 4.697103309 | 5.716183879 | 0.283281001 | 0.076218578 | 0.206432233 |
| AC125257.1 | 4.127078178 | 4.406925882 | 0.094651866 | 0.076323237 | 0.206432233 |
| AL133338.1 | 0.50700609 | 0.614122828 | 0.276524157 | 0.076642127 | 0.206546382 |
| HCG11 | 4.627778877 | 3.893890664 | -0.249107556 | 0.076642127 | 0.206546382 |
| AC016705.2 | 1.280190717 | 1.594767113 | 0.316987007 | 0.076962034 | 0.206662625 |
| AC009812.1 | 1.650472294 | 1.181810105 | -0.481880681 | 0.076962103 | 0.206662625 |
| LINC00589 | 2.559395821 | 4.556367187 | 0.832080732 | 0.077281798 | 0.207148517 |
| AC007114.1 | 1.738191059 | 2.038839773 | 0.230161733 | 0.07760532 | 0.207642909 |
| AL391121.1 | 0.732215055 | 0.884770792 | 0.273036323 | 0.077928567 | 0.208134796 |
| LINC01502 | 0.7838808 | 4.752125101 | 2.599866622 | 0.078808685 | 0.210109582 |
| AC096586.2 | 0.447739169 | 0.504847221 | 0.173188325 | 0.079200967 | 0.210779045 |
| AP000442.1 | 0.527535728 | 0.620302433 | 0.233702977 | 0.079561286 | 0.210985789 |
| DOCK9-DT | 0.950389034 | 0.834808623 | -0.187072688 | 0.079561286 | 0.210985789 |
| AC069544.1 | 0.622181239 | 0.697087718 | 0.164005314 | 0.080222112 | 0.212361016 |
| SNHG7 | 12.32781364 | 9.019622103 | -0.450778063 | 0.080554193 | 0.212862673 |
| AC009159.3 | 0.761565836 | 0.710453766 | -0.100227994 | 0.081221635 | 0.2138695 |
| LINC01355 | 0.849811144 | 0.904334385 | 0.089714058 | 0.081221706 | 0.2138695 |
| LINC01198 | 1.4179748 | 2.376750412 | 0.745158517 | 0.081551221 | 0.213887558 |
| AC127496.5 | 0.679699658 | 0.60586967 | -0.165889912 | 0.081557141 | 0.213887558 |
| AC090772.3 | 0.674230932 | 0.561450148 | -0.264084887 | 0.0818937 | 0.213887558 |
| AL031432.3 | 0.414258016 | 0.437267166 | 0.07798541 | 0.0818937 | 0.213887558 |
| LINC00944 | 0.695644458 | 1.098002781 | 0.658459665 | 0.082191736 | 0.213887558 |
| AC074117.1 | 1.477218673 | 1.712029405 | 0.212824076 | 0.082231385 | 0.213887558 |
| AL139393.3 | 0.814485436 | 0.484246494 | -0.750147298 | 0.082231385 | 0.213887558 |
| ZNRD2-AS1 | 0.819642851 | 0.732440433 | -0.162283975 | 0.082910142 | 0.214904239 |
| AC012313.5 | 0.530412162 | 0.444537563 | -0.254808526 | 0.082910142 | 0.214904239 |
| PRKCQ-AS1 | 0.626506835 | 0.36385721 | -0.78395785 | 0.083251219 | 0.215414332 |
| LINC00479 | 0.731230904 | 0.339884223 | -1.10528365 | 0.08422519 | 0.217557455 |
| AP003392.1 | 0.581697467 | 0.687613586 | 0.241329028 | 0.084967637 | 0.219096168 |
| AP005329.2 | 0.504804629 | 0.536722073 | 0.08845008 | 0.0856707 | 0.220528197 |
| AC104667.2 | 0.873626341 | 1.145107695 | 0.390395026 | 0.086372032 | 0.221570236 |
| AC009237.14 | 3.41401357 | 3.867445157 | 0.17991204 | 0.086372322 | 0.221570236 |
| HYI-AS1 | 0.425028567 | 0.511010495 | 0.26579311 | 0.08690085 | 0.222543686 |
| AC048344.4 | 0.708934184 | 0.763979175 | 0.107881617 | 0.087253311 | 0.223063687 |
| AC012368.1 | 1.054607854 | 0.752435237 | -0.487067329 | 0.087433451 | 0.223142124 |
| AC005019.2 | 0.925009525 | 1.257614184 | 0.443149268 | 0.089586134 | 0.228006559 |
| AC091965.1 | 0.351912074 | 0.454845621 | 0.370161951 | 0.08976242 | 0.228006559 |
| AC008764.6 | 0.45657446 | 0.493216038 | 0.111369554 | 0.089950344 | 0.228006559 |
| CD27-AS1 | 4.323194961 | 4.933170085 | 0.190417131 | 0.089950344 | 0.228006559 |
| AC112484.1 | 0.384630492 | 0.429160255 | 0.158043334 | 0.09068007 | 0.229466686 |
| AC109460.2 | 0.509545635 | 0.595897702 | 0.225853323 | 0.091230409 | 0.230155016 |
| LINC01352 | 0.742963449 | 0.611830705 | -0.280158726 | 0.091408259 | 0.230155016 |
| AP002387.2 | 2.70863336 | 2.909265315 | 0.103089747 | 0.091414551 | 0.230155016 |
| Z97205.2 | 0.689882879 | 0.868902304 | 0.332842518 | 0.091780674 | 0.230469979 |
| AL355990.2 | 0.847002273 | 0.988196602 | 0.222432254 | 0.091848384 | 0.230469979 |
| AC015909.3 | 1.492898805 | 1.849812447 | 0.309262626 | 0.092153505 | 0.230847623 |
| MSC-AS1 | 2.265086719 | 1.938713606 | -0.224466586 | 0.092897856 | 0.232322443 |
| LINC02104 | 0.94072774 | 0.894483141 | -0.072722957 | 0.094150883 | 0.235062322 |
| AC131009.3 | 1.445653628 | 1.64419782 | 0.185661955 | 0.094400418 | 0.235291861 |
| AL136162.1 | 0.550856267 | 0.629830317 | 0.193287273 | 0.094779087 | 0.235449546 |
| UBA6-AS1 | 2.998715443 | 2.424782916 | -0.306489035 | 0.094779087 | 0.235449546 |
| AC123777.1 | 2.878278716 | 4.844429915 | 0.751120601 | 0.095106965 | 0.235609195 |
| NCK1-DT | 1.479817064 | 1.675607423 | 0.17926534 | 0.095158972 | 0.235609195 |
| ARHGAP31-AS1 | 0.534583663 | 0.724849229 | 0.43926519 | 0.095539769 | 0.235770797 |
| AC021087.1 | 0.972889293 | 1.048207646 | 0.107576985 | 0.095540075 | 0.235770797 |
| AC005104.1 | 0.567903511 | 0.649514045 | 0.19371489 | 0.095879395 | 0.236217717 |
| GAS6-AS1 | 1.218423668 | 0.829017559 | -0.555541307 | 0.096305948 | 0.236877727 |
| AC012313.1 | 1.990417014 | 2.221598813 | 0.158527588 | 0.096690722 | 0.237432974 |
| AC004908.2 | 0.658161827 | 0.73242551 | 0.154239687 | 0.097076416 | 0.237988653 |
| OIP5-AS1 | 8.885208322 | 10.70507474 | 0.268817362 | 0.098242131 | 0.240451642 |
| AC022098.1 | 0.913010787 | 1.030769017 | 0.175017267 | 0.098633074 | 0.241013387 |
| AC087741.2 | 1.981873788 | 2.234690401 | 0.173209881 | 0.099418685 | 0.242140451 |
| AC010973.2 | 0.867797693 | 0.979022902 | 0.173983857 | 0.099418685 | 0.242140451 |
| AC100801.1 | 0.513396143 | 0.296808987 | -0.790537679 | 0.100378577 | 0.244080155 |
| AC078820.2 | 0.427117437 | 0.668927119 | 0.647216235 | 0.100835366 | 0.244792196 |
| LINC00987 | 1.561832861 | 1.16257344 | -0.425918218 | 0.10100488 | 0.244805659 |
| AF279873.3 | 0.735004604 | 1.094702261 | 0.574713345 | 0.101198726 | 0.244877955 |
| AC091932.1 | 1.15088011 | 1.444343052 | 0.327675891 | 0.101814338 | 0.245968944 |
| AL359643.3 | 2.098163255 | 1.947447938 | -0.107542176 | 0.102207707 | 0.246520365 |
| AL122035.2 | 0.482943813 | 0.543477384 | 0.170364648 | 0.102611172 | 0.247094323 |
| AL137779.2 | 0.733439978 | 0.655709062 | -0.161623072 | 0.103015586 | 0.247271294 |
| AC027097.2 | 1.288656249 | 1.509243192 | 0.22795782 | 0.103015904 | 0.247271294 |
| AC114810.1 | 0.791125258 | 0.670345037 | -0.239002268 | 0.104442391 | 0.25029292 |
| LINC01184 | 2.751708838 | 3.029800833 | 0.138895135 | 0.104647541 | 0.250382657 |
| AL683813.2 | 0.391005887 | 0.483431326 | 0.306120632 | 0.10485285 | 0.250472487 |
| AC006449.2 | 0.692318497 | 0.78724517 | 0.185377108 | 0.105058639 | 0.250563176 |
| MAPKAPK5-AS1 | 5.184371223 | 5.499902023 | 0.085236893 | 0.105884682 | 0.251116057 |
| PRKAR2A-AS1 | 0.380700334 | 0.420331534 | 0.14287186 | 0.105884682 | 0.251116057 |
| AC010719.1 | 0.921715111 | 0.787207424 | -0.227577077 | 0.106289929 | 0.251116057 |
| AL050341.2 | 3.428221763 | 3.634539492 | 0.084312145 | 0.10629963 | 0.251116057 |
| LINC02846 | 0.464583121 | 0.400317143 | -0.214793341 | 0.10629963 | 0.251116057 |
| ARRDC1-AS1 | 3.928017904 | 4.348916031 | 0.146854348 | 0.10629963 | 0.251116057 |
| AC009120.2 | 0.820157098 | 0.860695845 | 0.069603224 | 0.106715867 | 0.25170109 |
| AL162231.2 | 1.223471205 | 1.430160546 | 0.225196962 | 0.107552215 | 0.252477134 |
| TMEM147-AS1 | 1.684649393 | 1.956643609 | 0.21593263 | 0.107552215 | 0.252477134 |
| AL117332.1 | 1.27896323 | 1.181844203 | -0.113934922 | 0.107552215 | 0.252477134 |
| AL162724.2 | 0.692374016 | 0.605129801 | -0.194306948 | 0.107755275 | 0.252556712 |
| AC087501.4 | 0.697333875 | 0.590648228 | -0.239550404 | 0.107972332 | 0.252668795 |
| AL078644.2 | 0.483708318 | 0.553827749 | 0.195299993 | 0.108816461 | 0.253943899 |
| LINC01697 | 0.660589284 | 0.217061369 | -1.605650576 | 0.108950002 | 0.253943899 |
| AC139100.2 | 0.647798902 | 0.743953576 | 0.199666575 | 0.109027488 | 0.253943899 |
| MIR600HG | 0.819455366 | 0.626693982 | -0.386904232 | 0.110092432 | 0.256024924 |
| FGF14-AS2 | 1.756346269 | 1.559662472 | -0.171343457 | 0.110520291 | 0.256620209 |
| PLS3-AS1 | 0.391083552 | 0.427612421 | 0.128826896 | 0.110734754 | 0.256718924 |
| AC068888.1 | 1.274173246 | 1.139072255 | -0.161702185 | 0.111380197 | 0.257415841 |
| AL359921.2 | 2.295184771 | 2.380127689 | 0.052428673 | 0.111380197 | 0.257415841 |
| AL604028.1 | 2.71517499 | 3.040024259 | 0.163037655 | 0.11224529 | 0.259014247 |
| AC132872.3 | 0.818594418 | 0.978942733 | 0.258075637 | 0.112462306 | 0.259114542 |
| ZNF32-AS2 | 0.428657965 | 0.503341101 | 0.231709455 | 0.113333264 | 0.260718896 |
| AL161785.1 | 0.588777554 | 0.524627211 | -0.166430036 | 0.113552521 | 0.260821406 |
| AL139289.1 | 0.687709151 | 0.861999338 | 0.325888219 | 0.114872388 | 0.262847558 |
| AC124312.2 | 0.425365979 | 0.57003457 | 0.422344765 | 0.114988724 | 0.262847558 |
| AL049870.3 | 1.056260324 | 0.685991517 | -0.622702802 | 0.115229163 | 0.262847558 |
| AC135050.5 | 0.509779957 | 0.617355848 | 0.276227656 | 0.115313222 | 0.262847558 |
| RRN3P2 | 0.510610297 | 0.36413089 | -0.4877655 | 0.115314903 | 0.262847558 |
| AC018926.2 | 0.474678372 | 0.47753735 | 0.008663261 | 0.11619383 | 0.264067737 |
| AL513534.3 | 0.896150017 | 0.742490964 | -0.271366794 | 0.116203954 | 0.264067737 |
| AL355802.3 | 0.674849658 | 0.711078713 | 0.075443132 | 0.117098382 | 0.265695873 |
| AC025287.3 | 0.411242451 | 0.490338678 | 0.253789369 | 0.120935557 | 0.27398602 |
| AC005332.5 | 1.314509233 | 1.515001334 | 0.204794788 | 0.121190366 | 0.274147298 |
| NFYC-AS1 | 0.593497053 | 0.620270654 | 0.063657004 | 0.121651852 | 0.27477491 |
| AC090673.1 | 0.458322443 | 0.658860943 | 0.523611074 | 0.122361395 | 0.275960065 |
| AL049840.7 | 0.456890582 | 0.553450744 | 0.276606222 | 0.122577217 | 0.276029841 |
| AC009495.3 | 0.774779174 | 0.622572094 | -0.315544262 | 0.123497235 | 0.277514287 |
| AC112487.1 | 0.978243483 | 1.22815712 | 0.328229639 | 0.123803996 | 0.277514287 |
| CD44-AS1 | 0.573010114 | 0.633221099 | 0.144148725 | 0.123972626 | 0.277514287 |
| B4GALT1-AS1 | 0.682408255 | 0.605781376 | -0.171837872 | 0.123979926 | 0.277514287 |
| AC048341.2 | 2.28109712 | 2.507923312 | 0.136765362 | 0.124449337 | 0.277914742 |
| AL049555.1 | 0.875268153 | 0.227548726 | -1.943549567 | 0.12453112 | 0.277914742 |
| AC127024.4 | 0.582699565 | 0.647754521 | 0.152694946 | 0.124889089 | 0.278297627 |
| AC092652.1 | 0.57353198 | 0.249101181 | -1.203142074 | 0.125178111 | 0.278525962 |
| AL031673.1 | 0.70823351 | 0.838531158 | 0.243639286 | 0.125390312 | 0.27858294 |
| AL133467.1 | 0.533837532 | 0.306692879 | -0.799606067 | 0.125842272 | 0.278812872 |
| PINK1-AS | 1.237193351 | 1.455964425 | 0.23490412 | 0.125867298 | 0.278812872 |
| LINC00839 | 3.612515789 | 4.920275052 | 0.445735074 | 0.126342621 | 0.279451159 |
| SNHG1 | 11.08518746 | 12.27722457 | 0.147351289 | 0.12681934 | 0.280090643 |
| POLH-AS1 | 0.913184139 | 1.195218435 | 0.388296598 | 0.127297458 | 0.280731322 |
| AC016065.1 | 1.574307683 | 1.742665177 | 0.146577878 | 0.127776978 | 0.281373198 |
| AC109347.1 | 0.481029224 | 0.564387183 | 0.230560679 | 0.12874023 | 0.283076825 |
| AL662797.2 | 0.550557363 | 0.621687831 | 0.175297453 | 0.129709116 | 0.284600506 |
| AL513218.1 | 0.416977007 | 0.446713255 | 0.099381233 | 0.129937577 | 0.284600506 |
| AC090114.2 | 1.562330442 | 1.538830894 | -0.021864925 | 0.130195677 | 0.284600506 |
| ASB16-AS1 | 3.52140846 | 3.805313364 | 0.111862683 | 0.130195677 | 0.284600506 |
| AC011731.1 | 0.420011325 | 0.462544928 | 0.139165276 | 0.13068054 | 0.284610984 |
| AC007541.1 | 1.007351558 | 1.089385309 | 0.112947056 | 0.130683653 | 0.284610984 |
| AC079949.1 | 1.221698716 | 1.660601811 | 0.442817632 | 0.130772361 | 0.284610984 |
| AC078880.5 | 0.928823984 | 1.365269736 | 0.555708882 | 0.131615796 | 0.28602967 |
| AC083964.1 | 0.497788357 | 0.386169294 | -0.366299032 | 0.132646623 | 0.287756476 |
| AC103591.3 | 0.676979017 | 0.731173144 | 0.111101962 | 0.132902982 | 0.287756476 |
| UBR5-AS1 | 2.044282126 | 1.554218248 | -0.395405207 | 0.133144844 | 0.287756476 |
| AL136298.1 | 0.460460833 | 0.640907921 | 0.477038655 | 0.133379748 | 0.287756476 |
| AL133330.1 | 0.465004395 | 0.575235471 | 0.306908288 | 0.133385384 | 0.287756476 |
| AC004908.1 | 0.632604523 | 0.692803618 | 0.131142592 | 0.134363067 | 0.287756476 |
| PCAT7 | 1.329832658 | 1.05950456 | -0.327854915 | 0.134377521 | 0.287756476 |
| AL359921.1 | 0.52220329 | 0.528972987 | 0.018582503 | 0.134638329 | 0.287756476 |
| RPARP-AS1 | 0.997919323 | 1.019061196 | 0.0302456 | 0.13463869 | 0.287756476 |
| AL365434.2 | 1.689635938 | 1.352121214 | -0.321487935 | 0.134729173 | 0.287756476 |
| LINC00997 | 2.047441348 | 2.368456065 | 0.210124786 | 0.135139507 | 0.287756476 |
| AL844908.1 | 0.646710116 | 0.413743474 | -0.644382622 | 0.135390364 | 0.287756476 |
| AC093484.4 | 0.935430381 | 1.145708999 | 0.292538468 | 0.135640858 | 0.287756476 |
| AC069307.1 | 0.613652338 | 0.484274544 | -0.341596366 | 0.135641401 | 0.287756476 |
| AC011468.5 | 0.531820747 | 0.467306506 | -0.186570937 | 0.135641762 | 0.287756476 |
| HLA-F-AS1 | 1.355731007 | 1.779964114 | 0.392777196 | 0.135641762 | 0.287756476 |
| AC022509.3 | 1.297810017 | 1.597041537 | 0.299322629 | 0.13589334 | 0.287756476 |
| AC090517.2 | 0.475535491 | 0.505404471 | 0.087885407 | 0.136144553 | 0.287756476 |
| AL592435.1 | 0.378219028 | 0.432849251 | 0.194642715 | 0.136145458 | 0.287756476 |
| AC010504.1 | 0.45812878 | 0.418322894 | -0.13113624 | 0.136650234 | 0.287756476 |
| AC156455.1 | 1.297464598 | 1.592042847 | 0.29518399 | 0.136650597 | 0.287756476 |
| LINC02610 | 0.863144387 | 1.12813849 | 0.386270364 | 0.136650597 | 0.287756476 |
| AC012442.3 | 0.425587725 | 0.434093536 | 0.028549401 | 0.137154002 | 0.288409753 |
| AC002091.1 | 0.542613936 | 0.373211821 | -0.539931421 | 0.137654295 | 0.28905466 |
| AC087500.1 | 0.468738437 | 0.403639507 | -0.215715712 | 0.138174692 | 0.289333543 |
| AL162258.2 | 0.400159952 | 0.43730898 | 0.128076185 | 0.138174692 | 0.289333543 |
| ARHGAP15-AS1 | 1.944755139 | 0.879801647 | -1.144338312 | 0.138651976 | 0.289926331 |
| AC021188.1 | 0.678192368 | 0.788816803 | 0.217995735 | 0.139454659 | 0.291196931 |
| SNHG19 | 30.48722963 | 22.93601255 | -0.41059046 | 0.139711854 | 0.291326534 |
| LINC00654 | 0.587029669 | 0.457504524 | -0.359647411 | 0.140227155 | 0.291993226 |
| MIR3142HG | 13.20508457 | 16.48907826 | 0.320417214 | 0.140743917 | 0.292661098 |
| AC046143.2 | 1.029291192 | 1.174710553 | 0.190654137 | 0.141262051 | 0.292922748 |
| AC144831.1 | 0.485947305 | 0.431195104 | -0.172459082 | 0.141262142 | 0.292922748 |
| AC107068.1 | 0.518430487 | 0.559356186 | 0.109616691 | 0.142302991 | 0.294671796 |
| AC090181.2 | 0.806486512 | 0.818955198 | 0.022134124 | 0.144930776 | 0.299697574 |
| AC008735.2 | 2.544191185 | 2.782280528 | 0.129060802 | 0.146525347 | 0.302575854 |
| Z98200.2 | 0.737093178 | 0.822962856 | 0.158980311 | 0.147594828 | 0.303945485 |
| UBE2D3-AS1 | 1.57471182 | 1.72306346 | 0.129888004 | 0.147595765 | 0.303945485 |
| MACORIS | 4.68394614 | 7.040900568 | 0.588035482 | 0.148399209 | 0.30517909 |
| AL049840.4 | 1.993383 | 2.211036751 | 0.149504076 | 0.148672149 | 0.305319832 |
| AC027307.3 | 0.714898169 | 0.624794328 | -0.1943564 | 0.149212584 | 0.306008774 |
| AL022328.2 | 1.499570868 | 1.558113439 | 0.05525057 | 0.149754517 | 0.306698894 |
| AL645933.3 | 1.380934247 | 1.586063158 | 0.199805593 | 0.15029795 | 0.307390191 |
| AC008074.2 | 0.622363855 | 0.372645709 | -0.739953629 | 0.151389326 | 0.309198719 |
| AC138956.2 | 0.424447525 | 0.485197474 | 0.192985836 | 0.151655163 | 0.309318522 |
| MAP3K2-DT | 0.480973811 | 0.590554753 | 0.296112484 | 0.153037695 | 0.311712522 |
| AL357146.1 | 0.817550079 | 1.122624976 | 0.457497051 | 0.153536923 | 0.311986572 |
| MAFG-DT | 1.967894448 | 2.355220433 | 0.259209252 | 0.153590175 | 0.311986572 |
| AL049597.2 | 0.684817185 | 0.868116979 | 0.342170552 | 0.154144169 | 0.312686474 |
| NKILA | 0.416669386 | 0.852804027 | 1.033311145 | 0.154699681 | 0.31338755 |
| AC092953.2 | 0.579953478 | 0.66024079 | 0.187055095 | 0.155532455 | 0.314228424 |
| AC009005.1 | 1.099506785 | 1.314380084 | 0.257526018 | 0.155535703 | 0.314228424 |
| LMNTD2-AS1 | 0.40352747 | 0.520339716 | 0.366786937 | 0.156374959 | 0.315211274 |
| AC006213.4 | 0.658123678 | 0.583903953 | -0.17262765 | 0.156936561 | 0.315211274 |
| AC021016.1 | 0.995514577 | 0.85666338 | -0.216714023 | 0.156936944 | 0.315211274 |
| AC092910.3 | 0.382718559 | 0.443124361 | 0.211427781 | 0.156936944 | 0.315211274 |
| AC023469.1 | 0.310764071 | 0.908950527 | 1.548382055 | 0.157155413 | 0.315211274 |
| NNT-AS1 | 3.825090434 | 4.387152911 | 0.197791135 | 0.157500074 | 0.315211274 |
| SENCR | 0.486302264 | 0.41920843 | -0.214185579 | 0.157500074 | 0.315211274 |
| ATP1A1-AS1 | 0.944805774 | 1.024251006 | 0.116479623 | 0.158064733 | 0.315383137 |
| AC060766.4 | 1.600326222 | 1.484183989 | -0.108696076 | 0.158064733 | 0.315383137 |
| AC078923.1 | 0.413213677 | 0.640013564 | 0.631214473 | 0.158219671 | 0.315383137 |
| AC009902.2 | 2.152498854 | 1.494118534 | -0.526717862 | 0.159198265 | 0.31648946 |
| TNRC6C-AS1 | 2.773972537 | 2.129918632 | -0.381155188 | 0.15919865 | 0.31648946 |
| LINC00957 | 2.111960766 | 2.504921973 | 0.246182631 | 0.161484932 | 0.320607718 |
| AC093297.2 | 2.909959338 | 3.228524439 | 0.149875954 | 0.162060358 | 0.321322861 |
| Z99572.1 | 1.348055362 | 1.453108889 | 0.108263069 | 0.16321585 | 0.323184701 |
| AC011374.2 | 1.025735878 | 1.180798067 | 0.203102973 | 0.164377543 | 0.325053871 |
| MZF1-AS1 | 0.57627623 | 0.67137204 | 0.220351944 | 0.16496072 | 0.325375632 |
| SNHG17 | 7.357307606 | 8.356027056 | 0.183639253 | 0.165545454 | 0.325375632 |
| AP000593.3 | 1.670386357 | 1.678461682 | 0.006957768 | 0.165773797 | 0.325375632 |
| LINC02802 | 0.500178818 | 0.747554938 | 0.579735646 | 0.166131647 | 0.325375632 |
| AP002748.4 | 1.114681912 | 1.152412741 | 0.048025439 | 0.166131745 | 0.325375632 |
| ITGA9-AS1 | 0.563745971 | 0.620084459 | 0.137419515 | 0.166131745 | 0.325375632 |
| LINC02361 | 0.85894885 | 0.620452928 | -0.469250462 | 0.166425378 | 0.325375632 |
| AL031651.2 | 3.265512166 | 2.51006903 | -0.379582243 | 0.166714107 | 0.325375632 |
| AC136475.1 | 0.504591228 | 0.393659411 | -0.358167158 | 0.166719597 | 0.325375632 |
| AC006333.1 | 3.0579041 | 2.753421716 | -0.151317572 | 0.166719597 | 0.325375632 |
| AL450326.1 | 1.505591163 | 1.781323809 | 0.242619728 | 0.167309011 | 0.326099678 |
| AL353699.1 | 0.56732753 | 0.825623299 | 0.541301811 | 0.168567302 | 0.328123835 |
| AL136084.3 | 0.744820403 | 0.619042176 | -0.266854889 | 0.170872873 | 0.332178645 |
| AC020658.5 | 0.437146642 | 0.426744181 | -0.034745835 | 0.171551656 | 0.333064528 |
| AC004076.2 | 0.451261017 | 0.359076373 | -0.329671428 | 0.172080798 | 0.333225203 |
| AC073896.4 | 7.508372159 | 6.805044219 | -0.141895624 | 0.172080798 | 0.333225203 |
| AL158166.1 | 0.240306441 | 0.679302487 | 1.499178817 | 0.172666278 | 0.333925847 |
| SCAT2 | 0.480510649 | 0.505704783 | 0.07372702 | 0.174503548 | 0.337042428 |
| CASC9 | 0.509638624 | 0.410646494 | -0.31157764 | 0.176681068 | 0.34080728 |
| AC011476.3 | 0.548375855 | 0.526182367 | -0.059602147 | 0.176953688 | 0.340892718 |
| AL355075.2 | 0.475494963 | 0.549712849 | 0.209248143 | 0.178131636 | 0.341946553 |
| AC133552.5 | 2.514858362 | 2.785487169 | 0.147452521 | 0.178187822 | 0.341946553 |
| AC104825.1 | 0.765424261 | 0.671389975 | -0.189108634 | 0.178187822 | 0.341946553 |
| AC006213.5 | 0.431020092 | 0.398582881 | -0.112875375 | 0.178451677 | 0.342013291 |
| AC233728.1 | 0.526118381 | 0.580870496 | 0.142829101 | 0.180443406 | 0.344946554 |
| LAMC1-AS1 | 0.497737119 | 0.549845348 | 0.143641915 | 0.180618844 | 0.344946554 |
| LINC00346 | 0.883502909 | 1.192197826 | 0.432316858 | 0.180675288 | 0.344946554 |
| LINC02560 | 1.991848887 | 0.177076239 | -3.491665656 | 0.180971779 | 0.345071348 |
| AC132192.2 | 0.817454953 | 0.83014337 | 0.022221288 | 0.18192865 | 0.346453411 |
| AC015802.3 | 0.830727787 | 0.677118972 | -0.29496647 | 0.183188349 | 0.346958797 |
| SNHG11 | 3.956222748 | 4.370803082 | 0.143774725 | 0.18318845 | 0.346958797 |
| AC012063.1 | 0.386317254 | 0.443628665 | 0.199566475 | 0.18318845 | 0.346958797 |
| AC015849.3 | 0.948440951 | 1.06832357 | 0.171718811 | 0.183817222 | 0.346958797 |
| TMEM9B-AS1 | 1.358950221 | 1.475932618 | 0.119134248 | 0.183820769 | 0.346958797 |
| AL354872.1 | 0.574613119 | 0.698914364 | 0.282524765 | 0.183820769 | 0.346958797 |
| LINC01569 | 1.155208786 | 0.990894903 | -0.221349666 | 0.183820769 | 0.346958797 |
| AC012360.2 | 0.799575346 | 0.726266099 | -0.13873575 | 0.184454297 | 0.347714982 |
| LINC01711 | 1.514306485 | 2.337931517 | 0.626575444 | 0.18536239 | 0.348355581 |
| AC108047.1 | 0.564762046 | 0.493977005 | -0.193199253 | 0.185725389 | 0.348355581 |
| AL662791.1 | 1.000017648 | 0.874335924 | -0.19376588 | 0.185726406 | 0.348355581 |
| AC073896.2 | 1.172657144 | 1.24014323 | 0.080725488 | 0.185727423 | 0.348355581 |
| AC006001.2 | 1.675725975 | 1.881046003 | 0.166748881 | 0.187006624 | 0.34943791 |
| RNASEH1-AS1 | 4.898181625 | 5.439738608 | 0.151291058 | 0.187006624 | 0.34943791 |
| AC025857.2 | 7.124687951 | 8.954770523 | 0.329829634 | 0.187006624 | 0.34943791 |
| AC063948.1 | 0.677165649 | 0.696610336 | 0.04084309 | 0.188292321 | 0.351400545 |
| AC245060.2 | 0.475030073 | 0.556475361 | 0.22829896 | 0.188900394 | 0.352095242 |
| AC020907.4 | 0.553433139 | 0.431765446 | -0.358161244 | 0.190232666 | 0.354136372 |
| AC093799.1 | 0.409359032 | 0.446176483 | 0.124247748 | 0.191857998 | 0.356440243 |
| PHKA2-AS1 | 0.691508231 | 0.432072013 | -0.678474639 | 0.192184926 | 0.356440243 |
| AL139286.1 | 0.438300573 | 0.493752034 | 0.171866127 | 0.192186467 | 0.356440243 |
| AC005670.3 | 1.351294196 | 1.537924595 | 0.186642965 | 0.192843611 | 0.356661737 |
| MIR4458HG | 1.491531142 | 1.62746781 | 0.125834908 | 0.192843611 | 0.356661737 |
| MAFA-AS1 | 0.328508765 | 0.522044734 | 0.668241574 | 0.193060245 | 0.356661737 |
| AC068792.1 | 0.487650097 | 0.557514723 | 0.193163561 | 0.193499927 | 0.356661737 |
| AC124016.3 | 1.120597445 | 0.898777777 | -0.318231751 | 0.193500339 | 0.356661737 |
| CKMT2-AS1 | 2.646455071 | 2.923172187 | 0.143473651 | 0.194158708 | 0.357433972 |
| AL606834.1 | 1.021182641 | 1.133765977 | 0.150881962 | 0.19481872 | 0.358207326 |
| ATP6V1B1-AS1 | 0.674498902 | 0.858246877 | 0.347576609 | 0.19545765 | 0.358940063 |
| AC093726.2 | 0.60199376 | 0.666925412 | 0.147776888 | 0.196143264 | 0.359756625 |
| AC004130.2 | 0.555459979 | 0.682453988 | 0.297048812 | 0.197124633 | 0.36020386 |
| AC009237.15 | 1.164563216 | 1.310074497 | 0.169859897 | 0.197462793 | 0.36020386 |
| AC023983.2 | 1.663919214 | 1.400861243 | -0.248271327 | 0.197469424 | 0.36020386 |
| AC018809.2 | 0.573259288 | 0.658491035 | 0.199975975 | 0.197475226 | 0.36020386 |
| AP001007.1 | 0.864149566 | 0.658214272 | -0.392723725 | 0.19759341 | 0.36020386 |
| AL603839.3 | 0.664597638 | 0.714267276 | 0.103982861 | 0.199484933 | 0.362225588 |
| AC005076.2 | 0.979638831 | 1.216241419 | 0.31210776 | 0.199484933 | 0.362225588 |
| AC055854.1 | 0.400420831 | 1.048846006 | 1.389213938 | 0.199690303 | 0.362225588 |
| AC012442.2 | 0.388912819 | 0.426248632 | 0.132248415 | 0.200158145 | 0.362225588 |
| LINC01578 | 6.087969965 | 5.747650007 | -0.082989027 | 0.200158145 | 0.362225588 |
| AL606760.1 | 0.604231329 | 0.700934232 | 0.214178096 | 0.200158145 | 0.362225588 |
| AC254633.1 | 0.644373499 | 1.231585749 | 0.934548013 | 0.200714311 | 0.362567944 |
| AC018645.3 | 5.675671449 | 6.06556085 | 0.095849972 | 0.200833014 | 0.362567944 |
| AC080013.4 | 1.0456796 | 1.1186171 | 0.097275417 | 0.201509542 | 0.363256657 |
| AC006058.1 | 1.323055855 | 1.659314799 | 0.326713646 | 0.20218773 | 0.363256657 |
| AC024075.2 | 3.809971732 | 3.423851488 | -0.154160169 | 0.20218773 | 0.363256657 |
| SNHG5 | 32.49772144 | 28.30220041 | -0.199424345 | 0.20218773 | 0.363256657 |
| AC015802.1 | 0.353693771 | 0.482776865 | 0.44885573 | 0.202510229 | 0.363398764 |
| AL596087.2 | 0.43944606 | 0.530372323 | 0.271319402 | 0.204586633 | 0.366684086 |
| AC100791.3 | 0.557098686 | 0.551077753 | -0.015677028 | 0.205171812 | 0.367291984 |
| AP000786.1 | 0.568012682 | 0.601805467 | 0.083374072 | 0.206206221 | 0.367532361 |
| AL035541.1 | 4.872634183 | 7.156264919 | 0.554504881 | 0.206278589 | 0.367532361 |
| AP002336.2 | 0.458887553 | 0.407562681 | -0.171118723 | 0.206289721 | 0.367532361 |
| AC090204.1 | 4.640880482 | 6.074008275 | 0.38825033 | 0.20629077 | 0.367532361 |
| AL121899.1 | 0.69989394 | 0.692202728 | -0.015941689 | 0.207200547 | 0.36871325 |
| AC097468.3 | 3.185752498 | 3.388005094 | 0.088801856 | 0.207673202 | 0.369114393 |
| AC009275.1 | 0.799281039 | 0.934732929 | 0.225851354 | 0.208019488 | 0.369290245 |
| AP006621.2 | 0.992423752 | 1.159186103 | 0.224084035 | 0.208353784 | 0.369444417 |
| FBXL19-AS1 | 0.671858069 | 0.750213578 | 0.159144879 | 0.209061282 | 0.370259187 |
| AL035587.1 | 1.65845998 | 1.443839656 | -0.199933664 | 0.209757838 | 0.370613552 |
| DGCR11 | 1.016208094 | 1.084031558 | 0.093210897 | 0.209757838 | 0.370613552 |
| SCGB1B2P | 1.280508589 | 0.514903763 | -1.314342211 | 0.210455017 | 0.37096846 |
| AL022328.1 | 0.81368177 | 1.056873641 | 0.377266325 | 0.21045565 | 0.37096846 |
| PANK2-AS1 | 1.158376116 | 1.329804241 | 0.199110121 | 0.211155986 | 0.371764018 |
| HDAC4-AS1 | 0.716961222 | 0.873285185 | 0.284557776 | 0.211857477 | 0.372559732 |
| AC090409.1 | 0.361827681 | 0.887196817 | 1.293951407 | 0.214671909 | 0.37663743 |
| MED14OS | 0.697780313 | 0.604286454 | -0.207540292 | 0.214680812 | 0.37663743 |
| AC008966.1 | 0.898505029 | 0.952135803 | 0.083640781 | 0.215390838 | 0.377358029 |
| AC084357.2 | 0.581832084 | 0.642149807 | 0.14230705 | 0.216100432 | 0.377358029 |
| ZNF503-AS2 | 1.056749269 | 1.194039121 | 0.176216991 | 0.216102555 | 0.377358029 |
| LENG8-AS1 | 1.500082151 | 1.56825016 | 0.064114199 | 0.216102555 | 0.377358029 |
| SLC25A21-AS1 | 0.480331931 | 0.441541155 | -0.121483804 | 0.216729251 | 0.377901609 |
| AC083809.1 | 0.562160714 | 0.553360632 | -0.022762627 | 0.217147329 | 0.377901609 |
| LINC01291 | 1.428046573 | 1.723643023 | 0.271417983 | 0.217173195 | 0.377901609 |
| LINC01535 | 0.495416501 | 0.444740081 | -0.155679491 | 0.217528937 | 0.37807998 |
| AC009318.3 | 0.648760788 | 0.720142848 | 0.150596485 | 0.217888186 | 0.378264025 |
| ETV5-AS1 | 0.466937995 | 0.581783611 | 0.31725167 | 0.218558448 | 0.378986948 |
| AC115618.2 | 10.92143809 | 12.22813339 | 0.163041358 | 0.218966351 | 0.379253783 |
| AC007773.1 | 0.529427904 | 0.642871043 | 0.280095132 | 0.219326126 | 0.379436739 |
| ATP2B1-AS1 | 0.666475136 | 0.533919479 | -0.319928868 | 0.222584291 | 0.384627715 |
| RARA-AS1 | 1.968381983 | 1.844936075 | -0.093439387 | 0.224775489 | 0.387965093 |
| AL031670.1 | 0.506796282 | 0.583550721 | 0.203452116 | 0.225500294 | 0.388333782 |
| NIFK-AS1 | 2.192737534 | 2.323342062 | 0.083468441 | 0.225509303 | 0.388333782 |
| RASSF1-AS1 | 0.515694081 | 0.638647731 | 0.308504894 | 0.226982057 | 0.389522082 |
| COX10-AS1 | 0.707915222 | 0.658994712 | -0.103309709 | 0.226982057 | 0.389522082 |
| AC007663.4 | 0.550626777 | 0.59400594 | 0.109402588 | 0.226982057 | 0.389522082 |
| AL355596.1 | 2.005503749 | 2.05019654 | 0.031797555 | 0.227598444 | 0.390131432 |
| LINC01615 | 0.567488492 | 1.165679169 | 1.038507727 | 0.228087352 | 0.390428239 |
| CNIH3-AS1 | 1.901823705 | 3.108070702 | 0.708635805 | 0.22829461 | 0.390428239 |
| AL049840.5 | 2.297001251 | 2.495269137 | 0.119443789 | 0.229204028 | 0.391535028 |
| AC026801.2 | 1.819864684 | 1.659311581 | -0.133246366 | 0.229948114 | 0.391909286 |
| AC005332.3 | 3.640754498 | 4.051812719 | 0.154330031 | 0.229948114 | 0.391909286 |
| AL136018.1 | 2.12217082 | 2.716686331 | 0.356307214 | 0.230571009 | 0.392481999 |
| IGFL2-AS1 | 0.326029025 | 0.56992855 | 0.805780658 | 0.23080991 | 0.392481999 |
| RASAL2-AS1 | 0.86538633 | 1.014290341 | 0.229054446 | 0.231441436 | 0.39310815 |
| AC107464.2 | 0.633661546 | 0.335691148 | -0.916577976 | 0.232818471 | 0.394758068 |
| AC024060.2 | 3.245714276 | 3.446311315 | 0.086517027 | 0.232941633 | 0.394758068 |
| LINC02683 | 0.796890776 | 0.783359824 | -0.02470686 | 0.233238513 | 0.394813038 |
| AC022306.2 | 1.263641506 | 1.107039221 | -0.190880894 | 0.234448714 | 0.396344047 |
| AC022075.1 | 1.742953525 | 1.00974325 | -0.7875456 | 0.234825479 | 0.396344047 |
| AC131235.3 | 0.434111694 | 0.496766957 | 0.194502928 | 0.235202675 | 0.396344047 |
| JPX | 3.087323164 | 3.123936237 | 0.017008503 | 0.235204839 | 0.396344047 |
| LINC00265 | 2.238282606 | 1.994052103 | -0.166689096 | 0.235962689 | 0.397172825 |
| AC124067.2 | 0.895143168 | 0.795635936 | -0.170010005 | 0.236698422 | 0.39796255 |
| HCG15 | 0.701387386 | 0.597345505 | -0.231645856 | 0.240545634 | 0.403522788 |
| AC144652.1 | 1.453815236 | 1.700529052 | 0.226139724 | 0.240546069 | 0.403522788 |
| LINC00520 | 7.176670271 | 11.70711193 | 0.705998673 | 0.241306885 | 0.404344758 |
| NDUFV2-AS1 | 0.993659668 | 0.873587674 | -0.185799306 | 0.242861131 | 0.406037703 |
| LINC00339 | 11.00399564 | 12.04858494 | 0.130836245 | 0.242861131 | 0.406037703 |
| AC025174.1 | 0.826587569 | 0.136663345 | -2.596541328 | 0.244383933 | 0.40804052 |
| AL121894.2 | 0.456841871 | 0.478460929 | 0.066706235 | 0.245152095 | 0.40804052 |
| CPNE8-AS1 | 0.773533483 | 0.650376806 | -0.250187934 | 0.245185702 | 0.40804052 |
| IL10RB-DT | 0.434066901 | 0.392367153 | -0.145713145 | 0.245191813 | 0.40804052 |
| BANCR | 60.17623803 | 51.46764957 | -0.225528019 | 0.24597109 | 0.40804052 |
| AC000123.1 | 1.510680204 | 1.697944694 | 0.168591179 | 0.245971745 | 0.40804052 |
| VPS13B-DT | 2.116451744 | 2.340641097 | 0.14525614 | 0.245972182 | 0.40804052 |
| LINC02482 | 2.538559045 | 2.882010564 | 0.183065807 | 0.246754291 | 0.408821288 |
| ROR1-AS1 | 0.563200049 | 1.059300896 | 0.911393083 | 0.247498355 | 0.408821288 |
| AC132872.1 | 4.00057607 | 4.217302725 | 0.076112826 | 0.247538141 | 0.408821288 |
| AL354836.1 | 1.891699129 | 1.567095682 | -0.27158938 | 0.247538141 | 0.408821288 |
| TNFRSF14-AS1 | 1.55200547 | 1.921389106 | 0.308016071 | 0.248323732 | 0.409665561 |
| CHKB-DT | 1.35083232 | 1.59970064 | 0.243953349 | 0.249111066 | 0.410510841 |
| AL136295.6 | 0.932978959 | 0.82534742 | -0.176843012 | 0.250690965 | 0.412598807 |
| ADNP-AS1 | 1.207121433 | 1.111856428 | -0.118600306 | 0.251483532 | 0.412598807 |
| AC008870.2 | 0.508014518 | 0.532296398 | 0.067360077 | 0.251483532 | 0.412598807 |
| CDC37L1-DT | 0.817387966 | 0.939894558 | 0.201477914 | 0.251483532 | 0.412598807 |
| BX640514.2 | 0.187550811 | 0.740164843 | 1.980565108 | 0.252510524 | 0.413828992 |
| AC064875.1 | 1.945118767 | 2.407022008 | 0.307391085 | 0.253073906 | 0.414297525 |
| AC107959.1 | 0.509133196 | 0.566797334 | 0.154789839 | 0.253871715 | 0.41514838 |
| SNHG20 | 2.630657805 | 2.884138358 | 0.13271678 | 0.254671273 | 0.41600023 |
| AC006504.2 | 0.62724652 | 0.510893655 | -0.296009543 | 0.256128982 | 0.416694056 |
| LINC01914 | 0.337790771 | 0.806038678 | 1.254719155 | 0.256225618 | 0.416694056 |
| AC090617.5 | 1.320233108 | 1.252942875 | -0.075472043 | 0.256275639 | 0.416694056 |
| SBNO1-AS1 | 0.418237848 | 0.465152412 | 0.153379883 | 0.256275639 | 0.416694056 |
| MID1IP1-AS1 | 1.143970776 | 1.364419542 | 0.254237125 | 0.256676725 | 0.416694056 |
| AC005332.6 | 10.61347976 | 11.22061404 | 0.08025389 | 0.257080449 | 0.416694056 |
| AC112491.1 | 1.750252058 | 1.5419444 | -0.182811958 | 0.257080449 | 0.416694056 |
| Z95115.1 | 1.478557635 | 1.600242615 | 0.114100169 | 0.257887011 | 0.416694056 |
| AC006435.2 | 1.346268196 | 1.125168635 | -0.258824602 | 0.257887011 | 0.416694056 |
| AL008729.1 | 0.832683068 | 1.079136695 | 0.374038232 | 0.257887011 | 0.416694056 |
| MATN1-AS1 | 0.520113922 | 0.53183282 | 0.032145156 | 0.258695327 | 0.417548241 |
| AC107032.2 | 0.475861485 | 0.498373245 | 0.066684928 | 0.259090893 | 0.417735101 |
| NORAD | 65.61669169 | 69.24952267 | 0.07774127 | 0.259505397 | 0.417952058 |
| SNHG10 | 1.686815601 | 1.785232276 | 0.081809525 | 0.260317222 | 0.418807772 |
| AL590705.3 | 0.717967947 | 0.80535001 | 0.165696485 | 0.26072269 | 0.419008586 |
| AC002310.1 | 0.733768434 | 0.831749161 | 0.180823662 | 0.261946139 | 0.420477501 |
| AP005436.3 | 0.530534279 | 0.399212782 | -0.410288057 | 0.262311967 | 0.420477501 |
| AC009831.1 | 0.697112928 | 0.606080136 | -0.201883823 | 0.262763234 | 0.420477501 |
| EBLN3P | 6.180049937 | 6.443811608 | 0.06029582 | 0.262763234 | 0.420477501 |
| AC036176.1 | 1.681201667 | 1.582928276 | -0.086896905 | 0.263582086 | 0.42097257 |
| MYCNUT | 0.687674126 | 1.295449207 | 0.913655479 | 0.263636539 | 0.42097257 |
| LINC00920 | 6.441435476 | 7.443089125 | 0.208519282 | 0.264402698 | 0.421668212 |
| GDNF-AS1 | 0.748565293 | 0.464691182 | -0.687855892 | 0.265001377 | 0.421668212 |
| DM1-AS | 0.582486943 | 0.613820768 | 0.075591747 | 0.265225069 | 0.421668212 |
| AC002128.1 | 0.647285089 | 0.65564257 | 0.018508259 | 0.26563581 | 0.421668212 |
| AL589843.1 | 1.559299717 | 1.550026557 | -0.008605324 | 0.266008968 | 0.421668212 |
| CIRBP-AS1 | 1.515781316 | 1.687475662 | 0.154805066 | 0.2660492 | 0.421668212 |
| ZSCAN16-AS1 | 4.813118679 | 5.170092668 | 0.103218143 | 0.2660492 | 0.421668212 |
| LINC02251 | 1.551670874 | 1.092277657 | -0.506482943 | 0.267506788 | 0.423390044 |
| PTOV1-AS1 | 2.882305691 | 3.036033024 | 0.074964131 | 0.267702747 | 0.423390044 |
| AC020915.2 | 0.433389564 | 0.376809321 | -0.201829762 | 0.268107942 | 0.423582177 |
| RAB11B-AS1 | 1.61483018 | 1.718839656 | 0.090052513 | 0.268532164 | 0.423803933 |
| NRSN2-AS1 | 2.846293825 | 3.073303944 | 0.110705854 | 0.270196287 | 0.425979997 |
| TOLLIP-AS1 | 1.372471997 | 1.536041959 | 0.162440911 | 0.271030996 | 0.426845228 |
| TGFB2-AS1 | 0.523869054 | 0.424387903 | -0.303822708 | 0.27275692 | 0.429110729 |
| AC093788.1 | 0.497199141 | 0.570340339 | 0.197999272 | 0.273505345 | 0.429835242 |
| AC245297.4 | 1.414301849 | 1.604318696 | 0.181870698 | 0.276076337 | 0.432509938 |
| LINC01356 | 0.485424453 | 0.450784839 | -0.106807789 | 0.276076337 | 0.432509938 |
| AC004982.2 | 0.452930587 | 0.473456315 | 0.063941348 | 0.276076337 | 0.432509938 |
| AL132989.1 | 1.168476449 | 1.213737184 | 0.054827406 | 0.278620657 | 0.435129664 |
| LPP-AS2 | 1.157068913 | 1.394351176 | 0.269119167 | 0.278622879 | 0.435129664 |
| H1FX-AS1 | 0.543629156 | 0.581911811 | 0.098177695 | 0.278622879 | 0.435129664 |
| AC099522.2 | 0.870308234 | 0.875117446 | 0.007950203 | 0.279475267 | 0.436004779 |
| AC068234.2 | 2.953034093 | 3.078662856 | 0.060105874 | 0.279901013 | 0.436213166 |
| AP001107.4 | 0.47314489 | 0.47865609 | 0.016707422 | 0.280328981 | 0.436392658 |
| AC079336.2 | 0.535344913 | 0.464180246 | -0.205783566 | 0.281179128 | 0.436392658 |
| AC093673.1 | 5.839801073 | 8.440295112 | 0.531374217 | 0.281185356 | 0.436392658 |
| Z97989.1 | 0.642434304 | 0.620575099 | -0.049943121 | 0.281185356 | 0.436392658 |
| AC005911.1 | 0.613286577 | 0.579951022 | -0.080630309 | 0.282472464 | 0.437934983 |
| AP000704.2 | 0.508520384 | 0.681297722 | 0.421979778 | 0.283761557 | 0.438778863 |
| AC135178.6 | 0.871818966 | 0.916417809 | 0.071976906 | 0.283763783 | 0.438778863 |
| ALG13-AS1 | 0.458925296 | 0.483709637 | 0.075881949 | 0.284012471 | 0.438778863 |
| AL035446.1 | 0.741927159 | 1.604775272 | 1.113021822 | 0.284192338 | 0.438778863 |
| AC027682.6 | 0.968642608 | 0.917332424 | -0.078519831 | 0.285491601 | 0.440329505 |
| AC093635.1 | 0.402189355 | 0.505101704 | 0.328699011 | 0.285924554 | 0.440542166 |
| ZNNT1 | 1.336252818 | 1.45273021 | 0.12057381 | 0.286358172 | 0.440755414 |
| AC027307.2 | 10.51914933 | 10.7442696 | 0.03054937 | 0.287226518 | 0.441586515 |
| AC011477.2 | 1.187401892 | 1.044690605 | -0.184732579 | 0.288090398 | 0.441586515 |
| AP003486.1 | 1.391210933 | 1.251771802 | -0.152369592 | 0.288096639 | 0.441586515 |
| AC092614.1 | 0.473894997 | 0.427120097 | -0.14992565 | 0.288967422 | 0.441586515 |
| PCCA-DT | 5.012979731 | 5.316394925 | 0.084779877 | 0.288968536 | 0.441586515 |
| AC026979.2 | 2.715414279 | 2.558597416 | -0.085819157 | 0.288968536 | 0.441586515 |
| AC009061.2 | 0.605599149 | 0.669753993 | 0.145268098 | 0.288968536 | 0.441586515 |
| AC139887.1 | 0.432675455 | 0.490239603 | 0.180201754 | 0.289693876 | 0.442016771 |
| AC012181.2 | 0.667333969 | 0.692192405 | 0.05276417 | 0.28984221 | 0.442016771 |
| AC002398.1 | 1.700261433 | 1.782881753 | 0.068454429 | 0.29071766 | 0.442447978 |
| AC083862.1 | 1.740621777 | 1.65289612 | -0.074606693 | 0.29071766 | 0.442447978 |
| AC008991.1 | 1.934268323 | 1.458526916 | -0.40727593 | 0.291158122 | 0.442667084 |
| AL355001.2 | 2.196412784 | 2.03440027 | -0.110545655 | 0.291594887 | 0.44286361 |
| AC022211.3 | 0.72300691 | 0.820143363 | 0.181866681 | 0.292034055 | 0.44286361 |
| AP001542.3 | 1.232494184 | 0.981522445 | -0.328487675 | 0.292473891 | 0.44286361 |
| AC108449.2 | 2.719745586 | 2.303363373 | -0.239729678 | 0.292473891 | 0.44286361 |
| AC024896.1 | 8.986590713 | 9.679285119 | 0.107126601 | 0.29600769 | 0.447760365 |
| AC093382.1 | 0.426962876 | 0.536278056 | 0.328870586 | 0.297289384 | 0.449243978 |
| AL359962.2 | 0.436798205 | 0.405985313 | -0.105539387 | 0.29812207 | 0.449924248 |
| TRAM2-AS1 | 2.51033753 | 2.730569063 | 0.12132029 | 0.298676717 | 0.449924248 |
| AC069281.2 | 1.705095984 | 1.786758654 | 0.067491821 | 0.298676717 | 0.449924248 |
| MIF-AS1 | 0.697848716 | 0.738654392 | 0.081985188 | 0.299569953 | 0.449924248 |
| LINC02099 | 3.300860159 | 3.55958588 | 0.108867389 | 0.300013327 | 0.449924248 |
| MIR5689HG | 0.491268814 | 0.627023921 | 0.352007823 | 0.300414125 | 0.449924248 |
| AC009495.2 | 2.050388541 | 1.686324643 | -0.282015017 | 0.300461057 | 0.449924248 |
| LINC01311 | 0.572588516 | 0.61074918 | 0.093081285 | 0.300464968 | 0.449924248 |
| LINC01852 | 0.442832253 | 0.416164874 | -0.0896051 | 0.301361764 | 0.449924248 |
| AC008946.1 | 0.938415405 | 1.06185503 | 0.178288213 | 0.301361764 | 0.449924248 |
| PAXIP1-AS2 | 1.421303664 | 1.562897276 | 0.137008136 | 0.301361764 | 0.449924248 |
| AP003392.5 | 0.449334439 | 0.451821561 | 0.007963475 | 0.301809712 | 0.449924248 |
| AL590560.3 | 1.107649407 | 1.150014847 | 0.054151175 | 0.302259223 | 0.449924248 |
| AC114341.1 | 0.489352913 | 0.412809423 | -0.245399384 | 0.302259223 | 0.449924248 |
| AC004492.1 | 0.557545604 | 0.595657652 | 0.095393581 | 0.302259893 | 0.449924248 |
| AC079949.2 | 1.102679734 | 1.519380168 | 0.462469064 | 0.30495334 | 0.453481411 |
| AC107884.1 | 0.343403376 | 0.687411103 | 1.001268933 | 0.305872457 | 0.4543956 |
| FAM222A-AS1 | 0.509685122 | 0.480253727 | -0.085809431 | 0.307679811 | 0.456185017 |
| AC009054.2 | 0.506653104 | 0.547177759 | 0.111011292 | 0.307688086 | 0.456185017 |
| SEMA3B-AS1 | 0.720736128 | 0.533478613 | -0.43404073 | 0.308472726 | 0.4566305 |
| AC021242.3 | 0.429052497 | 0.384703188 | -0.157408397 | 0.308600251 | 0.4566305 |
| AC010245.2 | 0.571415328 | 0.588727303 | 0.043059801 | 0.310427578 | 0.458879578 |
| AL358472.2 | 1.056029315 | 1.095766476 | 0.053290486 | 0.311344082 | 0.459779144 |
| AC139887.2 | 0.794878672 | 0.892241739 | 0.166699969 | 0.313182215 | 0.462036608 |
| AL645608.7 | 0.594196955 | 0.416700753 | -0.511929507 | 0.314102836 | 0.46248245 |
| AC127024.5 | 1.40826246 | 1.489254712 | 0.080674287 | 0.314103955 | 0.46248245 |
| AC073195.2 | 0.476719858 | 0.479646313 | 0.008829243 | 0.315027478 | 0.463385245 |
| IDI2-AS1 | 3.099271303 | 3.978666329 | 0.360355862 | 0.315952783 | 0.464288882 |
| AL096828.3 | 0.485864344 | 0.534439025 | 0.137471797 | 0.316415992 | 0.464512366 |
| TP53TG1 | 14.47845405 | 13.09411401 | -0.14498912 | 0.31687987 | 0.464736391 |
| LINC02611 | 0.466120733 | 0.367926675 | -0.34128541 | 0.319671829 | 0.468370992 |
| LINC00681 | 12.198671 | 8.701381511 | -0.4874076 | 0.32329185 | 0.472841948 |
| AL512329.2 | 2.06229177 | 2.985375653 | 0.533664022 | 0.32335675 | 0.472841948 |
| DLG5-AS1 | 0.505669574 | 0.534227668 | 0.079259719 | 0.324359626 | 0.473382787 |
| TMEM99 | 5.05996834 | 6.036044048 | 0.254474976 | 0.324360745 | 0.473382787 |
| CRNDE | 6.0939158 | 7.214081295 | 0.243446114 | 0.325303875 | 0.473832863 |
| AC145098.1 | 1.31220174 | 0.938224876 | -0.483983882 | 0.325303875 | 0.473832863 |
| AC021078.1 | 1.011720583 | 1.071030189 | 0.082188245 | 0.327195481 | 0.476123639 |
| LINC00638 | 0.815268245 | 0.72668056 | -0.165953511 | 0.328143957 | 0.477038878 |
| AC113194.1 | 1.673878575 | 1.264133033 | -0.405046581 | 0.330014279 | 0.478406852 |
| AC008771.1 | 2.876333023 | 3.258245196 | 0.179864455 | 0.330046254 | 0.478406852 |
| PAXIP1-AS1 | 4.241807186 | 4.068026511 | -0.060349963 | 0.330046254 | 0.478406852 |
| ATP1B3-AS1 | 0.514368997 | 0.598591397 | 0.218767856 | 0.330908842 | 0.478859605 |
| ERICH6-AS1 | 1.117819296 | 1.198284989 | 0.100284081 | 0.331000075 | 0.478859605 |
| ANKRD10-IT1 | 6.365230362 | 7.54208931 | 0.244751507 | 0.331955677 | 0.479777179 |
| AP005482.4 | 1.238086013 | 1.120267374 | -0.144268444 | 0.333872224 | 0.482069355 |
| AC139720.1 | 0.816728165 | 0.916972742 | 0.16702287 | 0.334320515 | 0.482069355 |
| AC011477.1 | 1.679036937 | 1.471743129 | -0.190108076 | 0.334833169 | 0.482069355 |
| KCTD21-AS1 | 1.425623294 | 0.661613794 | -1.107531596 | 0.334833169 | 0.482069355 |
| AL390955.2 | 0.718329418 | 0.838463495 | 0.223102374 | 0.336760398 | 0.483910755 |
| LINC02062 | 0.759884256 | 0.775874352 | 0.030043348 | 0.336760398 | 0.483910755 |
| LINC00641 | 1.631582538 | 1.567552467 | -0.057758241 | 0.338694747 | 0.485624346 |
| AL357140.2 | 0.6800387 | 0.74670159 | 0.134914954 | 0.338694747 | 0.485624346 |
| LINC02100 | 0.378781721 | 0.490090692 | 0.371682036 | 0.338928713 | 0.485624346 |
| ILF3-DT | 11.43920788 | 10.90765319 | -0.068646419 | 0.33966459 | 0.48620446 |
| AL358216.1 | 1.024270186 | 1.36778746 | 0.417247743 | 0.340146272 | 0.48620446 |
| MIR1915HG | 0.657014109 | 0.817958046 | 0.316102497 | 0.340636213 | 0.48620446 |
| L3MBTL2-AS1 | 0.96456187 | 0.891429937 | -0.113752368 | 0.340636213 | 0.48620446 |
| AL133243.2 | 0.816221038 | 0.849772385 | 0.058116565 | 0.341609614 | 0.487128131 |
| KCNK15-AS1 | 0.532290214 | 0.492463486 | -0.112196284 | 0.343557845 | 0.488977781 |
| AC010168.2 | 0.520347082 | 0.440632649 | -0.239897855 | 0.34356175 | 0.488977781 |
| ST20-AS1 | 1.981305503 | 1.743088727 | -0.184805343 | 0.346503285 | 0.492694671 |
| LINC01150 | 0.493195429 | 0.433446236 | -0.186306374 | 0.348147035 | 0.493680281 |
| AL138995.1 | 0.487393874 | 0.465612085 | -0.065959615 | 0.348473191 | 0.493680281 |
| AL512408.1 | 0.571612381 | 0.626805713 | 0.132981164 | 0.348473191 | 0.493680281 |
| LINC01050 | 0.652922531 | 0.973417533 | 0.576146935 | 0.3485191 | 0.493680281 |
| AC121247.2 | 0.340134353 | 0.465563085 | 0.452871949 | 0.350409657 | 0.495006761 |
| AL451085.3 | 0.788240201 | 0.861961998 | 0.128988935 | 0.350450199 | 0.495006761 |
| BX284668.5 | 5.99161132 | 5.313265498 | -0.173345234 | 0.350450199 | 0.495006761 |
| AL049840.6 | 11.29355877 | 12.1799631 | 0.10900959 | 0.352434305 | 0.496869138 |
| AC138207.2 | 0.712385951 | 0.718789032 | 0.01290933 | 0.352434305 | 0.496869138 |
| AL109811.2 | 2.080362018 | 2.147814229 | 0.046034612 | 0.353429019 | 0.497801439 |
| LINC01614 | 0.823198179 | 0.926351743 | 0.17032031 | 0.354200233 | 0.498417482 |
| AC073842.2 | 0.724683964 | 0.860473042 | 0.247778025 | 0.355423766 | 0.499668251 |
| RAB30-DT | 1.757561204 | 1.895085508 | 0.108688016 | 0.358429175 | 0.50341934 |
| AC002550.2 | 0.899675421 | 0.950307233 | 0.078989401 | 0.35943452 | 0.503883323 |
| AP003068.1 | 0.75002037 | 0.720054562 | -0.058823547 | 0.35943452 | 0.503883323 |
| AC093752.3 | 0.658494057 | 0.609333009 | -0.111939522 | 0.359937745 | 0.504115435 |
| MIR3936HG | 0.413760937 | 0.398583042 | -0.053917116 | 0.360441635 | 0.504348042 |
| AC018690.1 | 0.803279939 | 0.900049173 | 0.164100976 | 0.366521455 | 0.511895727 |
| AL451165.2 | 3.28101463 | 3.104893157 | -0.079598402 | 0.366521455 | 0.511895727 |
| AP001432.1 | 0.551805619 | 0.510389529 | -0.112561418 | 0.3670271 | 0.51212286 |
| AC008443.5 | 1.629702015 | 1.790787567 | 0.13598601 | 0.367540941 | 0.512360994 |
| AL136040.1 | 0.440578813 | 0.463259803 | 0.072421386 | 0.369585206 | 0.514730142 |
| AL031186.1 | 0.886883314 | 0.923043474 | 0.057654296 | 0.371636524 | 0.516623212 |
| AL118558.4 | 0.780218871 | 0.724212035 | -0.10746674 | 0.371636524 | 0.516623212 |
| LINC01138 | 0.914051659 | 0.920902562 | 0.010772815 | 0.372664825 | 0.517570776 |
| SAMMSON | 1.552971032 | 0.84307175 | -0.881303597 | 0.373681633 | 0.518037575 |
| AC006504.7 | 2.246048265 | 2.008920257 | -0.160968631 | 0.373694888 | 0.518037575 |
| SNHG4 | 2.613385603 | 3.155967031 | 0.272162126 | 0.374726711 | 0.518986067 |
| AL139089.1 | 0.781722066 | 0.811968623 | 0.054768216 | 0.375760293 | 0.519935235 |
| AC063923.2 | 1.816020798 | 2.945499715 | 0.697731688 | 0.376139602 | 0.519978172 |
| Z82246.1 | 1.256730355 | 1.618757774 | 0.365211984 | 0.378648424 | 0.522786766 |
| LINC02609 | 1.711854065 | 1.791954758 | 0.065974496 | 0.378871588 | 0.522786766 |
| AC008035.1 | 0.565094159 | 0.525691117 | -0.104275919 | 0.380954126 | 0.524691114 |
| LINC00174 | 1.697191554 | 1.611613133 | -0.074643937 | 0.380954566 | 0.524691114 |
| AC073896.3 | 1.309538343 | 1.207226638 | -0.117361756 | 0.381476297 | 0.524925449 |
| AC116407.2 | 0.630872424 | 0.640017308 | 0.020762631 | 0.381996486 | 0.525014962 |
| AC062037.3 | 0.608110743 | 0.683320415 | 0.168228153 | 0.382244651 | 0.525014962 |
| AC009022.1 | 0.507216032 | 0.515680045 | 0.023875871 | 0.383044561 | 0.525630082 |
| AL162457.2 | 8.212573895 | 14.33271329 | 0.803405397 | 0.384647169 | 0.527344558 |
| AL022322.1 | 0.578705238 | 0.728476255 | 0.332053247 | 0.386190498 | 0.528492844 |
| LINC00909 | 2.85690593 | 2.77574374 | -0.04157915 | 0.386192694 | 0.528492844 |
| LINC01389 | 0.519772453 | 0.580338711 | 0.15901499 | 0.38718411 | 0.529364355 |
| Z73429.1 | 0.38564246 | 0.494053712 | 0.35740399 | 0.387940592 | 0.529435797 |
| AC110285.1 | 15.97155659 | 20.22446065 | 0.340596304 | 0.388300199 | 0.529435797 |
| ZNF528-AS1 | 1.067017675 | 1.013554097 | -0.074160982 | 0.388300199 | 0.529435797 |
| LINC00324 | 1.19914615 | 1.014127119 | -0.241769 | 0.389356574 | 0.530391756 |
| PAX8-AS1 | 0.53227693 | 0.736026526 | 0.467580723 | 0.390414695 | 0.530864426 |
| USP27X-AS1 | 0.574708753 | 0.592117368 | 0.04305215 | 0.390414695 | 0.530864426 |
| AL139288.1 | 0.83429386 | 0.780043686 | -0.097000703 | 0.391474452 | 0.531821071 |
| AC008669.1 | 0.583542708 | 0.503843594 | -0.211862292 | 0.392536173 | 0.532294737 |
| AC135050.6 | 10.44316648 | 9.747906282 | -0.099394934 | 0.392536173 | 0.532294737 |
| LINC01443 | 11.18989629 | 14.97667825 | 0.420521012 | 0.394590297 | 0.534209115 |
| AC010809.1 | 0.431543392 | 0.470103549 | 0.123472939 | 0.394663532 | 0.534209115 |
| AC092535.5 | 3.536669416 | 2.534185453 | -0.480869267 | 0.398942418 | 0.539023556 |
| URB1-AS1 | 5.961331125 | 6.337444038 | 0.088266592 | 0.398942418 | 0.539023556 |
| AP000894.4 | 1.173416725 | 0.994691803 | -0.238393967 | 0.400016211 | 0.539985717 |
| AC026740.1 | 1.793170176 | 1.332366153 | -0.4285218 | 0.40109174 | 0.540948481 |
| LINC01971 | 0.403507937 | 0.446525755 | 0.146146338 | 0.402658799 | 0.542571829 |
| AC139768.1 | 1.944630752 | 1.819311535 | -0.096103634 | 0.404328731 | 0.543350851 |
| AL359711.2 | 0.482688478 | 0.446247032 | -0.113249814 | 0.404328731 | 0.543350851 |
| BX322234.1 | 1.064915672 | 1.415604392 | 0.410678951 | 0.404328731 | 0.543350851 |
| AC008537.2 | 0.531894744 | 0.614594298 | 0.208493602 | 0.406491585 | 0.54576613 |
| TENM3-AS1 | 0.29471086 | 0.545507245 | 0.888298133 | 0.409745331 | 0.549164283 |
| AL596094.1 | 0.796376895 | 0.829980278 | 0.059625691 | 0.409758213 | 0.549164283 |
| SNHG30 | 5.608584901 | 5.884336994 | 0.06924306 | 0.410849419 | 0.550132899 |
| DLEU1 | 0.963195353 | 1.034514383 | 0.103053367 | 0.41194224 | 0.551101939 |
| AL118558.3 | 2.193359381 | 2.100273458 | -0.062565035 | 0.413036783 | 0.551848531 |
| LINC00322 | 0.717898091 | 0.709806905 | -0.016352452 | 0.413239556 | 0.551848531 |
| AL355574.1 | 1.686874927 | 1.739072258 | 0.043964868 | 0.414133047 | 0.552547488 |
| TPT1-AS1 | 1.517633214 | 1.77250577 | 0.223967165 | 0.415231031 | 0.553517794 |
| AC105206.2 | 0.993239697 | 1.216858342 | 0.292947401 | 0.416330625 | 0.55399446 |
| AL035071.1 | 3.963925136 | 3.888515802 | -0.027710114 | 0.416330733 | 0.55399446 |
| AC073409.1 | 2.157618002 | 0.393040068 | -2.456691166 | 0.418127062 | 0.555889318 |
| EXOSC10-AS1 | 1.171153641 | 1.343467426 | 0.198030989 | 0.418535286 | 0.555936995 |
| AL008723.2 | 0.923366234 | 0.867594005 | -0.089882893 | 0.421223952 | 0.558359457 |
| AC011466.3 | 0.399282977 | 0.621441956 | 0.638208082 | 0.421282905 | 0.558359457 |
| ARHGAP27P1-BPTFP1-KPNA2P3 | 0.578393991 | 0.581234949 | 0.007068889 | 0.421854968 | 0.558359457 |
| LINC01547 | 1.400020366 | 1.302357178 | -0.104322645 | 0.421854968 | 0.558359457 |
| GABPB1-AS1 | 1.444089908 | 1.4966303 | 0.051557322 | 0.42407664 | 0.560802856 |
| AC027702.2 | 0.541805232 | 0.510774188 | -0.085088706 | 0.428540455 | 0.565703713 |
| ZNF790-AS1 | 1.191255751 | 1.120511877 | -0.088325237 | 0.428540455 | 0.565703713 |
| AC100814.2 | 1.624653966 | 1.415236539 | -0.199089271 | 0.429100241 | 0.565942279 |
| LINC00456 | 0.990584987 | 0.992350125 | 0.002568472 | 0.431365623 | 0.568137381 |
| NR4A1AS | 0.638086485 | 0.690435317 | 0.113754286 | 0.431905755 | 0.568137381 |
| AP001469.3 | 0.646626327 | 0.715932174 | 0.146890668 | 0.431906181 | 0.568137381 |
| LINC00235 | 0.416303973 | 0.407859722 | -0.029564286 | 0.435287171 | 0.572080762 |
| AC020558.2 | 0.602905327 | 0.53084072 | -0.183652434 | 0.436417552 | 0.572703698 |
| LINC00488 | 0.669776913 | 1.085238581 | 0.69625969 | 0.436528338 | 0.572703698 |
| AL021707.3 | 0.786995184 | 0.799610673 | 0.022942922 | 0.440387077 | 0.576964593 |
| AL390198.1 | 1.419118817 | 1.403318771 | -0.016152623 | 0.440932721 | 0.576964593 |
| AL391244.2 | 2.214975707 | 2.579712684 | 0.219919518 | 0.440955959 | 0.576964593 |
| AC091965.4 | 0.547252046 | 0.649196857 | 0.246450571 | 0.442088863 | 0.576964593 |
| MIR17HG | 0.332662456 | 0.494349258 | 0.571471616 | 0.44209435 | 0.576964593 |
| ASMTL-AS1 | 1.739407018 | 1.908332257 | 0.133716819 | 0.442094772 | 0.576964593 |
| SOS1-IT1 | 1.514023086 | 1.16453488 | -0.378633353 | 0.443235266 | 0.577947819 |
| AC092279.1 | 0.716149013 | 0.614520139 | -0.220799515 | 0.444377441 | 0.578931517 |
| AL135925.1 | 0.71924306 | 0.694660718 | -0.050170877 | 0.445521292 | 0.579915684 |
| AP001025.1 | 0.99701959 | 1.10556471 | 0.149089714 | 0.449730351 | 0.584884507 |
| CR936218.1 | 0.536423813 | 0.557269358 | 0.055001545 | 0.452300366 | 0.586282657 |
| AC024075.3 | 1.112725757 | 1.050773819 | -0.082645909 | 0.452419539 | 0.586282657 |
| AC091982.3 | 1.284463911 | 1.279199366 | -0.005925228 | 0.452419539 | 0.586282657 |
| SNHG8 | 42.13488214 | 39.6207289 | -0.088759668 | 0.452419539 | 0.586282657 |
| AC012640.4 | 1.177253911 | 1.268923343 | 0.108179401 | 0.453575085 | 0.586282657 |
| AC015912.3 | 2.666388793 | 1.875880211 | -0.507319455 | 0.453575085 | 0.586282657 |
| HOXC-AS1 | 1.861924976 | 1.700140276 | -0.131141157 | 0.454731878 | 0.586282657 |
| PRR34-AS1 | 4.163981516 | 4.321235133 | 0.053480071 | 0.454732295 | 0.586282657 |
| AL163051.1 | 0.578772564 | 0.558620369 | -0.051128356 | 0.454732295 | 0.586282657 |
| LINC01366 | 1.700095788 | 1.933406853 | 0.185529226 | 0.454732295 | 0.586282657 |
| AL031775.2 | 0.655439744 | 0.670347038 | 0.032445013 | 0.45530277 | 0.586511678 |
| PLCH1-AS1 | 1.768550222 | 1.468809307 | -0.267920083 | 0.456302056 | 0.586912869 |
| MIR3659HG | 0.358614776 | 0.464064332 | 0.371889884 | 0.456400429 | 0.586912869 |
| SCARNA9 | 0.672737635 | 0.588666749 | -0.19259283 | 0.45702881 | 0.587215157 |
| AL138976.2 | 1.148834253 | 1.09812907 | -0.065123036 | 0.458213887 | 0.587221745 |
| AC138230.1 | 0.433332297 | 0.446878565 | 0.044409078 | 0.458213887 | 0.587221745 |
| AC093157.1 | 1.528102077 | 1.600067453 | 0.066391806 | 0.458213887 | 0.587221745 |
| CCDC183-AS1 | 0.682547609 | 0.618163416 | -0.142941406 | 0.459377731 | 0.58770433 |
| LINC02367 | 3.074896689 | 3.680424315 | 0.259334165 | 0.459377731 | 0.58770433 |
| MMP25-AS1 | 0.924859313 | 0.927042926 | 0.00340222 | 0.46054323 | 0.58869096 |
| AC006026.3 | 0.622013534 | 0.577103969 | -0.108114718 | 0.46188423 | 0.589900047 |
| ZNF561-AS1 | 0.96094632 | 0.883849834 | -0.120654565 | 0.464049628 | 0.592159055 |
| PAN3-AS1 | 1.094140643 | 0.972166088 | -0.170523483 | 0.465221722 | 0.592641665 |
| NAPA-AS1 | 0.854606842 | 0.92240614 | 0.110141248 | 0.465221722 | 0.592641665 |
| AL031600.1 | 2.326194838 | 2.722489648 | 0.226954624 | 0.46639546 | 0.593124721 |
| AC008393.1 | 0.909463589 | 0.948193377 | 0.060165437 | 0.46639546 | 0.593124721 |
| AC027097.1 | 1.316778933 | 1.372262145 | 0.059542949 | 0.46757084 | 0.594113416 |
| AC011815.1 | 0.423735209 | 0.393479845 | -0.106873268 | 0.46874786 | 0.595102512 |
| AC020913.3 | 0.378989636 | 0.447783337 | 0.240642448 | 0.470143586 | 0.596069889 |
| HECW2-AS1 | 0.51812787 | 1.307736341 | 1.335691608 | 0.470770626 | 0.596069889 |
| AC009318.2 | 1.240022517 | 1.255767533 | 0.0182031 | 0.471106811 | 0.596069889 |
| AC124798.1 | 0.960369362 | 0.784216276 | -0.292337795 | 0.471106811 | 0.596069889 |
| MIR3681HG | 0.993725366 | 1.04849006 | 0.077394087 | 0.4758443 | 0.601554225 |
| AC093726.1 | 2.099474472 | 2.129606415 | 0.020558576 | 0.47703274 | 0.602546431 |
| AC138207.4 | 0.600998878 | 0.536694189 | -0.163262032 | 0.481802711 | 0.608057014 |
| AL117379.1 | 1.203968882 | 1.017428718 | -0.242870383 | 0.485397147 | 0.611559443 |
| AC011450.1 | 1.45978688 | 1.420888849 | -0.038964058 | 0.485397147 | 0.611559443 |
| AL391988.1 | 0.834099585 | 0.971032857 | 0.219300473 | 0.489006049 | 0.615586873 |
| AC009562.1 | 0.846346736 | 0.842138147 | -0.007191919 | 0.489445586 | 0.615621112 |
| AC106820.3 | 0.830644878 | 0.745218113 | -0.15656908 | 0.493840317 | 0.620103948 |
| AC046143.1 | 0.876305804 | 0.912784465 | 0.058839823 | 0.493840317 | 0.620103948 |
| AL359853.1 | 0.665433478 | 1.400018181 | 1.073079208 | 0.495570346 | 0.621753383 |
| ZNF426-DT | 0.776578353 | 0.749116782 | -0.051940849 | 0.49626661 | 0.622104155 |
| AL691432.2 | 3.151160062 | 2.989623837 | -0.075919066 | 0.497482735 | 0.623062065 |
| AC017076.1 | 0.661705753 | 0.748503972 | 0.177820148 | 0.498688134 | 0.623062065 |
| ZBED5-AS1 | 5.436164685 | 5.245902043 | -0.051398294 | 0.498700045 | 0.623062065 |
| AL354920.1 | 2.212239575 | 1.94750381 | -0.183881481 | 0.498700045 | 0.623062065 |
| AC002470.1 | 1.584584019 | 1.416205376 | -0.162073659 | 0.499918937 | 0.624062687 |
| AC010326.3 | 3.647951999 | 3.780357576 | 0.051435956 | 0.503585079 | 0.628114054 |
| AC008840.1 | 0.430403734 | 0.399121568 | -0.108862353 | 0.507263403 | 0.632173841 |
| AL035425.2 | 0.437935083 | 0.374886509 | -0.224263122 | 0.509157275 | 0.634004847 |
| AC106782.5 | 0.859413403 | 0.927119875 | 0.109403613 | 0.509726717 | 0.63418499 |
| AL139011.1 | 0.506391813 | 0.541329281 | 0.096252347 | 0.510342932 | 0.634422979 |
| AF001548.2 | 4.739626346 | 4.249939126 | -0.15733115 | 0.512194304 | 0.636194755 |
| LINC01144 | 0.830561574 | 0.966462108 | 0.218626044 | 0.51343043 | 0.637200026 |
| AC009509.5 | 1.080111367 | 1.169203289 | 0.11434572 | 0.515907333 | 0.639742233 |
| AC099518.6 | 0.497339968 | 0.467233303 | -0.090089267 | 0.51714268 | 0.640741927 |
| AC253536.6 | 0.668790838 | 0.748062884 | 0.161604469 | 0.518390324 | 0.640855802 |
| FP671120.8 | 0.431507729 | 0.373990299 | -0.206385552 | 0.51839042 | 0.640855802 |
| AL035665.1 | 0.330231278 | 1.883212311 | 2.511646978 | 0.51852231 | 0.640855802 |
| AC026356.2 | 0.447283695 | 0.478040291 | 0.09594205 | 0.51963418 | 0.641168574 |
| AC125807.2 | 1.629152759 | 1.682794895 | 0.046737461 | 0.519634277 | 0.641168574 |
| ZNF433-AS1 | 0.472028149 | 0.44127264 | -0.097202597 | 0.520879671 | 0.641644677 |
| NOP14-AS1 | 2.104406796 | 2.214745328 | 0.0737272 | 0.520879671 | 0.641644677 |
| LINC01010 | 0.858741997 | 0.993959847 | 0.210962823 | 0.522734783 | 0.643399036 |
| AL139349.1 | 0.614111426 | 0.508736414 | -0.271582082 | 0.524597641 | 0.644662723 |
| AL122010.1 | 2.498865123 | 2.658268642 | 0.089213876 | 0.524625056 | 0.644662723 |
| AC040977.1 | 4.546091839 | 4.917851241 | 0.113401266 | 0.528384187 | 0.648214947 |
| AL121603.2 | 0.82049212 | 0.859396942 | 0.066835165 | 0.528384187 | 0.648214947 |
| AC009495.1 | 2.788138214 | 2.56411219 | -0.120842694 | 0.529624571 | 0.64920319 |
| BET1-AS1 | 0.510698643 | 0.559569081 | 0.131844023 | 0.530265556 | 0.649455681 |
| AC007384.1 | 0.601427513 | 0.302530191 | -0.991311746 | 0.532129874 | 0.650704655 |
| AC073508.3 | 3.033162018 | 3.000339564 | -0.015696775 | 0.532156988 | 0.650704655 |
| MAN1B1-DT | 1.546579312 | 1.548968842 | 0.002227305 | 0.534679747 | 0.653254388 |
| DICER1-AS1 | 0.78825271 | 0.847596737 | 0.10471981 | 0.535943385 | 0.654262857 |
| AL031429.1 | 2.283744203 | 5.074019112 | 1.151727887 | 0.536825528 | 0.654736594 |
| AC025171.1 | 1.897324465 | 1.881527752 | -0.012061849 | 0.537208525 | 0.654736594 |
| AC139795.3 | 0.893665805 | 0.92644162 | 0.051964645 | 0.53847507 | 0.655744927 |
| AC091729.3 | 2.820285301 | 2.734014382 | -0.044820281 | 0.5397433 | 0.656753664 |
| ZNF529-AS1 | 1.228455715 | 1.204434283 | -0.028490172 | 0.542284047 | 0.659307885 |
| AC004918.5 | 0.729154395 | 0.744441258 | 0.029933683 | 0.543556654 | 0.6603174 |
| DNM1P35 | 0.538383176 | 0.532742115 | -0.015195994 | 0.545468254 | 0.662100897 |
| AC010834.3 | 0.843694539 | 0.906616328 | 0.103771381 | 0.547382999 | 0.662808897 |
| LINC02777 | 1.02434696 | 0.962877805 | -0.08927983 | 0.547383369 | 0.662808897 |
| MRPL20-AS1 | 8.117937852 | 8.509792697 | 0.068010692 | 0.547383369 | 0.662808897 |
| AL133467.4 | 0.43353752 | 0.867090321 | 1.000025425 | 0.549267868 | 0.664290655 |
| LINC00662 | 1.371875693 | 1.513886206 | 0.142107003 | 0.549941895 | 0.664290655 |
| AC018521.6 | 1.102045487 | 1.041952382 | -0.080894425 | 0.549941895 | 0.664290655 |
| OSTN-AS1 | 0.276988122 | 0.623908724 | 1.171510872 | 0.552695047 | 0.667076561 |
| AC131254.1 | 1.442387394 | 1.496786053 | 0.053409328 | 0.553775576 | 0.66784082 |
| AL023803.1 | 0.435677123 | 0.496100978 | 0.187374442 | 0.554622247 | 0.668322046 |
| LINC00665 | 2.471059812 | 2.647125555 | 0.099296693 | 0.555076558 | 0.668330081 |
| LINC01563 | 2.159936422 | 2.553569252 | 0.241526337 | 0.557644995 | 0.67088153 |
| ZEB1-AS1 | 0.761499794 | 0.800046364 | 0.071239962 | 0.558942867 | 0.671360982 |
| AC016876.2 | 6.929202631 | 6.894007288 | -0.007346522 | 0.558942867 | 0.671360982 |
| AC068338.3 | 0.519708919 | 0.467604336 | -0.15241551 | 0.563466869 | 0.675945808 |
| AC007032.1 | 0.399378308 | 0.412316675 | 0.045996832 | 0.564118113 | 0.675945808 |
| SNHG15 | 8.263133656 | 7.601545801 | -0.120396178 | 0.564118203 | 0.675945808 |
| AC003956.1 | 0.563776136 | 0.634148811 | 0.169699015 | 0.565408083 | 0.67694809 |
| AL031963.3 | 0.512070347 | 0.525513706 | 0.037386372 | 0.566714495 | 0.677968543 |
| AC012181.1 | 0.56446667 | 0.522160043 | -0.112396332 | 0.569315611 | 0.679991632 |
| AP001318.2 | 1.915419867 | 1.876677145 | -0.029480194 | 0.569316503 | 0.679991632 |
| SNHG14 | 1.174406793 | 1.272190207 | 0.115382168 | 0.570619643 | 0.681003299 |
| AC010186.3 | 1.606567119 | 1.489392794 | -0.109256973 | 0.574537563 | 0.685131455 |
| AC046134.2 | 0.671518837 | 0.663946665 | -0.016360516 | 0.577156563 | 0.687705306 |
| SUCLA2-AS1 | 0.75607133 | 0.734013192 | -0.042716357 | 0.578468168 | 0.688169701 |
| AL122035.1 | 1.268241895 | 1.442384234 | 0.185625591 | 0.578468168 | 0.688169701 |
| RHPN1-AS1 | 2.515725759 | 2.280450804 | -0.141655614 | 0.579781173 | 0.688552137 |
| AC009283.1 | 2.879714845 | 2.512220764 | -0.196962712 | 0.581095574 | 0.688552137 |
| HCG18 | 2.449332074 | 2.477793786 | 0.01666774 | 0.581095574 | 0.688552137 |
| AC245140.2 | 0.651289298 | 0.650701928 | -0.001301692 | 0.581095574 | 0.688552137 |
| AC004854.2 | 0.628421906 | 0.61700618 | -0.026448532 | 0.581095574 | 0.688552137 |
| AC004148.1 | 1.426368466 | 1.333062538 | -0.09760225 | 0.582411368 | 0.689563975 |
| AC138028.4 | 0.701832935 | 0.710698631 | 0.018110268 | 0.586367071 | 0.693413348 |
| AC104971.3 | 0.816099385 | 0.304685126 | -1.421425781 | 0.586591466 | 0.693413348 |
| AC007938.3 | 0.497663819 | 0.602191155 | 0.275050012 | 0.589010763 | 0.694623684 |
| PVT1 | 2.682300675 | 2.424419389 | -0.145831681 | 0.589011108 | 0.694623684 |
| AC005785.1 | 0.457156735 | 0.449733566 | -0.023618309 | 0.589011108 | 0.694623684 |
| AC060780.1 | 1.72295955 | 1.723425709 | 0.000390279 | 0.591660634 | 0.697110439 |
| AC018752.1 | 0.688305356 | 0.714943691 | 0.054780883 | 0.592814357 | 0.697110439 |
| PIK3CD-AS2 | 11.35379413 | 12.02772259 | 0.083189012 | 0.592987446 | 0.697110439 |
| AF117829.1 | 0.573336275 | 0.579318923 | 0.014976229 | 0.592987446 | 0.697110439 |
| CD2BP2-DT | 2.437262121 | 2.551424777 | 0.066041691 | 0.594315621 | 0.697488796 |
| AC074032.1 | 0.556092833 | 0.531605391 | -0.064970006 | 0.594315621 | 0.697488796 |
| LINC01739 | 1.245998543 | 1.672295342 | 0.424527281 | 0.59526243 | 0.697488796 |
| AC090425.2 | 0.636865798 | 0.705212283 | 0.147068206 | 0.595587325 | 0.697488796 |
| EPB41L4A-AS1 | 7.788183335 | 7.42834794 | -0.068245454 | 0.595645154 | 0.697488796 |
| AC018529.2 | 0.95124876 | 0.93484018 | -0.025102925 | 0.596976041 | 0.697870517 |
| CFAP58-DT | 0.475962737 | 0.595574817 | 0.323434123 | 0.598289671 | 0.697870517 |
| AC069234.5 | 0.442121733 | 0.422513735 | -0.065445413 | 0.598306588 | 0.697870517 |
| MIR762HG | 0.68666934 | 0.66449015 | -0.047367736 | 0.59830828 | 0.697870517 |
| SNHG29 | 95.67294384 | 91.33158361 | -0.066997142 | 0.59830828 | 0.697870517 |
| LINC00327 | 1.00411254 | 0.856303161 | -0.229727418 | 0.598974821 | 0.698102582 |
| LINC02732 | 7.30384169 | 8.636208301 | 0.241742545 | 0.602289069 | 0.701417769 |
| AC011363.1 | 0.414506907 | 0.448452154 | 0.113557928 | 0.607583081 | 0.707031598 |
| AC022150.4 | 0.473157559 | 0.526283979 | 0.153520803 | 0.613724912 | 0.713622503 |
| AC100786.1 | 1.854912959 | 1.956671552 | 0.077050114 | 0.614399245 | 0.71385064 |
| AC010331.1 | 0.398510309 | 0.42652942 | 0.098028211 | 0.617099177 | 0.715873782 |
| AL024507.3 | 0.412255027 | 0.431438101 | 0.065616504 | 0.617099502 | 0.715873782 |
| LINC00853 | 0.914632956 | 0.986808016 | 0.109576532 | 0.61912802 | 0.717669359 |
| AC093582.1 | 0.602042 | 0.638659476 | 0.085182773 | 0.627942565 | 0.726656168 |
| PCAT6 | 7.792330782 | 7.826507293 | 0.006313704 | 0.627952103 | 0.726656168 |
| AC009974.1 | 0.802443316 | 0.844987233 | 0.074530059 | 0.629312832 | 0.726656168 |
| AL121944.2 | 1.279180095 | 1.253776142 | -0.028939612 | 0.629314417 | 0.726656168 |
| AC005586.1 | 1.260977011 | 1.257780986 | -0.003661243 | 0.629314417 | 0.726656168 |
| AC103563.7 | 0.72571214 | 0.609336187 | -0.252158984 | 0.636203206 | 0.734042803 |
| AL353804.1 | 0.57710425 | 0.580084423 | 0.007430924 | 0.637513127 | 0.734986177 |
| HM13-IT1 | 1.018560842 | 1.140607972 | 0.163270861 | 0.638885754 | 0.736000332 |
| ARHGAP5-AS1 | 5.745812854 | 6.359762441 | 0.146461873 | 0.643006364 | 0.740176177 |
| AC093010.2 | 7.280129835 | 6.869174166 | -0.083827516 | 0.645759562 | 0.742200944 |
| CCNT2-AS1 | 0.940222231 | 0.872403656 | -0.108005977 | 0.645759562 | 0.742200944 |
| AL139241.1 | 1.029510455 | 0.532993023 | -0.949769928 | 0.647412788 | 0.743528687 |
| DYRK3-AS1 | 0.58492475 | 0.741622206 | 0.342433406 | 0.648511256 | 0.743653464 |
| AC092368.3 | 1.620354487 | 1.552080283 | -0.062106284 | 0.648517622 | 0.743653464 |
| AL162595.1 | 0.556666276 | 0.600709573 | 0.10985497 | 0.649207817 | 0.743873577 |
| LINC02328 | 0.649533957 | 0.643319337 | -0.013869897 | 0.649898465 | 0.744093871 |
| AC145423.3 | 0.720136588 | 0.736314355 | 0.032051262 | 0.651280512 | 0.745104831 |
| AC147067.1 | 1.214382631 | 1.23196589 | 0.02073925 | 0.654048199 | 0.747698286 |
| AC016888.1 | 1.084469079 | 1.212810157 | 0.161364821 | 0.656127019 | 0.749500872 |
| AC095057.3 | 0.733736387 | 0.586032051 | -0.324282262 | 0.659597099 | 0.751031485 |
| AC009403.1 | 1.68165744 | 1.646887914 | -0.030141483 | 0.659597836 | 0.751031485 |
| RAP2C-AS1 | 0.567131557 | 0.594435178 | 0.067836062 | 0.659597836 | 0.751031485 |
| AL662844.4 | 0.760365066 | 0.597680532 | -0.3473177 | 0.659597836 | 0.751031485 |
| LINC02449 | 0.462992117 | 0.351397885 | -0.397882124 | 0.660987458 | 0.751031485 |
| AC073046.1 | 0.873769069 | 0.890606917 | 0.02753678 | 0.660987899 | 0.751031485 |
| AC110285.2 | 3.936842109 | 3.818962877 | -0.043857957 | 0.660988193 | 0.751031485 |
| GAPLINC | 4.521314422 | 3.592678947 | -0.331682232 | 0.662379647 | 0.752040162 |
| AC068870.2 | 7.158657834 | 7.02898342 | -0.026373072 | 0.663772414 | 0.752477004 |
| AC007383.1 | 3.910451277 | 3.677666695 | -0.088544373 | 0.663772414 | 0.752477004 |
| LINC02604 | 3.286968831 | 3.021213801 | -0.121629494 | 0.666561286 | 0.75506525 |
| AL354733.3 | 0.785513091 | 0.716235398 | -0.133201497 | 0.667931184 | 0.75604341 |
| LEF1-AS1 | 0.794746043 | 0.724716197 | -0.133077789 | 0.669354773 | 0.757080816 |
| AC011239.1 | 0.290729436 | 0.688101259 | 1.242943733 | 0.670852626 | 0.758200583 |
| AC019171.1 | 0.463516374 | 0.793329025 | 0.77529903 | 0.676249736 | 0.762546766 |
| AC061992.2 | 0.719645064 | 0.824306941 | 0.195896114 | 0.676358468 | 0.762546766 |
| AC005519.1 | 0.417770602 | 0.428022996 | 0.034977332 | 0.677060322 | 0.762546766 |
| AC002553.2 | 0.577637878 | 0.584977242 | 0.01821515 | 0.677737005 | 0.762546766 |
| EXOC3-AS1 | 3.586143446 | 3.262759222 | -0.136340671 | 0.677762597 | 0.762546766 |
| AC254562.3 | 0.686538943 | 0.562401006 | -0.287742381 | 0.677762597 | 0.762546766 |
| GARS1-DT | 0.9690281 | 0.987531661 | 0.027288501 | 0.679167847 | 0.763552406 |
| AP002807.1 | 0.597250777 | 0.581609556 | -0.038285852 | 0.683390274 | 0.76757233 |
| INE1 | 0.875397134 | 0.833530334 | -0.070702958 | 0.684799962 | 0.76757233 |
| PARD6G-AS1 | 0.5371585 | 0.509603932 | -0.07597144 | 0.684799962 | 0.76757233 |
| TYMSOS | 2.100692188 | 2.023525547 | -0.053993717 | 0.684799962 | 0.76757233 |
| ZNF460-AS1 | 0.450136084 | 0.435062824 | -0.049137474 | 0.686208344 | 0.768573937 |
| AC083967.1 | 1.15504789 | 0.624871298 | -0.886321689 | 0.687958284 | 0.769956311 |
| AP003390.1 | 0.625660008 | 0.680701391 | 0.121643168 | 0.689031784 | 0.770007597 |
| BX322562.1 | 1.633749843 | 1.885092154 | 0.206447955 | 0.689035599 | 0.770007597 |
| AL137847.1 | 0.674242206 | 0.718127614 | 0.090973301 | 0.69041165 | 0.770286454 |
| UGDH-AS1 | 0.72998956 | 0.732966418 | 0.005871269 | 0.690449655 | 0.770286454 |
| LINC01094 | 0.581725908 | 0.668706453 | 0.201033482 | 0.691864793 | 0.770286454 |
| AC004988.1 | 5.537628178 | 5.731117963 | 0.049548404 | 0.691864793 | 0.770286454 |
| SNHG9 | 10.87113803 | 9.272533707 | -0.229467463 | 0.691864793 | 0.770286454 |
| VASH1-AS1 | 0.522960002 | 0.491239763 | -0.090273265 | 0.693281007 | 0.771288035 |
| AL021878.2 | 0.691930248 | 0.641002999 | -0.110295503 | 0.694698226 | 0.772289242 |
| AC004253.1 | 0.554416545 | 0.56469832 | 0.02651003 | 0.696114312 | 0.772715357 |
| AC091563.1 | 2.23747599 | 2.713815362 | 0.278450367 | 0.696116648 | 0.772715357 |
| AC092803.1 | 1.318689934 | 1.492989909 | 0.179099034 | 0.697536066 | 0.773715711 |
| AC036214.2 | 0.893572529 | 0.848859155 | -0.074059635 | 0.700378076 | 0.776291364 |
| PSMG3-AS1 | 2.910086847 | 3.090756055 | 0.086897582 | 0.701800658 | 0.777138312 |
| RBMS3-AS3 | 0.773521534 | 0.841526111 | 0.121566581 | 0.70314976 | 0.777138312 |
| LRRC8C-DT | 1.151832363 | 1.190178874 | 0.047247652 | 0.703224286 | 0.777138312 |
| AL606834.2 | 2.234515146 | 2.157251682 | -0.050767322 | 0.703224286 | 0.777138312 |
| AL031722.1 | 0.531027199 | 0.603729175 | 0.185115764 | 0.706743935 | 0.780450218 |
| AC079684.2 | 0.684679703 | 0.624249611 | -0.133306228 | 0.7075014 | 0.780709232 |
| LURAP1L-AS1 | 0.711581806 | 0.837005336 | 0.234207197 | 0.70887675 | 0.781127594 |
| AL445228.3 | 0.384433296 | 0.422133769 | 0.134966947 | 0.708926919 | 0.781127594 |
| AC093423.2 | 0.646879945 | 0.600920417 | -0.106324044 | 0.711067375 | 0.782908253 |
| AC009948.3 | 0.619697248 | 0.573697896 | -0.111272332 | 0.713215039 | 0.784694218 |
| LINC00668 | 0.456478877 | 0.431578494 | -0.080925126 | 0.713909936 | 0.784880364 |
| AL121655.1 | 0.416886698 | 0.41668392 | -0.000701912 | 0.716069442 | 0.786111849 |
| AL359091.4 | 1.122625027 | 1.172641368 | 0.062885731 | 0.716083131 | 0.786111849 |
| NDUFB2-AS1 | 0.817179375 | 0.810478666 | -0.011878581 | 0.717517095 | 0.787107291 |
| AC005696.3 | 0.457808949 | 0.415716383 | -0.139146058 | 0.718839041 | 0.787978479 |
| AC079305.2 | 0.541704763 | 0.676039972 | 0.319601774 | 0.720387506 | 0.788518267 |
| AC108010.1 | 1.32302434 | 1.377268507 | 0.057970245 | 0.720387754 | 0.788518267 |
| AL390760.1 | 1.091580742 | 1.628009664 | 0.576690417 | 0.72289455 | 0.790505639 |
| GAS5-AS1 | 1.09549039 | 1.107726581 | 0.016024999 | 0.72326236 | 0.790505639 |
| AC073073.2 | 0.604282761 | 0.632507607 | 0.065859047 | 0.726140875 | 0.793071197 |
| AC009118.3 | 1.008257875 | 1.064064379 | 0.077720767 | 0.72686105 | 0.793277447 |
| LINC01063 | 0.765544736 | 0.663075099 | -0.207314409 | 0.727580983 | 0.793483132 |
| AL050309.1 | 0.457019298 | 0.487324469 | 0.092627577 | 0.729023023 | 0.793896231 |
| AC024575.2 | 0.413936415 | 0.426137656 | 0.041910373 | 0.729023264 | 0.793896231 |
| AC009065.6 | 0.660467133 | 0.755544047 | 0.194029095 | 0.731906151 | 0.79587827 |
| MCM3AP-AS1 | 0.445566964 | 0.476309491 | 0.096257028 | 0.731909487 | 0.79587827 |
| LINC02608 | 0.59151076 | 0.702578533 | 0.248255084 | 0.733997953 | 0.797568373 |
| AC034243.1 | 0.575190353 | 0.533825218 | -0.107672021 | 0.734694965 | 0.797745151 |
| AC015922.3 | 6.686631839 | 6.224450998 | -0.10333309 | 0.736245933 | 0.798848239 |
| HAGLR | 6.138396863 | 6.568447768 | 0.097690556 | 0.737693291 | 0.799837388 |
| AC016355.1 | 0.507296914 | 0.522698109 | 0.043147554 | 0.742040932 | 0.803967425 |
| AC015712.2 | 0.589665373 | 0.724263637 | 0.296618467 | 0.743491985 | 0.804955427 |
| PRANCR | 2.151568328 | 1.285074614 | -0.743536529 | 0.74639682 | 0.807514821 |
| GEMIN7-AS1 | 0.581314631 | 0.628800178 | 0.113282406 | 0.750760827 | 0.810474269 |
| A1BG-AS1 | 1.707415893 | 1.72197656 | 0.012250992 | 0.750760827 | 0.810474269 |
| AC092687.3 | 1.165207571 | 1.265192074 | 0.118769442 | 0.750760827 | 0.810474269 |
| AL159166.1 | 8.012905455 | 8.711608962 | 0.120613743 | 0.752716078 | 0.811997908 |
| HCG25 | 0.575039121 | 0.582793629 | 0.019324997 | 0.756591906 | 0.815589687 |
| AL359962.1 | 0.696270287 | 0.886426748 | 0.348353958 | 0.759468698 | 0.817787004 |
| AL133304.2 | 0.744499332 | 0.524403686 | -0.505592729 | 0.75972577 | 0.817787004 |
| SREBF2-AS1 | 1.670401814 | 1.719347357 | 0.041665856 | 0.762436877 | 0.820114019 |
| AC069120.1 | 0.531433892 | 0.838151679 | 0.65732111 | 0.764076163 | 0.82128561 |
| AC109454.2 | 0.431903818 | 0.452451596 | 0.067053386 | 0.765364264 | 0.822078307 |
| AC093249.6 | 0.810253097 | 0.741349317 | -0.128219144 | 0.766829535 | 0.822468747 |
| AC010531.6 | 0.845538034 | 0.982048497 | 0.21592462 | 0.766829535 | 0.822468747 |
| AC084036.1 | 2.432858593 | 2.651280436 | 0.124036809 | 0.768295428 | 0.823449443 |
| AC093388.1 | 1.144707942 | 1.169280638 | 0.030641672 | 0.771229698 | 0.826001391 |
| AC011472.1 | 0.702425417 | 0.705377349 | 0.0060502 | 0.772698064 | 0.826388402 |
| AC009812.4 | 2.140319137 | 1.712542322 | -0.321686287 | 0.772698064 | 0.826388402 |
| AL391834.2 | 1.582964978 | 1.530694349 | -0.048443105 | 0.774167244 | 0.827366998 |
| AC034236.2 | 0.906679198 | 0.89365514 | -0.02087398 | 0.775637234 | 0.828345057 |
| DBH-AS1 | 0.877503944 | 0.736850589 | -0.252033496 | 0.777107827 | 0.829322363 |
| LINC02055 | 0.565766609 | 0.652341222 | 0.205419765 | 0.78157504 | 0.833493953 |
| AC005291.1 | 2.015691556 | 1.905503711 | -0.081102475 | 0.783619457 | 0.835077694 |
| AC107294.2 | 0.696751515 | 0.738153546 | 0.083276713 | 0.78594892 | 0.836366684 |
| AC010761.1 | 1.588121726 | 1.518117596 | -0.065037947 | 0.785949402 | 0.836366684 |
| AL078590.2 | 1.420680159 | 1.432671308 | 0.012125862 | 0.787425495 | 0.836744875 |
| AC002553.1 | 0.568459518 | 0.544807536 | -0.061310953 | 0.787425687 | 0.836744875 |
| AC004923.4 | 0.445985199 | 0.381674448 | -0.224653227 | 0.791859104 | 0.840259874 |
| BX537318.1 | 1.079113372 | 1.10274391 | 0.031251349 | 0.791859104 | 0.840259874 |
| LINC01436 | 3.417317071 | 4.396440163 | 0.363471721 | 0.799114112 | 0.847356086 |
| SNHG32 | 86.30695326 | 75.50901498 | -0.192827897 | 0.803714137 | 0.851628961 |
| STX17-AS1 | 0.504634368 | 0.504731327 | 0.000277168 | 0.805198815 | 0.852597044 |
| AC116036.2 | 0.418596158 | 0.440144347 | 0.072417671 | 0.806677852 | 0.852955365 |
| AL121601.1 | 0.508353902 | 0.50815474 | -0.000565327 | 0.806679823 | 0.852955365 |
| SNHG28 | 0.558105698 | 0.575988649 | 0.045502005 | 0.808171601 | 0.853927955 |
| AGBL5-IT1 | 0.809640042 | 0.764101895 | -0.083515605 | 0.811890881 | 0.856825214 |
| AL928654.2 | 1.728905009 | 1.711119756 | -0.014917872 | 0.81263513 | 0.856825214 |
| ERVK13-1 | 0.689528975 | 0.615448945 | -0.163971995 | 0.8126353 | 0.856825214 |
| AL008582.1 | 0.410969311 | 0.390652091 | -0.073146329 | 0.817100112 | 0.860924818 |
| AL133553.1 | 0.54628069 | 0.697908767 | 0.353396028 | 0.818155518 | 0.861428906 |
| AC003035.2 | 0.410314264 | 0.689906331 | 0.749671191 | 0.818942062 | 0.861640955 |
| RBM26-AS1 | 0.534005205 | 0.577564864 | 0.113129175 | 0.820088277 | 0.861640955 |
| AC009065.9 | 0.911438681 | 0.947968399 | 0.056693368 | 0.820088277 | 0.861640955 |
| AC104532.2 | 0.422942903 | 0.449305153 | 0.087232695 | 0.82755534 | 0.868874911 |
| OSER1-DT | 6.719475946 | 6.714441847 | -0.001081244 | 0.833544231 | 0.874547812 |
| SLC12A9-AS1 | 1.09170747 | 1.128587622 | 0.047932104 | 0.835042428 | 0.875504455 |
| AC074135.1 | 0.407721053 | 0.395652094 | -0.043350059 | 0.836010024 | 0.875845755 |
| AC018904.1 | 3.003621977 | 2.935019306 | -0.03333326 | 0.836541224 | 0.875845755 |
| CHL1-AS2 | 1.789938933 | 1.89242319 | 0.080324376 | 0.837225123 | 0.875947519 |
| AC103974.1 | 0.776372807 | 0.920714743 | 0.246004661 | 0.841791613 | 0.879661286 |
| AC003102.1 | 0.584187508 | 0.487024542 | -0.262437032 | 0.84254229 | 0.879661286 |
| AC002467.1 | 2.146124769 | 2.234405878 | 0.058157321 | 0.84254229 | 0.879661286 |
| ST7-AS1 | 1.270866541 | 1.307646013 | 0.041159513 | 0.845546279 | 0.881899815 |
| AC243960.3 | 0.490488794 | 0.443760222 | -0.144439825 | 0.847009293 | 0.881899815 |
| LINC00863 | 0.56589311 | 0.547172666 | -0.04853341 | 0.847049119 | 0.881899815 |
| LINC00667 | 4.459614188 | 4.671993328 | 0.067119309 | 0.847049119 | 0.881899815 |
| AL133325.3 | 0.476343825 | 0.462859852 | -0.041427856 | 0.848550578 | 0.882234613 |
| AC026401.3 | 13.66238428 | 14.52374467 | 0.088204196 | 0.848552515 | 0.882234613 |
| EIF2AK3-DT | 0.545179023 | 0.567544575 | 0.058003653 | 0.863615906 | 0.896647112 |
| AL031775.1 | 1.097829333 | 1.088447582 | -0.012381861 | 0.863615906 | 0.896647112 |
| AP002360.2 | 6.598603836 | 6.04840824 | -0.125605286 | 0.866634725 | 0.899156112 |
| AC012313.2 | 0.986102143 | 1.002628278 | 0.023977833 | 0.868144868 | 0.900097422 |
| AC017100.1 | 5.166741201 | 4.869374151 | -0.085518264 | 0.869655492 | 0.900413071 |
| AC022706.1 | 0.697405813 | 0.529475993 | -0.397433117 | 0.869655492 | 0.900413071 |
| AC006942.1 | 1.17334144 | 1.218403887 | 0.054369553 | 0.875702691 | 0.904791777 |
| AC021755.2 | 0.550456514 | 0.671645882 | 0.287072192 | 0.875702691 | 0.904791777 |
| AC124016.2 | 0.700570148 | 0.67672795 | -0.04995354 | 0.875702691 | 0.904791777 |
| AC007066.2 | 0.485779704 | 0.476004136 | -0.029328108 | 0.877215639 | 0.905102246 |
| GTSE1-DT | 0.473862216 | 0.473061366 | -0.002440285 | 0.877215639 | 0.905102246 |
| LINC00482 | 1.030677152 | 1.128180474 | 0.130405377 | 0.877972254 | 0.905257304 |
| AL139246.5 | 4.255211071 | 6.05340514 | 0.508516215 | 0.881757156 | 0.90853239 |
| AL136295.7 | 1.527432093 | 1.450069102 | -0.074986589 | 0.892368905 | 0.918832259 |
| ZNF213-AS1 | 1.961078263 | 1.923319297 | -0.028048823 | 0.893886489 | 0.919760529 |
| EPHA5-AS1 | 0.494521686 | 0.498074272 | 0.010327102 | 0.898274422 | 0.92363892 |
| LOH12CR2 | 0.959278652 | 0.965013254 | 0.008598807 | 0.901480128 | 0.925660132 |
| MIR181A2HG | 1.017614975 | 1.215111885 | 0.255897353 | 0.901480128 | 0.925660132 |
| AC022916.2 | 0.636034848 | 0.602189016 | -0.07888942 | 0.906040689 | 0.929065074 |
| AL118516.1 | 6.494984717 | 5.908323435 | -0.136577326 | 0.906040689 | 0.929065074 |
| AC004816.1 | 0.725127138 | 0.766180663 | 0.079450647 | 0.909073271 | 0.93153493 |
| OCIAD1-AS1 | 0.528515439 | 0.502431427 | -0.073018907 | 0.910587552 | 0.93244665 |
| AC012146.1 | 2.161349158 | 2.57664517 | 0.253561724 | 0.913648438 | 0.934939766 |
| AP000282.1 | 0.538608821 | 0.731225989 | 0.44107949 | 0.914301094 | 0.934966804 |
| AC022509.2 | 0.527793991 | 0.597523904 | 0.179021505 | 0.91517057 | 0.935215374 |
| AP001107.9 | 0.891967599 | 0.907529383 | 0.02495305 | 0.916693795 | 0.936131215 |
| LINC02620 | 0.865352884 | 0.925146306 | 0.096392965 | 0.918970123 | 0.93781435 |
| AC010359.2 | 0.578806659 | 0.583269947 | 0.011082221 | 0.924312422 | 0.941978479 |
| UBAC2-AS1 | 0.911674436 | 0.966062862 | 0.083598346 | 0.92431244 | 0.941978479 |
| AC006206.2 | 0.429415977 | 0.40815735 | -0.073250432 | 0.928223514 | 0.945319036 |
| SLCO4A1-AS1 | 20.0347284 | 20.54950816 | 0.036600912 | 0.931938033 | 0.948454999 |
| AC104958.2 | 1.897863198 | 1.663980826 | -0.189737194 | 0.938043043 | 0.954017891 |
| AC012615.6 | 0.613793389 | 0.602383036 | -0.027071964 | 0.942624212 | 0.958024471 |
| MIR100HG | 2.736438477 | 2.839734004 | 0.053456379 | 0.947207279 | 0.961373533 |
| AC102953.2 | 1.066517101 | 1.44417036 | 0.43733384 | 0.947207279 | 0.961373533 |
| AL645568.1 | 0.475626736 | 0.432376036 | -0.137543251 | 0.951792094 | 0.965370649 |
| AC022364.1 | 1.23691319 | 1.164452673 | -0.087092247 | 0.953320728 | 0.966264661 |
| AC078880.3 | 1.601081969 | 2.494830337 | 0.639894538 | 0.95862929 | 0.970986112 |
| AL034376.1 | 15.35216863 | 21.2906949 | 0.471780574 | 0.964020007 | 0.975784318 |
| AC244090.1 | 8.416516568 | 8.587022412 | 0.028934704 | 0.967085487 | 0.978224005 |
| AC100810.1 | 8.163806861 | 8.41650012 | 0.043978383 | 0.968615585 | 0.979108374 |
| AP001462.1 | 0.550384263 | 0.502643604 | -0.13090339 | 0.970145799 | 0.979991663 |
| AC027020.2 | 0.888252114 | 0.943036866 | 0.086344953 | 0.971676123 | 0.980873869 |
| AC005840.4 | 0.58060406 | 0.508342719 | -0.191753186 | 0.973206552 | 0.981754988 |
| LINC02765 | 1.136294696 | 1.475679962 | 0.377042827 | 0.975458271 | 0.983362051 |
| AL109917.1 | 0.431578303 | 0.53752177 | 0.316700848 | 0.977798401 | 0.985056014 |
| AC110285.5 | 0.400448389 | 0.467277898 | 0.222664484 | 0.980077787 | 0.986686538 |
| AP001486.2 | 0.58058639 | 0.615208444 | 0.083564554 | 0.98086006 | 0.986808673 |
| LINC00653 | 0.822809485 | 0.726518859 | -0.179558176 | 0.983921997 | 0.989222587 |
| AC091979.1 | 1.255733761 | 1.427439784 | 0.184899269 | 0.985358945 | 0.990000609 |
| CAHM | 0.556012374 | 0.573029038 | 0.04349126 | 0.988515331 | 0.991836955 |
| IQCH-AS1 | 0.71519292 | 0.648643463 | -0.140906758 | 0.988515331 | 0.991836955 |
| AC139530.1 | 2.076117128 | 2.057351569 | -0.01309949 | 0.990046535 | 0.992706163 |
| AP001816.1 | 4.587110047 | 4.482637076 | -0.033237818 | 0.991577775 | 0.99357424 |
| AC012184.3 | 0.585643155 | 0.63492037 | 0.116553798 | 0.99617166 | 0.997507906 |
| GLIDR | 0.71921202 | 0.724087963 | 0.009747836 | 0.99770299 | 0.998371692 |
| ALOX12-AS1 | 1.155388468 | 1.141163075 | -0.017873029 | 1 | 1 |
