# Supplementary figures and images for: Gene Instability-Related lncRNA Prognostic Model of Melanoma Patients via Machine Learning Strategy
Source: J Oncol. 2021 May 25;2021:5582920. doi: 10.1155/2021/5582920 (PMC8169244; doi:10.1155/2021/5582920)

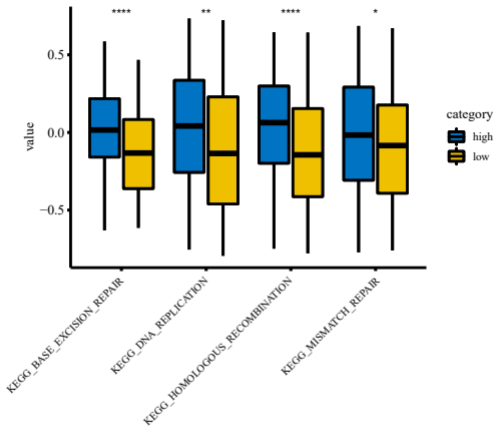

Supplement: Supplementary Materials — Supplemental information for this article can be found online. Figure S1: the following four KEGG pathways (ssGSEA) associated with gene instability were analyzed for characteristic differences between the high- and low-risk score groups: base excision repair, DNA replication, homologous recombination, and mismatch repair. ∗∗∗∗P < 0.0001. ∗∗P < 0.01. ∗P < 0.05. Table S1: the difference analysis of the matrix lncRNA. [file 5582920.f1.zip › 5582920.f1/supplementary figure 1 (1).pdf]
